# Live Cell Analysis and Mathematical Modeling Identify Determinants of Attenuation of Dengue Virus 2’-O-Methylation Mutant

**DOI:** 10.1371/journal.ppat.1005345

**Published:** 2015-12-31

**Authors:** Bianca Schmid, Melanie Rinas, Alessia Ruggieri, Eliana Gisela Acosta, Marie Bartenschlager, Antje Reuter, Wolfgang Fischl, Nathalie Harder, Jan-Philip Bergeest, Michael Flossdorf, Karl Rohr, Thomas Höfer, Ralf Bartenschlager

**Affiliations:** 1 Department of Infectious Diseases, Molecular Virology, University of Heidelberg, Heidelberg, Germany; 2 Division of Theoretical Systems Biology, German Cancer Research Center (DKFZ), Heidelberg, Germany; 3 BioQuant Center, University of Heidelberg, Heidelberg, Germany; 4 Department of Bioinformatics and Functional Genomics, Biomedical Computer Vision Group, Institute of Pharmacy and Molecular Biotechnology, University of Heidelberg, Heidelberg, Germany; Purdue University, UNITED STATES

## Abstract

Dengue virus (DENV) is the most common mosquito-transmitted virus infecting ~390 million people worldwide. In spite of this high medical relevance, neither a vaccine nor antiviral therapy is currently available. DENV elicits a strong interferon (IFN) response in infected cells, but at the same time actively counteracts IFN production and signaling. Although the kinetics of activation of this innate antiviral defense and the timing of viral counteraction critically determine the magnitude of infection and thus disease, quantitative and kinetic analyses are lacking and it remains poorly understood how DENV spreads in IFN-competent cell systems. To dissect the dynamics of replication versus antiviral defense at the single cell level, we generated a fully viable reporter DENV and host cells with authentic reporters for IFN-stimulated antiviral genes. We find that IFN controls DENV infection in a kinetically determined manner that at the single cell level is highly heterogeneous and stochastic. Even at high-dose, IFN does not fully protect all cells in the culture and, therefore, viral spread occurs even in the face of antiviral protection of naïve cells by IFN. By contrast, a vaccine candidate DENV mutant, which lacks 2’-O-methylation of viral RNA is profoundly attenuated in IFN-competent cells. Through mathematical modeling of time-resolved data and validation experiments we show that the primary determinant for attenuation is the accelerated kinetics of IFN production. This rapid induction triggered by mutant DENV precedes establishment of IFN-resistance in infected cells, thus causing a massive reduction of virus production rate. In contrast, accelerated protection of naïve cells by paracrine IFN action has negligible impact. In conclusion, these results show that attenuation of the 2’-O-methylation DENV mutant is primarily determined by kinetics of autocrine IFN action on infected cells.

## Author Summary

Dengue virus (DENV) infection is a global health problem for which no selective therapy or vaccine exists. The magnitude of infection critically depends on the induction kinetics of the interferon (IFN) response and the kinetics of viral countermeasures. Here we established a novel live cell imaging system to dissect the dynamics of this interplay. We find that IFN controls DENV infection in a kinetically determined manner. At the single cell level, the IFN response is highly heterogeneous and stochastic, likely accounting for viral spread in the presence of IFN. Mathematical modeling and validation experiments show that the kinetics of activation of the IFN response critically determines control of virus replication and spread. A vaccine candidate DENV mutant lacking 2’-O-methylation of viral RNA is profoundly attenuated in IFN-competent cells. This attenuation is primarily due to accelerated kinetics of IFN production acting on infected cells in an autocrine manner. In contrast, accelerated protection of naïve cells by paracrine IFN action has negligible impact. Thus, attenuation of the 2’-O-methylation DENV mutant is primarily determined by kinetics of autocrine IFN action on infected cells.

## Introduction

Dengue virus (DENV) is a mosquito-transmitted pathogen infecting ~390 million people each year [1]. In ~500,000 cases, predominantly in children, the infection leads to more severe disease characterized by vascular leakage and hypovolemic shock [2,3]. As vector control methods are inefficient and neither approved vaccines nor antiviral therapies are available, DENV infections are an unmet global health problem [1,4].

The five serotypes of DENV belong to the genus *Flavivirus* [5] and have a capped single-stranded RNA genome of positive polarity. The genome encodes for a polyprotein that is cleaved proteolytically into three structural proteins (capsid protein, prM and envelope) and seven non-structural proteins (NS1, NS2A, NS2B, NS3, NS4A, NS4B and NS5; [6,7]). The NS proteins are required for viral RNA replication in the cytoplasm in close association with intracellular membranes [8,9].

DENV is recognized by the innate immune system of the human host. During DENV replication, double-stranded viral RNA is sensed by the pattern recognition receptors (PRRs) RIG-I (retinoic acid inducible gene I) and Mda5 (Melanoma differentiation-associated protein 5) [10–12]. Their activation induces the expression of type 1 interferons (IFN-α and IFN-β) and type 3 IFNs (IFN λ1, λ2 and λ3, also referred to as IL29, IL28A and IL28B, respectively, and IFN-λ4) [13–17]. Upon release from infected cells, IFNs signal in an autocrine and paracrine manner, causing the expression of antiviral IFN-stimulated genes (ISGs) in target cells via phosphorylation of STAT1 and STAT2 [18–20]. ISGs block virus replication in the cytoplasm at multiple steps [21].

DENV counteracts the IFN response through several mechanisms (reviewed in [7]). A key mediator of IFN escape is NS5 that contains a C-terminal RNA-dependent RNA polymerase (RdRp) and N-terminal RNA methyl-transferase (MTase) and guanylyl-transferase (GTase) activities. The latter two enzymatic activities mediate the capping of the DENV RNA genome in the cytoplasm [22,23], where cellular orthologs of these enzymes are not available [24,25], as well as internal RNA methylation [26]. The latter activity affects the 2’-OH group of the ribose and impedes both the detection of viral RNA by PRRs and its sequestration by the ISG IFIT1 (IFN-induced protein with tetratricopeptide repeats 1) [27–33]. In addition, NS5 induces the proteasomal degradation of STAT2 [34–37] via the interacting host cell protein UBR4 (ubiquitin protein ligase E3 component n-recognin 4) [37], thus preventing ISG induction by IFN.

Hence, on one hand, DENV impairs the host’s innate immune response by inhibiting the induction of IFNs, abrogating IFN-induced signaling through the JAK-STAT1/2 pathway, and escaping the action of ISGs. On the other hand, DENV potently induces IFNs and other antiviral cytokines and is sensitive to multiple ISGs whose expression confers protection against DENV infection [21,38]. Interestingly, the abrogation of a single escape mechanism, namely the 2’-O-methylation of the viral RNA, strongly attenuates the closely related West Nile virus *in vivo* [27]. Therefore, DENV mutants unable to perform 2’-O-methylation have been proposed as vaccine candidates [39,40]. However, the mechanisms by which viral 2’-O-methylation mutants of DENV are attenuated are not clear. For the West Nile virus, Daffis and co-workers [27], in agreement with further studies [28,31,41], have described sequestration of mutant virus RNA by IFIT1, but did not detect enhanced IFN expression in response to mutant virus. By contrast, Zust and colleagues, who worked with human and mouse coronaviruses, observed higher type 1 IFN expression induced by mutants lacking 2'-O-methyltransferase activity [29].

These contrasting findings, along with the potential medical relevance of the DENV 2'-O-methylation mutant, beg for a mechanistic explanation of how wildtype DENV spreads in IFN-competent host cells and why the mutant is attenuated. Previous studies reported the sensitivity of DENV replication and virus production to IFN treatment or impairment by IFN-effector proteins under steady-state conditions [34,38,42,43] or visualized either only DENV replication [44] or only the IFN response to a non-spreading virus [45]. Here, we have established a live cell imaging system to monitor simultaneously the dynamics of DENV spread and IFN response and quantified the interplay of these two processes by data-driven mathematical modeling. Our results reveal an early window of opportunity during which the IFN response can curb DENV replication by acting on already infected cells. Thus, the timing of IFN induction is a decisive factor of viral replication and spread that critically determines attenuation of the 2'-O-methylation mutant.

## Results

### IFN pre-treatment renders cultured cells resistant against DENV infection

**Fig 1:**
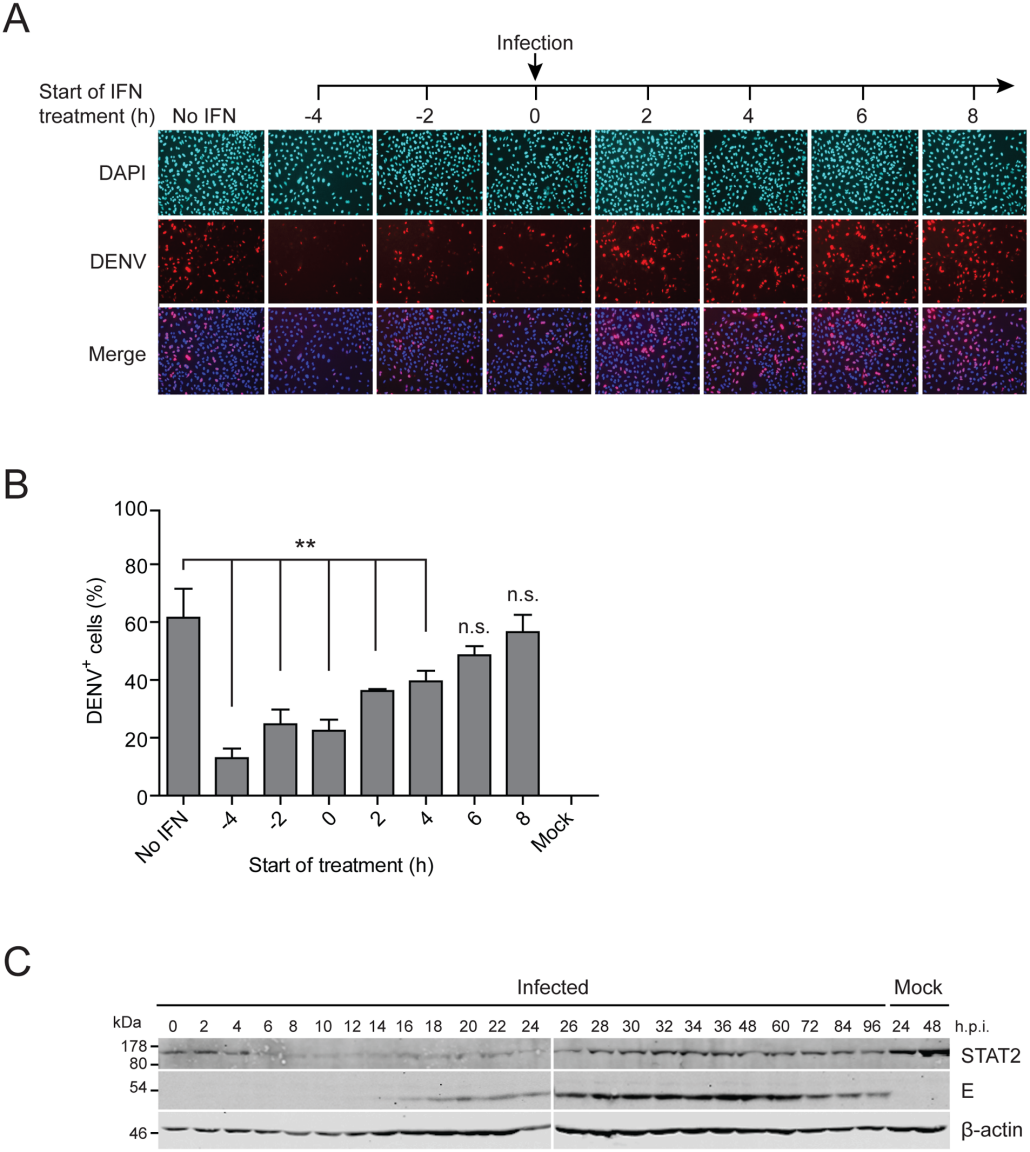
IFN pre-treatment protects against DENV infection. (**A**) IFN-competent A549 cells were treated with 100 IU/ml IFN-α prior to (-4 h, -2 h) or after (2 h, 4 h, 6 h, 8 h) or at the time point of infection (0 h) with the DENV2 strain 16681 at a MOI of 10 TCID_50_/cell. Cells were fixed 24 h post infection and analyzed by immunofluorescence using a NS5-specific antiserum. Mock-treated and DENV-infected cells without IFN treatment served as reference. (**B**) Quantification of infection efficiency of cells shown in panel (A). For each time point, 500–1,000 cells detected in 3 view fields were analyzed for DENV infection. Mean values and SDs are shown for each time point (** p-value <0.005). (**C**) IFN-competent A549 cells were infected with the DENV2 strain 16681 at a MOI of 10 TCID_50_/cell. Cells were harvested at given time points after infection (h p.i.) and lysates were analyzed by Western blot using STAT2-, E- (DENV envelope protein) and β-actin-specific antisera. A representative immunoblot from 3 independent experiments is shown. Numbers in the left refer to the positions of molecular weight standards in kiloDalton (kDa).

We reasoned that the efficiency of virus spread and ultimately the outcome of infection should depend on the dynamics of virus replication versus activation of the IFN response. To study the dynamics of this interplay we utilized A549 cells that are permissive for DENV and capable of producing type 1 and 3 IFNs. In the first set of experiments, we determined the time-dependency of DENV-2 (strain 16681) IFN sensitivity by treating cells with 100 IU/ml IFN-α prior to (-4 and -2 h) or after DENV infection (2, 4, 6 and 8 h) with MOI 10 TCID_50_/cell. Cells were fixed 24 h post infection and the fraction of NS5-expressing cells was determined by immunofluorescence (Fig 1A and 1B). In the absence of IFN treatment, ~60% of the cells were infected. However, pre-treatment of cells with IFN-α 4 h prior to infection reduced the number of DENV-positive cells to ~15%. This number steadily increased when IFN treatment was delayed until 6 h post infection and, after this time point, inhibition of DENV replication was no longer detectable. A similar time-dependent IFN sensitivity was observed with the same concentration of IFN-α after DENV infection with MOI 0.1 TCID_50_/cell (S1A Fig), a condition used later on in live cell imaging. Measurement of the titers of virus released from cells revealed a ~2 log decrease when cells were treated with IFN-α prior to or until 2 h after infection (S1B Fig).

To correlate DENV replication and STAT2 degradation, we infected A549 cells with the virus and harvested the cells right after inoculation up to 96 h post infection. In agreement with earlier reports [34], Western blot analysis revealed rapid STAT2 degradation that was detectable already 6 h after infection (Fig 1C). From 26 h post infection onwards STAT2 abundance increased again, most likely due to continued growth of uninfected cells expressing normal levels of STAT2 (~40% as deduced from the infection efficiency of cells that had not been treated with IFN; see Fig 1B). In addition, as STAT2 is an ISG, the increase of this protein might be due to IFN released from infected cells and enhancing expression of STAT2 in cells responding to IFN.

In summary, these data show that IFN curbs DENV infection, yet the virus efficiently spreads in IFN-competent A549 cells. As the effectiveness of IFN depended on the time of its delivery relative to the infection, these results suggested that the kinetics of virus replication and spread versus kinetics of activation of the IFN response likely determine the outcome of DENV infection.

### Establishment and characterization of reporter cells suitable to monitor IFN response at the single cell level and in live cells

**Fig 2:**
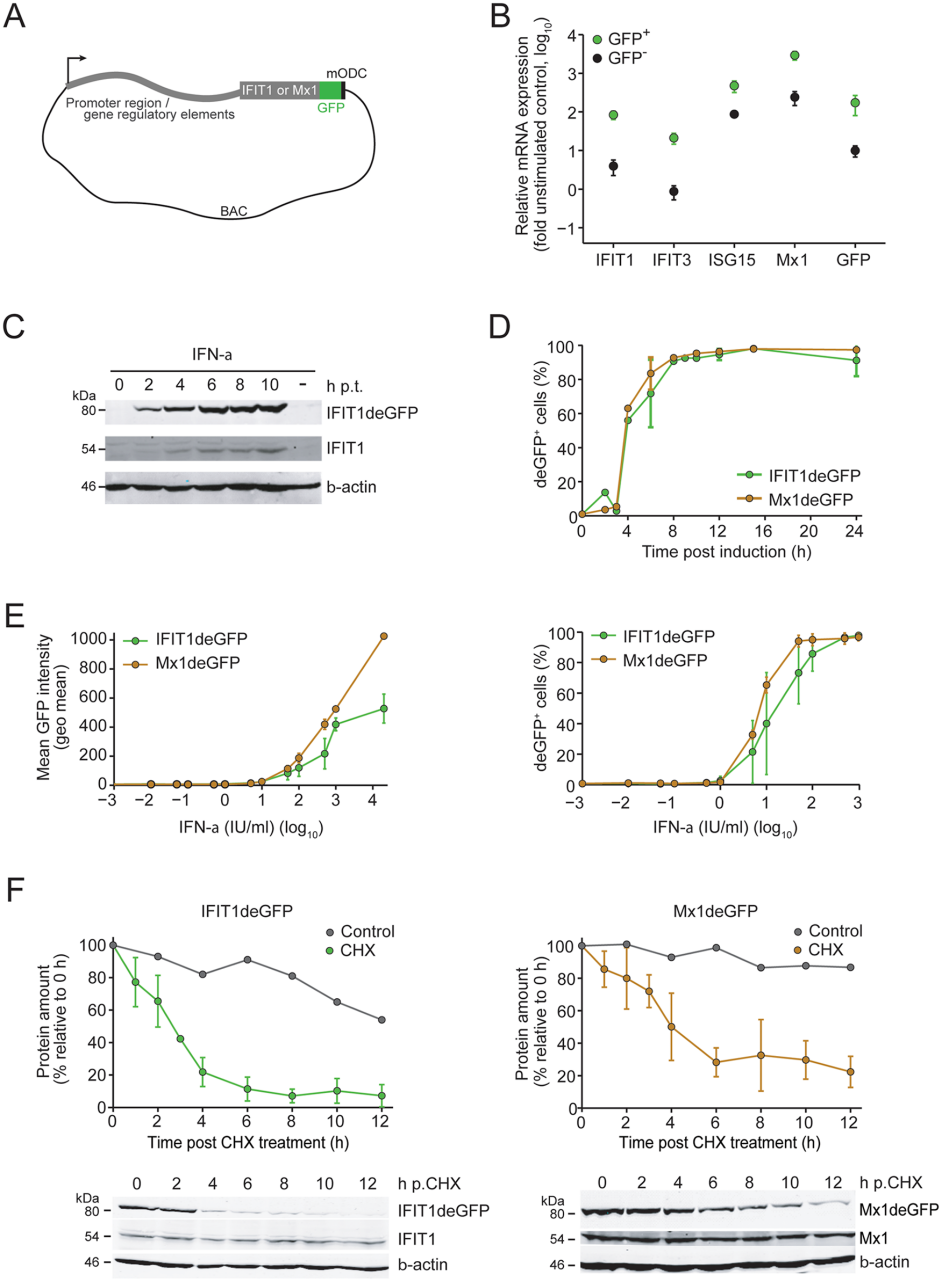
Characterization of stable BAC reporter cell lines. (**A**) Schematic of the BAC reporter construct. Destabilization of the Mx1deGFP or IFIT1deGFP fusion protein was achieved by C-terminal fusion with the degradation domain of mouse ornithine decarboxylase (mODC). (**B**) A549-IFIT1deGFP cells were stimulated with 10 IU/ml IFN-α for 24 h and subjected to FACS to separate cells according to expression of IFIT1deGFP. Right after sorting, GFP-positive and -negative cells were lysed and total RNA was extracted. Amounts of mRNAs specified in the bottom of the graph were quantified by RT-qPCR and normalized to GAPDH mRNA levels. Note that the IFIT1 specific RT-qPCR detected both the endogenous ISG and the reporter mRNA. Data represent the mean from two independent experiments and their respective SDs. (**C**) A549-IFIT1deGFP cells were stimulated with 100 IU/ml IFN-α (to achieve high level expression of the endogenous ISG), harvested at time points specified in the top (hours) and analyzed by Western blot to detect proteins specified in the right. A representative blot from 3 independent experiments is shown. Mock-treated cells are shown in the right lane of each panel. (**D**) Induction kinetics of IFIT1deGFP and Mx1deGFP after stimulation of A549 reporter cell lines with IFN-α. Cells were treated with 100 IU/ml of IFN-α, harvested at time points specified in the bottom of the graph and number of GFP-positive cells was determined by flow cytometry. Data are mean from 3 independent experiments and their respective SDs. (**E**) IFN-α dose response assay with A549 reporter cell lines. Cells were stimulated for 24 h with 100 IU/ml IFN-α and analyzed for mean GFP intensity (left panel) or number of GFP-expressing cells (right panel) by using flow cytometry. Data are mean from 3 independent experiments and their respective SDs. (**F**) Half-life of IFIT1deGFP (left panel) and Mx1deGFP (right panel) as determined by CHX treatment of cells after pre-stimulation with 100 IU/ml IFN-α for 15 h. Cells were harvested at time points given in the bottom of the graph and cell lysates were analyzed by Western blot using GFP-, IFIT1-, Mx1- and β-actin-specific antisera. Representative blots are shown in the bottom of each panel; quantifications from 4 independent experiments and their respective SDs are depicted in the upper graphs of each panel. In panels C and F, numbers in the left of Western blots refer to molecular weights of size standards in kiloDalton (kDa), respectively.

In order to monitor the dynamics of the IFN response as well as DENV replication and spread in real time and at the single cell level, we established IFN-competent A549 reporter cell lines. Cells were stably transfected with modified genes encoding for the ISGs Mx1 or IFIT1 contained in bacterial artificial chromosomes (BACs). This system allowed the expression of the respective ISGs under control of the authentic gene regulatory elements (used BACs contained 49,417 bp and 84,599 bp upstream of the coding sequence of Mx1 and IFIT1, respectively; Fig 2A). To enable the detection of Mx1 and IFIT1, they were each fused in-frame to the destabilized enhanced green fluorescent protein (deGFP) containing C-terminally the degradation domain of mouse ornithine decarboxylase that was used to reduce stability of the respective ISG (see below). Characterization of stable cell lines A549-Mx1deGFP (S2 Fig) and A549-IFIT1deGFP (Fig 2B) revealed that IFN-treated and deGFP-positive cells indeed contained significantly higher amounts of ISG mRNAs as compared to deGFP-negative cells. Moreover, treatment of A549-IFIT1 reporter cells with IFN-α (Fig 2C) or IFN-λ (S3A Fig) showed comparable induction kinetics of the deGFP reporter protein and the corresponding endogenous ISG. Application of biologically equipotent concentrations of IFN-α or IFN-λ as determined by hepatitis C virus (HCV) replicon inhibition assay [46], revealed comparable induction kinetics of IFIT1deGFP and Mx1deGFP (Fig 2D and S3B Fig, respectively). In addition, induction of reporter ISGs was dose-dependent both with respect to mean deGFP expression (Fig 2E, left panel) and fraction of deGFP-positive cells (Fig 2E, right panel). Analogous results were obtained for IFN-λ treatment (S3C Fig).

Kinetic analyses of biological responses require reporter proteins with rather short half-lives. Both IFIT1 and Mx1, are very stable (e.g. *t*_*1/2*_ of the mouse MxA is ~2.3 days [47] and *t*_*1/2*_ of IFIT1 >24 h; A. Pichlmair, personal communication). For this reason, we fused these genes C-terminally with deGFP (Fig 2A) and assessed the stability of these fusion proteins by using cycloheximide (CHX) treatment of cells [13,48]. Stable A549 reporter cells were treated with IFN-α for 15 h to induce expression of the reporter protein prior to addition of CHX. Cells were harvested at various time points after CHX addition and lysates were analyzed by Western blot or flow cytometry. We determined a half-life of only ~2 h for IFIT1deGFP and ~4 h for Mx1deGFP (Fig 2F, left and right panel, respectively). Thus, the cell lines stably expressing the BAC-encoded deGFP fusion proteins are suitable for studying the dynamics of the IFN response.

### Variability of the IFN response at the single cell level

**Fig 3:**
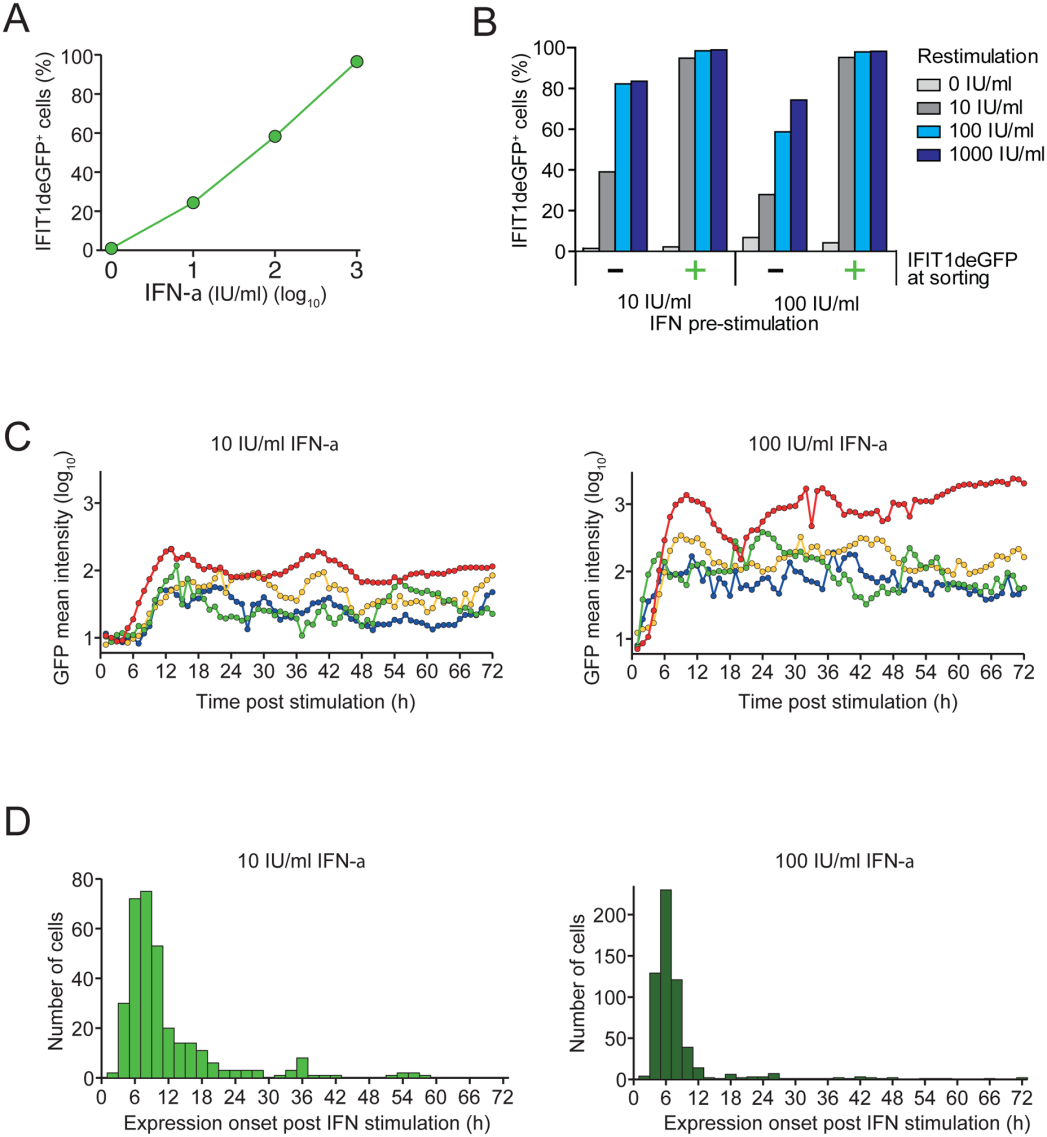
Heterogeneity of the IFN response at the single cell level. (**A**) A549-IFIT1deGFP cells stimulated with increasing amounts of IFN-α for 24 h were analyzed for IFIT1deGFP expression by flow cytometry. (**B**) A549-IFIT1deGFP cells stimulated with 10 or 100 IU/ml IFN-α for 24 h were subjected to FACS. IFIT1deGFP-positive and -negative cells were separated, seeded and re-stimulated 24 h later using IFN-α concentrations specified in the right. Twenty four hours later, cells were analyzed by flow cytometry. (**C**) Heterogeneity of IFN-α response kinetics at the single cell level. A549-IFIT1deGFP cells were treated with 10 or 100 IU/ml IFN-α (left and right panel, respectively) and single cells were tracked by time-lapse microscopy for a total observation period of 72 h. For each time point Z-stacks composed of 16 to 20 consecutive optical sections were acquired and total intensity of IFIT1deGFP in individual cells was extracted. Mean intensities of the IFIT1deGFP reporter per cell are plotted for selected cells (indicated by different colors). A more comprehensive data set is shown in S4 Fig. (**D**) Kinetics of expression onset of IFIT1deGFP in cells treated with 10 or 100 IU/ml IFN-α (left and right panel, respectively). Data are based on live cell imaging results displayed in panel C and in S4 Fig.

We next determined the IFN response at the single cell level by using flow cytometry of the reporter cell lines. As shown in Fig 3A, we observed discrete IFIT1-expressing cell populations upon IFN-α treatment with the fraction of IFN-responding cells increasing continuously with IFN-α dose. However, even at highest IFN-α concentration ~5% of cells did not respond, consistent with stochasticity of the IFN response as described earlier [45,46]. To determine whether IFN-α responsiveness is a stable property of individual cells, we sorted A549-IFIT1deGFP cells treated with either 10 or 100 IU/ml IFN-α into deGFP-expressing and non-expressing fractions. Sorted cells were seeded and treated again with IFN-α (Fig 3B). Nearly all cells that had responded to the first IFN-α treatment responded to the second treatment, possibly as a result of the higher abundance of signal-transduction molecules such as STAT1/2 and IRF-9 [45,49]. However, cells that did not respond to primary IFN-α treatment showed practically the same dose response as naïve cells during primary IFN-α application (Fig 3B). This result indicates that a stochastic process is involved in ISG expression.

Next we investigated the kinetics of ISG induction upon IFN-α treatment in single cells by time-lapse microscopy. For this purpose we utilized A549-IFIT1deGFP cells that stably express a nuclear localized far-red fluorescent protein to allow automatic tracking of individual cells. We started imaging 1 h after addition of 10 or 100 IU/ml IFN-α and, during a 72 h-observation period, determined for each time point total intensity of IFIT1deGFP in individual cells. As shown in Fig 3C, the level of the short-lived IFIT1deGFP reporter fluctuated in the responding cells over time. However, after switching IFIT1deGFP expression on, the majority of cells remained IFIT1deGFP positive (a more comprehensive presentation of larger cell numbers is given in S4 Fig). The time of IFIT1deGFP induction varied widely with nearly all positive cells switching within 24 h after the IFN-α stimulus (Fig 3D).

Taken together, our data show that a fraction of cells does not respond to IFN with IFIT1 expression. Moreover, cells that do respond exhibit a broad range of delays for IFIT1 induction, but once it is switched on, IFIT1 expression is sustained.

### Characterization of the faR reporter DENV

**Fig 4:**
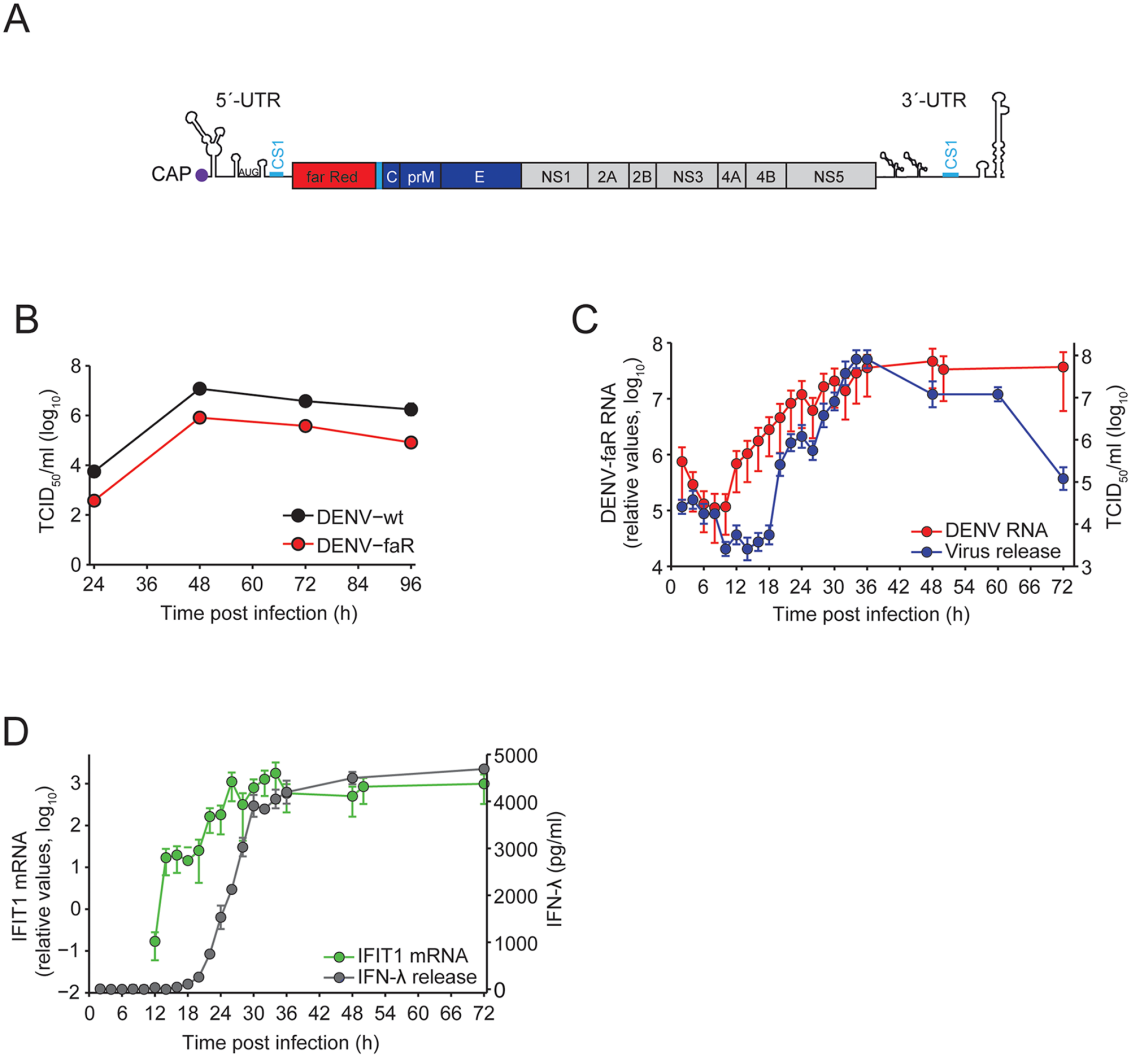
Characterization of the DENV-faR reporter virus. (**A**) Schematic of the DENV reporter virus genome. The first 103 nucleotides of the capsid coding sequence were duplicated, because they contain the circularization sequence 1 (CS1; blue bar) that is essential for DENV RNA replication. Thus, the far red reporter is fused N-terminally with ~34 amino acid residues of the capsid protein that contains a nuclear localization sequence and C-terminally with the sequence encoding for the 2A cleavage factor (PCS) of the Thosea asigna virus, thus generating the authentic N-terminus of the capsid protein. (**B**) A549 cells were infected with the DENV-faR reporter virus or the parental wildtype (wt) lacking a reporter gene at a MOI of 1 TCID_50_/cell. At time points specified in the bottom, culture supernatants were harvested and titers of infectious virus were determined by limiting dilution assay. (**C**) Single-step growth curve of DENV-faR in A549 cells. Cells were infected at a MOI of 10 TCID_50_/cell and harvested at multiple time points. Viral RNA was quantified by RT-qPCR and infectivity released from cells was monitored by limiting dilution assay. Graphs display a representative result out of 3 independent experiments. Values correspond to the mean of triplicate measurements and error bars. (**D**) Kinetics of activation of IFIT1 transcription and IFN-λ secretion. A549 cells were infected with DENV-faR at a MOI of 10 TCID_50_/cell and harvested at time points specified in the bottom of the graph. IFIT1 transcription was determined by RT-qPCR, whereas IFN-λ secretion was quantified by ELISA. IFIT1 mRNA values were normalized to GAPDH mRNA levels.

To study the interplay between the IFN response and the kinetics of DENV replication and spread, we constructed a genetically modified DENV reporter virus genome encoding the far red fluorescent protein TurboFP635 (“faR”) (Fig 4A). This reporter matures rapidly, is pH- and photo-stable and has high signal intensity [50]. The reporter virus, designated DENV-faR, was derived from the DENV-2 isolate 16681 by using the monocistronic construct design we have described earlier [51]. Replication fitness and virus production of this reporter genome was only slightly lower as compared to the parental DENV wildtype (DENV-wt) (Fig 4B). Importantly, reporter virus spread was readily detected in A549 cells. This is best demonstrated by the robust increase of reporter protein-positive cells upon infection with this virus as compared to infection with virus-like particles that support only a single round of infection (S5A and S5B Fig).

To verify that faR protein detection is a reliable marker for DENV-infected cells we infected A549 cells with the reporter virus and determined the kinetics of production of dsRNA, a well-established marker for active virus replication [52] and faR-expression in the same cells (S5C Fig). We found that faR protein expression was only slightly delayed as compared to the appearance of dsRNA, and the presence of dsRNA in a given cell correlated well with faR expression. Thus this reporter virus is a reliable tool to study the dynamics of DENV replication and spread in our A549 ISG reporter cells.

During the setup of the experimental system we found that concentrated DENV-faR virus stocks prepared directly from culture supernatants contained cytokines that induced our reporter ISGs, although the virus was propagated in VeroE6 cells assumed to be IFN-deficient [53]. These cytokines were antiviral as inferred from a HCV-based replication inhibition assay, but could be removed by ultracentrifugation of concentrated DENV-faR stocks through a 20% sucrose cushion (S6 Fig). Using these purified virus stocks we assessed the replication properties of DENV-faR by establishing a single-step growth curve in A549 cells (Fig 4C). RNA replication became first detectable ~8 h after inoculation and infectious virus was released into culture supernatant from ~16 h post infection onwards (S7A and S7B Fig), reaching its half-maximum at ~30 h (note that high amounts of viral RNA and infectivity at early time points are due to residual inoculum sticking to cells even after stringent washing). In agreement with these results, we found an increase of IFIT1 mRNA starting 12 h p.i. and release of IFN-λ from 18 h p.i. onwards reaching its half-maximum at 26 h (Fig 4D). We additionally determined the decay of infectious viral particles in the culture medium and found a half-life of 1.7 h (S7C Fig). We also detected IFN-β in culture supernatants of infected cells (S8 Fig), whereas IFN-α production was not detectable. These results show that DENV-faR replication induces IFN secretion from A549 cells.

### Single cell kinetics of IFN response in DENV-faR-infected A549 reporter cells

**Fig 5:**
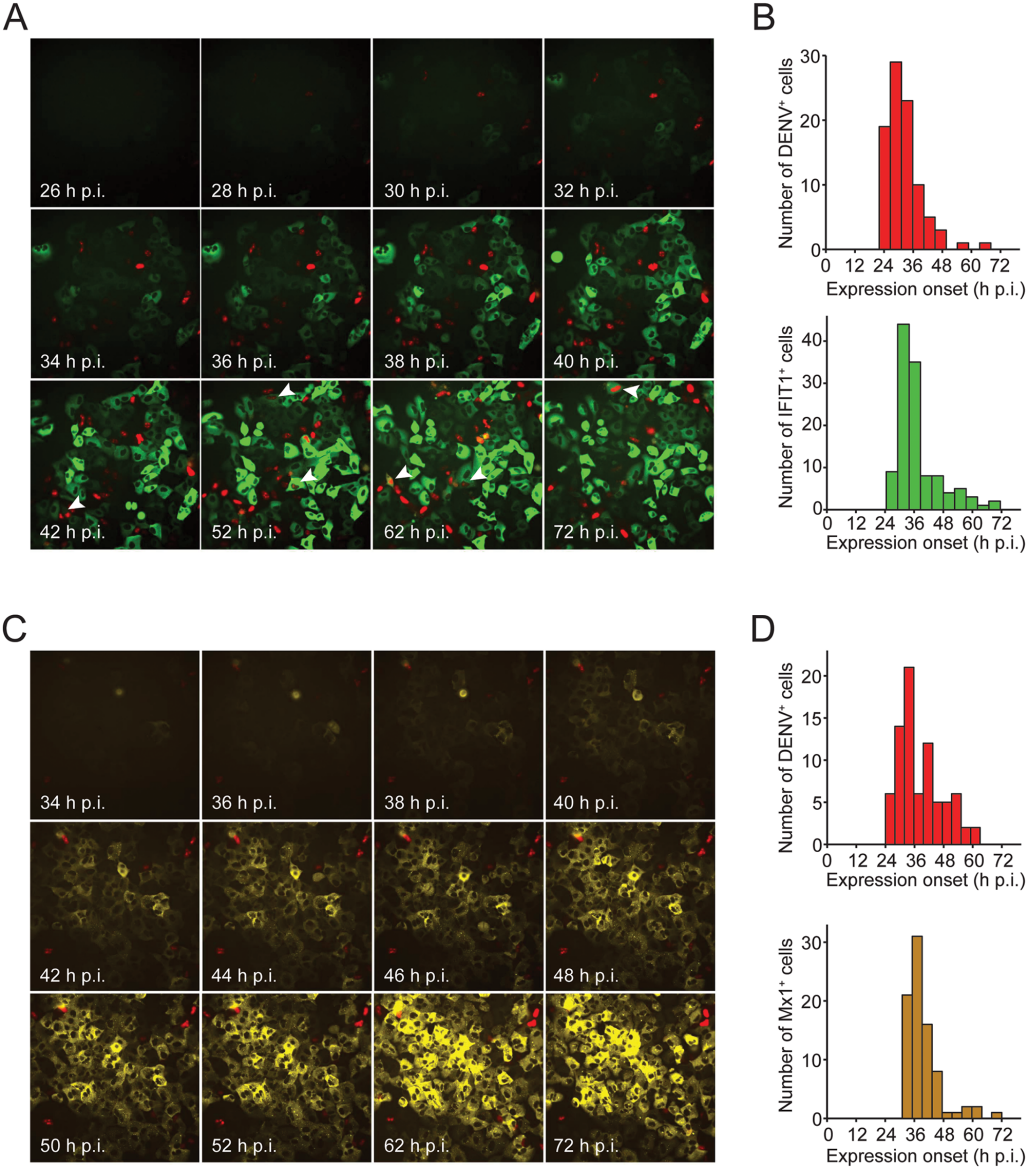
Heterogeneity of IFN induction and DENV-faR replication in A549 reporter cells at the single cell level. A549 reporter cell lines were infected with DENV-faR and time-lapse live cell imaging was started one hour later. Images were recorded in 30 min intervals for IFIT1 and 1 h intervals for Mx1 until 72 h p.i. Images were analyzed by using the ImageJ software package and the MTrackJ plug-in. (**A, B**) A549-IFIT1deGFP cells were infected with DENV-faR at a MOI of 0.2 TCID_50_/cell. (**A**) Representative still images taken at time points specified in the bottom left. Arrowheads point to faR—IFIT1deGFP double positive cells. (**B**) Kinetics of onset of detectable expression of the viral faR reporter gene (upper panel) and the ISG-reporter IFIT1deGFP (lower panel), respectively. (**C, D**) A549-Mx1deGFP cells were infected with DENV-faR at a MOI of 0.1 TCID_50_/cell. Shown are representative still images (**C**) and kinetics of detectable onset of expression (**D**) of the viral faR (upper panel) as well as the Mx1deGFP reporter (lower panel), respectively. In panel B, 91 DENV^+^ and 119 IFIT1^+^ cells were used for the analysis, respectively; in panel D 79 DENV^+^ and 83 Mx1^+^ cells, respectively. The corresponding movies are shown in the supplementary section.

Taking advantage of our reporter cells and virus system, we determined the kinetics of DENV replication/spread and activation of the IFN response by time-lapse microscopy. Experiments were conducted with A549-IFIT1deGFP and A549-Mx1deGFP cells (Fig 5), using low MOI to allow for viral spread through secondary infection events. In both cell lines DENV-faR-expressing cells were first detected 22 h after infection, reflecting the delay from infection to the rise in intracellular viral RNA and expression of the reporter protein to a detectable level (cf. Fig 4C). Given that infected cells start to produce viral particles from 16 h post infection (cf. Fig 4C and S7B Fig) and that infection takes ~20 h to become visible with the faR reporter, the majority of cells becoming DENV-faR-positive between 36 and 72 h p.i. likely reflects viral spread by secondary infection events. Consistent with these kinetics we found that A549-IFIT1deGFP reporter cells infected with the reporter virus at high MOI (10 TCID_50_/cell) displayed a faster onset of faR expression concomitant with a lower number of IFIT1deGFP-positive cells, likely as a result of the JAK-STAT signaling block (S9 Fig).

As deduced from the expression kinetics of our reporters (Fig 5B and 5D), during this time period many cells became protected against viral infection by induction of ISGs. Based on calculations derived from the histograms, the first onset of ISG-reporter expression was delayed relative to the appearance of DENV-faR-positive cells by 4.75 h, reflecting the minimal time that is required to activate the IFN response by DENV. Previous studies suggested that IFIT1 expression can be stimulated directly by the IRF3 signaling cascade [54,55] and thus, should be induced in DENV-infected cells independent of exogenous IFN. However, in only ~10% of DENV-faR positive cells, we found also IFIT1deGFP expression arguing that IFIT1 expression is induced primarily by IFN, at least in our cell culture system (Fig 5A; S1 Movie). Alternatively, the low expression of IFIT1deGFP in DENV-containing cells might be due to viral blockade of the IRF3 pathway. In contrast to IFIT1, the induction of Mx1 requires IFN [48] and indeed, only very rarely we detected Mx1deGFP–DENV-faR double-positive cells (Fig 5C; S2 Movie). Moreover, the fact that DENV-faR-expressing cells did not induce Mx1deGFP is consistent with an efficient block of JAK-STAT signaling in DENV-infected cells.

Overall, the time courses of IFIT1deGFP and Mx1deGFP induction were similar, further supporting that secreted IFNs are the primary inducers of the antiviral response. With only rare exceptions, cells expressing either ISG reporter remained DENV-faR negative, showing that IFN produced from DENV-infected cells protected naïve cells against virus infection. However, at the same time viral spread occurred, showing that the protection of cells through the IFN response is not sufficient to curb DENV spread.

### Quantitative model of viral spread and IFN response

**Fig 6:**
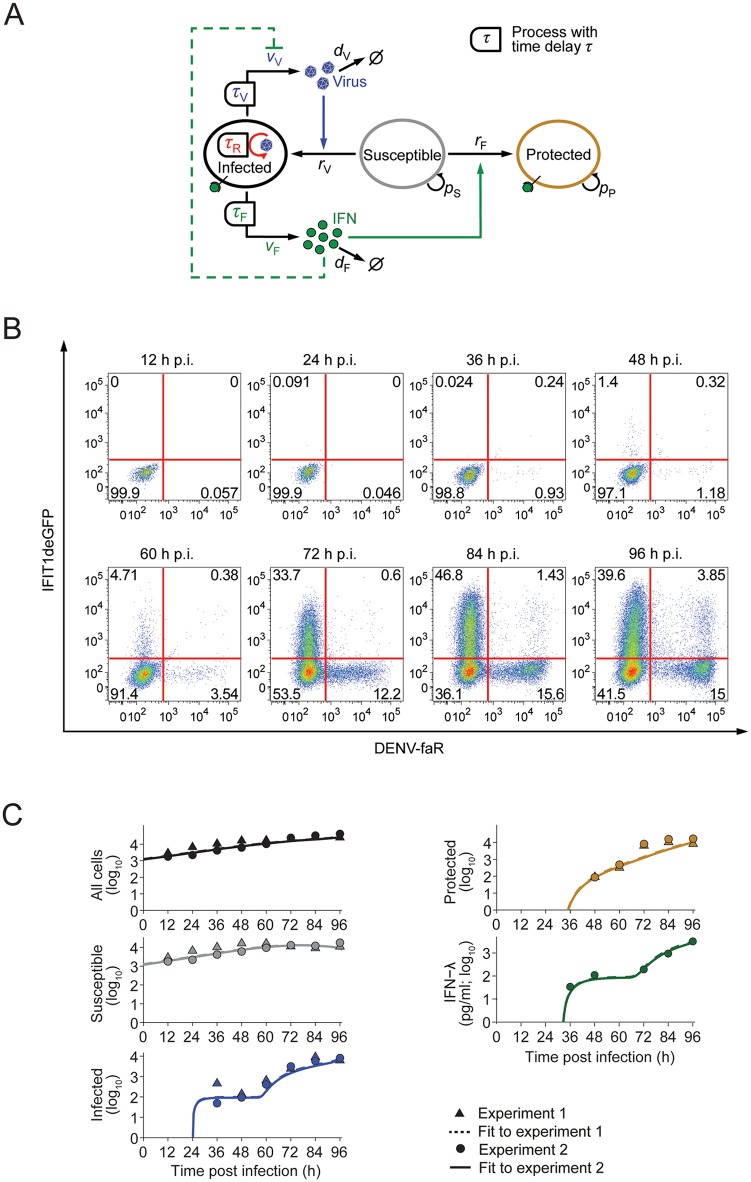
Quantitative model of DENV spread and IFN response. (**A**) Schematic of the mathematical model: Virus replicates in infected cells after time delay *τ*_R_ and produces virus with time delay *τ*_V_ and rate *v*_V_, which can infect susceptible cells (blue arrow). Infected cells produce IFN after time delay *τ*_F_ with rate *v*_F_, which protects susceptible cells (solid green arrow) and can induce antiviral genes in infected cells (dashed green arrow). For further details of the mathematical model see S1 Supplementary Methods. (**B**) Spread of DENV-faR virus and activation of the IFN response. A549-IFIT1deGFP cells were infected with DENV-faR at a MOI of 0.1 TCID_50_/cell. At time points specified in the top of each panel, cells were fixed and processed for measurement by flow cytometry. (**C**) Best fit of the mathematical model to two independent experiments; the initial conditions for the number of susceptible cells are specific to each experiment whereas the model parameters are the same for both experiments. The numbers of susceptible, DENV-faR positive (i.e. infected) and IFIT1deGFP positive (i.e. protected) cells have been taken from the flow-cytometry data (the comparatively small number of double-positive cells was counted as infected); IFN-λ was measured in experiment 2 by ELISA. Non-infected cells served as control.

To understand which parameters control the kinetics of viral spread and the IFN response, we developed a data-based mathematical model. This model describes the rates of naïve cells becoming infected by extracellular DENV or protected by the action of IFNs released from infected cells (Fig 6A). The model takes into account that in infected cells, DENV replicates after a time delay *τ*_R_ (see Fig 4C). After a time delay *τ*_V_, which considers the additional time necessary for viral protein expression and particle assembly, infectious virions are released. Moreover, infected cells secrete IFN after time delay *τ*_F_ that corresponds to the time necessary for viral recognition, downstream signal transduction as well as IFN-gene transcription, mRNA translation and IFN secretion (Fig 4D). Released IFNs protect susceptible cells (i.e. cells that are sensitive to infection) from infection (solid green arrow in Fig 6A). Importantly, IFNs inhibit virus replication in infected cells if acting early, i.e. prior to DENV-induced shut-off of JAK-STAT signaling (dashed green arrow in Fig 6A; cf. Fig 1A and 1B).

The resulting model is an effective model that describes key parameters of the system and abstracts from much molecular detail. This common strategy in modeling virus-host interactions [56,57] allowed us to identify the values of these key parameters from experimental data. In particular, potential IFN effects on infected cells were incorporated into the value of the virus production rate per cell, *v*_V_. The extracellular diffusion coefficients of DENV and IFN are of the order of 10^4^ μm^2^ h^–1^ and 10^5^ μm^2^ h^–1^, respectively; [58,59]; thus, within one hour DENV and IFN diffuse over distances ~100 μm and ~315 μm, respectively. As virus production and IFN secretion evolve over several hours (cf. Fig 4), we can assume spatial mixing of both DENV and IFN over many cell diameters and therefore neglected spatial gradients in the model. Hence, the numbers of susceptible, infected and protected cells and the extracellular concentrations of DENV and IFN are described by a system of delay-differential equations (DDEs) with time delays for virus replication, virus production and IFN secretion (Fig 6A; S1 Supplementary Methods).

We determined the parameters of the model by fitting time courses of the model variables (infected, susceptible and protected cells as well as extracellular IFN concentration) to time-resolved experimental data. To this end, we infected A549-IFIT1deGFP reporter cells with DENV-faR at very low MOI (0.1 TCID_50_/cell), so that initially few cells are infected and viral spread can readily be observed. We performed time-resolved flow cytometry measurements (Fig 6B; S10A Fig for the Mx1deGFP reporter) along with quantification of IFN-λ, the most prominent IFN species in this cell system (S8 Fig). For the purpose of parameter estimation, we found time-resolved flow cytometry more informative than live cell imaging, because the former gave an accurate count of susceptible cells (double negative) in addition to infected (DENV-faR positive) and protected (ISG reporter-positive) cells. Similar to the time-lapse microscopy, we observed a strong rise of infected cells with a time delay (~60 h), which, given the low MOI, should be due to secondary spread of the virus from initially infected cells. Cell death was not prominent during the observation period.

**Fig 7:**
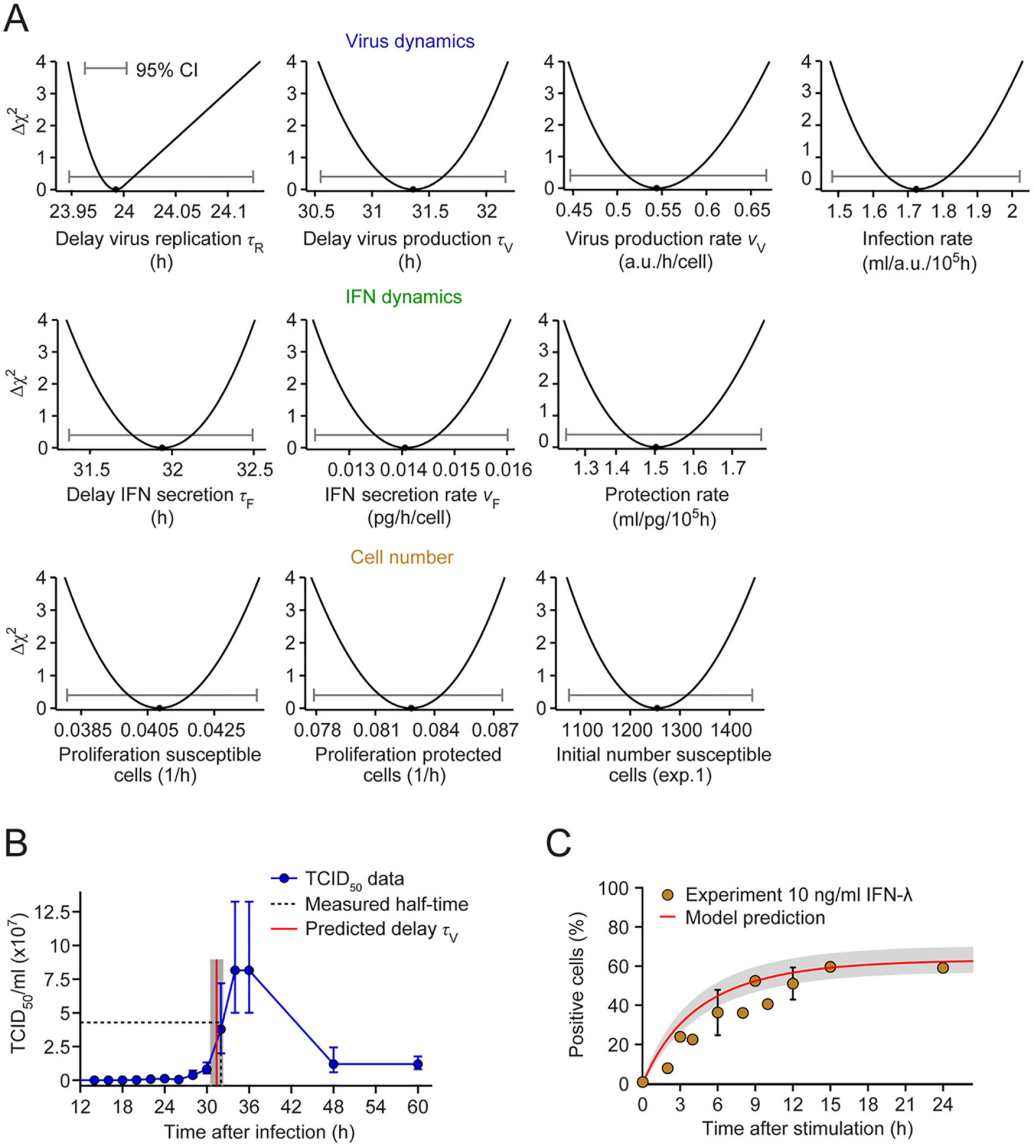
Parameter identification and model validation. (**A**) The profile likelihoods for the model parameters show that all parameters are identified by the experimental data (CI, confidence interval). (**B**) The inferred delay for virus production (red line) matches the half-time for the rise in infectious DENV secreted by infected cells (experimental data replotted from Fig 4C on linear scale). (**C**) The predicted increase in protected cells after IFN treatment (red line with shaded 95% confidence bound) matches the experimentally observed rise in IFIT1deGFP positive cells (yellow dots). The model was simulated with a single application of 10 ng/ml IFN-λ; the corresponding data are replotted from S3B Fig.

We determined an optimal set of model parameters (including the independently measured virus half-life, S7C Fig, and the IFN half-life in the extracellular medium [45]) such that it makes the data (Fig 6B) the most likely outcome of an experimental measurement (maximum-likelihood estimate). Using these parameters, we found that the model fitted the time course data well (Fig 6C). Next, we studied whether the experimental data constrain the model parameters by computing the profile likelihoods for all parameters [60]. The model parameters were fully identified from the data (Fig 7A; S1 Table) and thus, the model is suitable for making quantitative predictions.

To test the validity of the model, we examined three critical parameters using independent experimental data. First, we noted that the delay for intracellular virus replication was estimated from the flow cytometry data to be 24 h (Fig 7A, top row; *τ*_R_), which equals the delay after which DENV became visible in infected cells by using live cell imaging (Fig 5B and 5D). Second, the delay for the production of DENV particles was 31 h (Fig 7A, top row; *τ*_V_), which accurately reflects the independently measured half-time of DENV secretion, i.e. the middle of the log phase (Fig 7B). Thus, the delay for virus production in the model corresponds to this half-time rather than the time of first appearance of viral particles (~16 h). Also note that the estimated time difference of ~8 h between the appearance of replicated viral RNA and secreted infectious DENV is consistent with the estimated delays in the experimental data (Fig 4C and S7A and S7B Fig). The fact that the delays for virus replication and production from the model fit match the experimentally observed times at which infected cells become visibly virus-positive (in live cell imaging) and reach their half-maximal virus production rate (as determined by TCID_50_ assay), indicates that DENV spread requires substantial production of infectious virus particles. As a third test of the model, we considered the simulated response of the cells to an external application of IFN-λ (S3B Fig). Again, the model prediction matches these independent data well (Fig 7C). In summary, the mathematical model agrees with key data obtained in independent experiments that were not used for model fitting.

### Spread of mutant DENV with defective 2’-O-methylation

**Fig 8:**
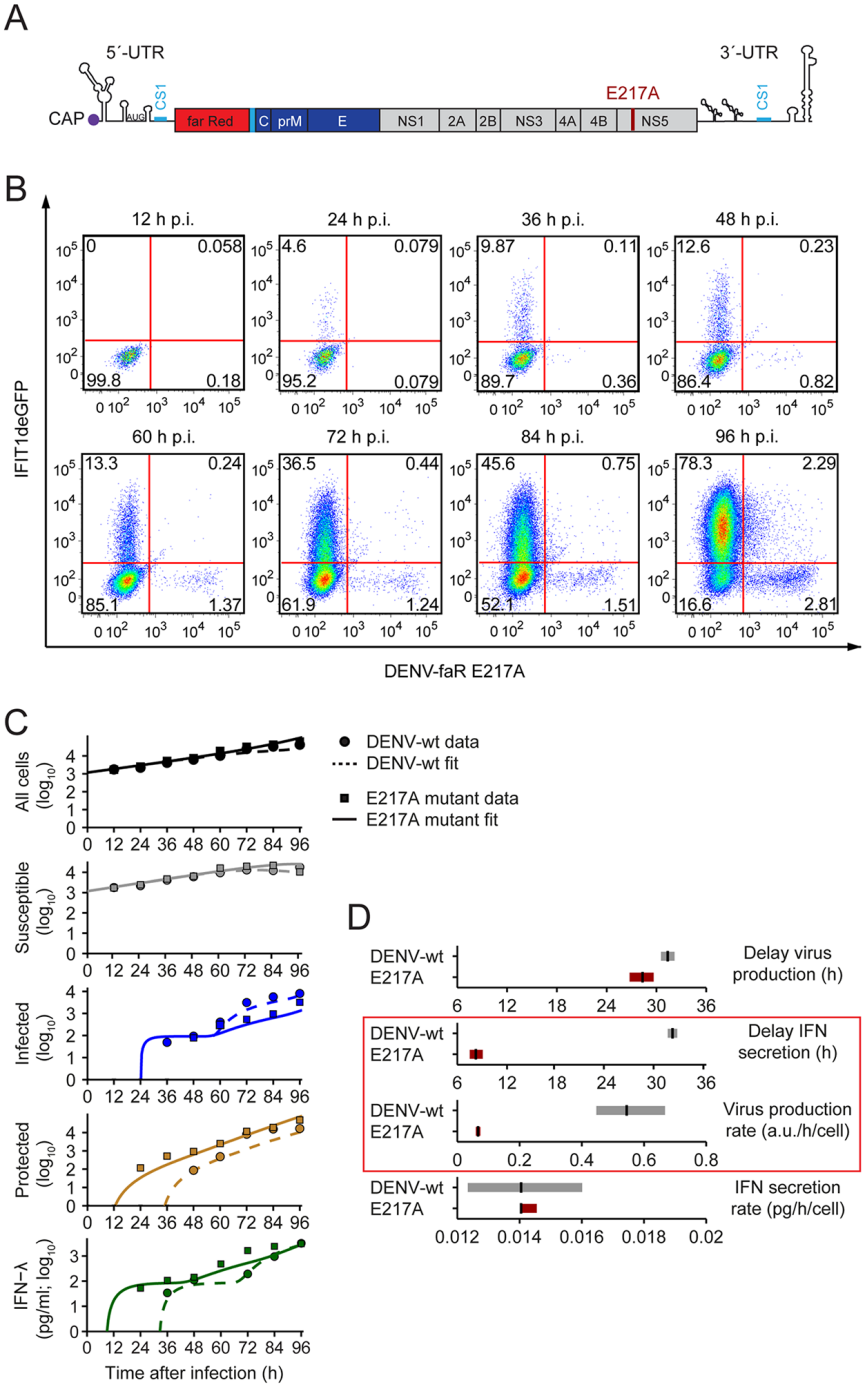
Spread of a DENV mutant with defective 2’-O-methylation of the viral RNA genome. (**A**) Genomic structure of the DENV-faR 2’-O-methylation mutant containing the E217A substitution in the NS5 protein (for further details see legend to Fig 4A). (**B**) Spread of the DENV-faR mutant in A549 reporter cells and activation of the IFN response. A549-IFIT1deGFP cells were infected with the DENV-faR E217A mutant at a MOI of 0.1 TCID_50_/cell. At the time points specified in the top of each panel, cells were fixed and processed for measurement by flow cytometry. (**C**) Model dynamics versus experimental data from (B) (solid lines and squares, respectively); for comparison, the model dynamics and data for wildtype DENV are reproduced from Fig 6C (dashed lines and circles, respectively). IFN-λ was measured by ELISA. Non-infected cells served as control. (**D**) Best-fit values and confidence bounds for the model parameters that have been allowed to differ between wildtype DENV and the 2’-O-methylation mutant. The model predicts that the virus production rate and the delay of IFN secretion decrease in case of the mutant (red box), whereas the delay of virus production and IFN secretion rate remain unchanged.

Next, we asked whether the model could explain the attenuated spread of a proposed vaccine candidate—a DENV mutant that lacks 2'-O-methyltransferase activity [39,40]. To this end, we replaced the glutamic acid residue 217 in NS5 by an alanine residue in our DENV-faR reporter virus (Fig 8A). This modification impairs 2’-O-methylation of the DENV RNA genome.

To quantify the effect of this mutation on virus replication and IFN response, we infected A549-IFIT1deGFP and A549-Mx1deGFP cells with the DENV-faR E217A mutant (MOI of 0.1 TCID_50_/cell) and performed kinetic analyses analogous to the ones conducted with the wildtype virus. By using FACS-based analyses, in both A549-IFIT1deGFP cells (Fig 8B) and A549-Mx1deGFP cells (S10B Fig), we observed a strong reduction of virus spread as compared to the wildtype virus. Moreover, the analogous impairment of spread of this mutant was found when we analyzed the cells by live cell imaging (S11 Fig; S3 and S4 Movies). We reasoned that four key parameters might vary between wildtype DENV and the E217A mutant: As the translation of mutant RNA is impaired because of its sequestration by IFIT1 [41] both the viral production and, possibly, also the delay in virus production after infection could be altered. Moreover, as 2’-O-unmethylated RNA is recognized more readily by Mda5 [29], infected cells might produce IFNs with shorter delay and/or higher rate. To fit the mathematical model to the data obtained with the mutant, we allowed these rates and delays of virus production and IFN secretion to be mutant-specific, but otherwise used the parameters previously established for wildtype DENV. We found agreement of the model with the data (Fig 8C) and, using profile-likelihood analysis, established confidence bounds on the mutant-specific parameters (Fig 8D; S2 Table). Only two out of these four parameters differed strongly from the wildtype value: the virus production rate was decreased 8-fold for the E217A mutant and the delay from infection to IFN production was decreased 4-fold, by ~24 h. Importantly, for the mutant DENV, IFN production sets in prior to virus production whereas the timing of the two events roughly coincides for the wildtype.

### Autocrine IFN action on infected cells is the key determinant for attenuation of the E217A mutant

**Fig 9:**
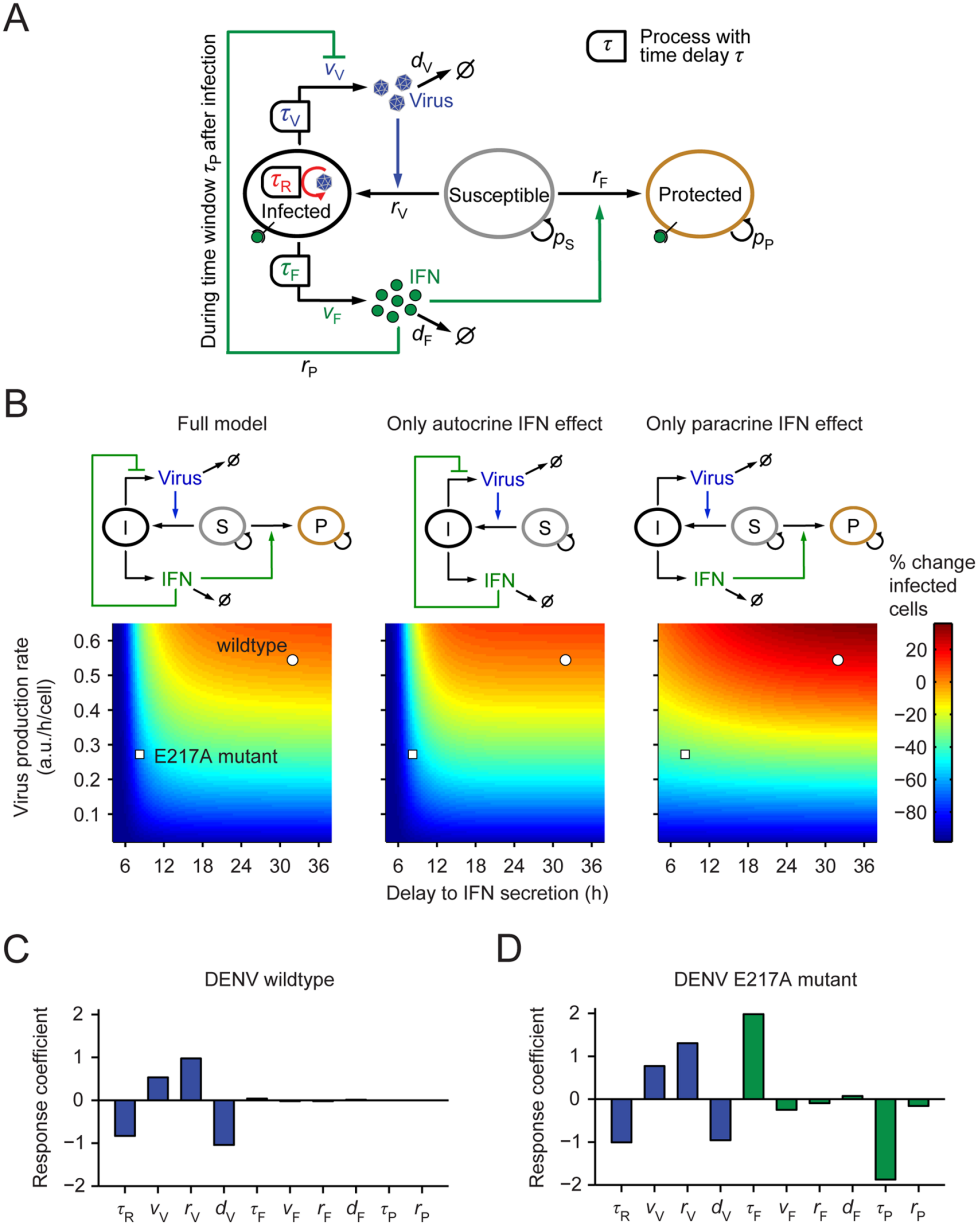
Lack of 2’-O-methylation of the DENV RNA genome causes attenuation of virus spread by IFN action on infected cells. (**A**) Schematic of the extended mathematical model. The inhibition of virus replication by early IFN signals is described explicitly. For further details see legend to Fig 6A. (**B**) Extent of predicted viral spread simulated by the model as a function of the delay from infection to IFN secretion and the virus production rate by infected cells that have not been inhibited by early IFN signals. The color code gives the relative difference from the spread of wildtype DENV in the full model at 96 h p.i. (**C, D**) Sensitivity analysis of the model parameters for infection with (**C**) wildtype DENV and (**D**) the E217A mutant. The response coefficients *R*_*i*_ of model parameter *i* were calculated with the full model. Shown are virus-related (blue bars) and IFN-specific (green bars) parameter effects on infected cells.

Although the model identified the parameters most critical for the attenuation of the E217A mutant, their relative importance was not clear. Specifically, the IFN production delay and the virus production rate might not be independent parameters (as assumed for simplicity in the previous analysis), because virus replication could be affected by IFN in a time-dependent manner through the early induction of ISGs before STAT2 degradation takes effect (see Fig 1). To account for this possibility, we extended the mathematical model (Fig 9A): The inhibition of virus production by IFN was described explicitly and could take place through ISG induction in an initial phase after infection (Fig 9A, solid green inhibition link), assumed to be 8 h. Compared to Fig 1B, this somewhat extended time window accounts for the fact that the initial infection dose in the time-course experiments (Figs 6B and 8B) is much lower, likely delaying the average onset of viral replication. If an infected cell does not perceive IFN within this time period, it becomes a virus producer, albeit with a somewhat reduced production rate (~50% of wildtype DENV), to account for the possibility that basal IFIT1 expression inhibits translation of DENV mutant RNA. With these modifications (S3 Table), the model fitted the kinetics of both wildtype and mutant DENV replication and IFN response as before (Fig 8C), but could now account for ISG induction and the resulting inhibition of viral replication within an early window of opportunity after infection.

We systematically varied the mutant-specific parameters and found that the IFN-independent reduction of the virus production rate attenuates viral spread, but this attenuation was strongly enhanced by the acceleration of IFN secretion (Fig 9B, left panel). When we retained the autocrine IFN effect on virus-infected cells in the model, but cancelled the paracrine IFN signaling branch protecting susceptible cells, the result was virtually unchanged (Fig 9B, middle panel). By contrast, the simulation of only the paracrine IFN effect resulted in much less attenuation of the DENV mutant (Fig 9B, right panel), which is not consistent with the experimental data. Thus, the model predicts that autocrine IFN action on infected cells is critical for the attenuation of the E217A mutant whereas paracrine IFN action has negligible impact.

These computational results indicate that IFN can quench DENV spread by reducing the viral production rate early on in infected cells. While early IFN production by the E217A DENV mutant enables this effect, IFN production in response to the wildtype virus sets in too late for quenching virus production. We then asked how robust these conclusions are, given that the model has been parameterized using a specific experimental setting. Therefore, we systematically varied each model parameter and quantified its effect on the extent of viral spread, using the response coefficients *R*_*i*_ defined as ratio of the fold change of virus-infected cells to the fold change of model parameter *i*. The spread of wildtype virus is controlled only by virus-related processes: production, decay in the medium and infection of susceptible cells (Fig 9C; note that a negative response coefficient implies that viral spread decreases as the respective parameter is increased). The parameters of the IFN response do not have a noticeable effect on viral spread, including faster (paracrine) protection of susceptible cells (*r*_F_). This is in stark contrast to the E217A mutant where the timing of the IFN response (*τ*_F_) and the time window during which IFN can act on infected cells (*τ*_P_) have the dominant control on viral spread (Fig 9D). The dependence of spread of the E127A mutant on the parameters of virus production and infection is very similar to wildtype virus. Together, the results of this sensitivity analysis of the model show that (1) viral spread is consistently controlled by the rates of virus production and infection and (2) a sufficiently early, autocrine IFN response, as elicited by the E217A mutant, curbs DENV spread.

**Fig 10:**
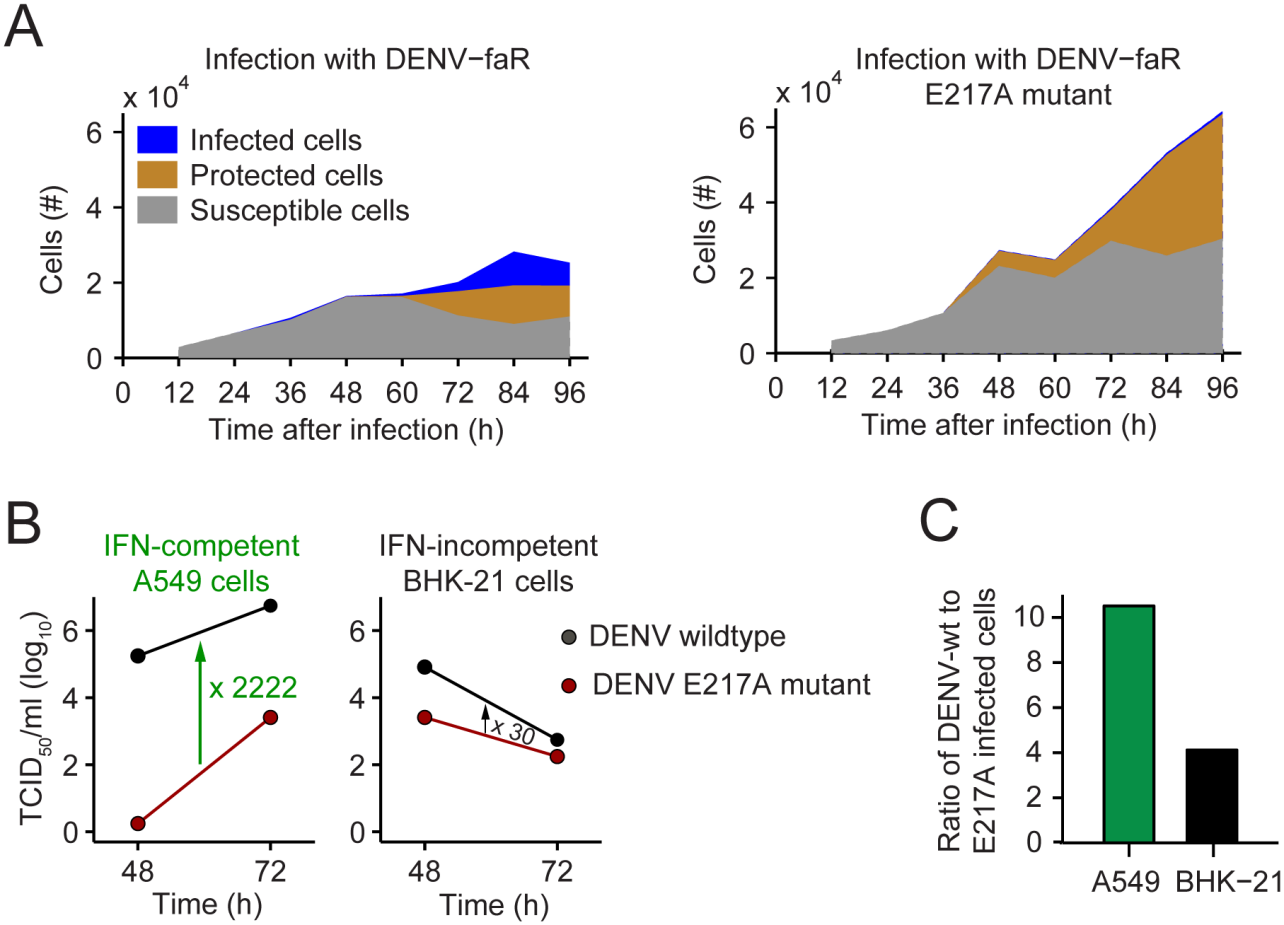
Attenuation of the 2’-O-methylation mutant depends on IFN competence of the infected cell. (**A**) Comparison of the measured time courses of susceptible, infected and protected cells (data from Fig 6B and Fig 8B for DENV-faR and the DENV-faR E217A mutant, respectively), showing large persisting reservoirs of susceptible cells. (**B**) Release of infectious DENV from A549 cells (IFN-competent) and BHK-21 cells (IFN-incompetent). Cells were transfected with DENV RNA genomes and harvested 48 and 72 h later. Titers of infectious virus were determined by limiting-dilution assay. Arrows and numbers refer to fold mean difference of extracellular virus titers. A representative result of three independent experiments is shown. (**C**) Ratio of wildtype- to E217A mutant-infected cells with and without IFN response. A549 cells (IFN-competent) and BHK-21 cells (IFN-incompetent) were infected with DENV-faR wildtype or the DENV-faR E217A mutant at low MOI. The number of faR-positive cells was analyzed by flow cytometry at 72 h p.i. and the ratio between DENV wildtype and E217A mutant-infected cells was calculated.

Consistent with these results, our experimental data revealed a large fraction of susceptible cells that persisted throughout the entire observation period (Fig 10A; overall cell numbers are higher in the case of infection with the E217A mutant, because virus spread is low and hence growth of cells is largely unimpeded). Thus, a large fraction of cells neither becomes infected by the virus nor protected by upregulation of ISGs, implying that the availability of susceptible cells is not a limiting factor for the spread of mutant DENV. This observation is consistent with the live cell imaging results showing that antiviral protection is always incomplete due to high cell-to-cell heterogeneity in the induction of ISGs (Fig 5).

To validate experimentally the enhanced IFN sensitivity of the DENV mutant, we transfected IFN-competent A549 cells and IFN-incompetent BHK-21 cells with RNA of the DENV-faR wildtype or the DENV-faR E217A mutant. By using electroporation of the respective RNA genome, we hit the vast majority of cells, thus allowing determination of predominantly autocrine IFN effects. In agreement with our model prediction, we observed strong attenuation of replication and virus production of the E217A mutant in A549 cells whereas in BHK-21 cells this impairment as measured by the limiting dilution assay was almost 75-fold lower (Fig 10B). Consistent with this result, we found that 72 h post electroporation, the E217A mutant had infected ten times less A549 cells than wildtype virus whereas the respective reduction in BHK-21 cells was only fourfold (Fig 10C). The low-level reduction of mutant virus production in BHK-21 cells might be due to IFN-independent expression of IFIT1 that blocks translation of 2’-O-non-methylated RNA [54].

With the aim to determine whether these properties are unique to the E217A mutant or are linked to impaired replication fitness in general, we compared induction of the IFN response between DENV wildtype, the E217A mutant and a mutant that owing to an amino acid change in NS4B (mutant P23A) was impaired in RNA replication relative to wildtype to a degree that was comparable to the relative reduction observed with the E217A mutant in IFN-incompetent cells [61]. We infected the cells with high MOI to monitor the replication kinetics of the three DENV-faR reporter viruses largely independent from virus spread. We found that kinetics of virus replication and activation of the IFN response were well comparable between the wildtype and the NS4B mutant P23A (S12A and S12B Fig). Consistent with our live cell imaging data, replication of the E217A 2’-O-methyltransferase mutant was very much reduced and only ~10% of cells were faR positive 96 h post infection, in contrast to ~60% in case of wildtype and the NS4B mutant (S12C Fig). Moreover, in spite of this low number of E217A mutant-positive cells, ~90% of the cells mounted an IFN response which was ~20% higher than in cell cultures infected with the wildtype or the NS4B mutant 96 h post infection. Thus, the enhanced IFN sensitivity of the DENV 2’-O-methyltransferase mutant is a unique property that is not linked to replication fitness.

Taken together, our data provide strong evidence that attenuation of the E217A mutant is linked primarily to the magnitude and kinetics of induction of IFN acting on infected cells in an autocrine manner. This IFN response likely is too high and too fast for the virus to establish IFN resistance, e.g. by quantitative STAT2 degradation, thus accounting for the serious impairment of replication fitness of this mutant in an IFN-competent system. It is also for this reason that paracrine IFN action plays a minor, if any, role as virus production rate is reduced so profoundly that virus spread remains limited.

## Discussion

The outcome of virus infections, either rapid control or spreading, very much depends on the kinetics of replication of a given virus and activation of the innate antiviral defense. Although this is a well-established paradigm in virology, most studies are based on measurements under steady-state conditions and only rarely take into account the dynamics of virus replication/spread as well as the kinetics of activation and stochasticity of the IFN response. Several earlier reports demonstrated that induction of ISGs rendered cultured cells resistant against DENV infection [38,42,62], but the kinetics of activation of the antiviral state in relation to virus replication and spread have not been quantified in a single system. To overcome this limitation, in the present study we established BAC-based IFN-competent reporter cell lines that allow monitoring of DENV replication and spread, as well as activation of the IFN response in live cells. We chose two different markers: first, IFIT1 that appears to be activated in an IRF3-dependent manner; second Mx1 that is induced exclusively by IFN, thus serving as selective marker for an intact JAK-STAT signaling pathway [55] (reviewed in [63]). By using live cell microscopy we observed profound heterogeneity of the IFN response at the single cell level arguing, for a stochastic response. This observation is in good agreement with earlier reports describing heterogeneous induction of the *IFNB1* gene upon viral infection [58,64–67] or IFN treatment [46]. Moreover, a recent study identified an “all-or-nothing” expression of IFN-β and ISGs induced by infection of mouse cells with Newcastle disease virus and differing between individual cells [45]. Consistent with our data this study showed that one IFN-secreting cell protects a large number of naïve cells by induction of ISG expression. However, Rand and co-workers utilized an avian virus that did not spread in the murine cell culture and therefore could not identify the contribution of autocrine and paracrine IFN response to curbing the spread of infection. We found that owing to stochasticity the IFN response is “leaky”. Thus, a fraction of non-protected cells remains that supports virus replication. While in the case of DENV these cells will undergo apoptosis as a result of viral replication, in the case of persistent virus infections such as those caused by HCV, this stochasticity might play an important role for chronicity [46]. The molecular determinants underlying the stochastic nature of the IFN response are poorly explored, but likely due to limiting amounts of factors involved in IFN perception, signal transduction or transcription of ISGs.

Mathematical modeling of viral infections has a rich tradition [56,68], but models that include the immune response explicitly have primarily focused on the adaptive immune response [69]. By comparison, the initial phase during which an invading virus must overcome the innate immune response to spread has rarely been addressed [70–73] and experimental data for the parameterization of such models have been scarce. Here we have developed a mathematical model for the dynamic interaction of viral spread and the IFN response and identified all model parameters from a consistent set of experimental data based on DENV infection of IFN-competent human cells. The model specifically studies spreading infections that originate from few initially infected cells, thus using experimental data with low MOI. In high MOI infections, the initial virus load of the infected cells is expected to be more heterogeneous, which might be more accurately described by more complex, agent-based simulation models. The model also abstracts from several details. Notably, the spatial heterogeneity of cell responses observed in our live cell imaging analysis has not been described explicitly, because on the relevant time scale for virus replication and host gene expression, all cells in the culture are likely to be confronted with spatially averaged virus and IFN concentrations [58,59]. This might change in densely crowded tissues where the slowing of both viral spread and IFN diffusion could cause, at least initially, more localized infections and IFN responses [71,74]. A related aspect is the stochasticity of the IFN response that we have characterized both by flow cytometry and live cell imaging. The model accounts for the observation that ISG induction occurs in individual cells at very different times even after uniform IFN treatment. This is naturally reflected in the slow rate of ISG induction (see Fig 7C for a comparison of model and data), which will appear stochastic at the single cell level.

The model based on kinetics of wildtype DENV replication and IFN induction predicted two synergistic effects that account for attenuation of the 2’-O-methylation deficient mutant: first, reduced production of virus particles likely resulting from the antiviral activity of IFIT1; second, accelerated kinetics of IFN activation. Indeed, by using time-resolved flow cytometry we validated these predictions and observed faster cytokine production in the case of the mutant (compare Fig 6B with Fig 8B; see also S10 Fig). Importantly, this mutant has no general defect in RNA replication, because replication and virus production were much less reduced in IFN-incompetent BHK-21 cells as compared to IFN-competent A549 cells. The reduction of the mutant in BHK-21 cells might be due to low basal expression level of IFIT1 that binds RNA lacking 2’-O-methylation and blocks its translation by competing with translation factor EIF4E for mRNA templates [27–33,41]. However, in IFN-competent cells, the 2’-O-unmethylated RNA induces a much faster IFN response. By using our data-based model, we calculated a ~24 h advance in the onset of IFN secretion in the mutant compared to the wildtype (Fig 8D). It is plausible to assume that the delay observed with the mutant is too short to mount an IFN-resistant state in the infected cells, which requires sufficient expression of, amongst others, NS5 and quantitative degradation of STAT2 (reviewed in [7]). As a consequence, cells infected with the mutant are exposed to IFN at a time when they are still IFN responsive, thus causing a profound reduction of virus production rate. In the case of the wildtype, recognition of viral (2’-O-methylated) RNA is inefficient and therefore, the IFN response is induced at a later time point when high amounts of single strand viral genomes or double strand replication intermediates have accumulated. By this time, JAK-STAT signaling would already be shut down and therefore, IFN would not impact viral replication in these already unresponsive infected cells.

Another observation consistent with our model is the higher number of IFIT1deGFP—DENV- or Mx1deGFP—DENV-wildtype double positive cells as compared to the mutant virus (best visible in the flow cytometry results). Given the slower activation kinetics of the IFN response, the time period available to produce progeny wildtype virus without impact of autocrine IFN is much longer and thus, virus spread is much more extended. Therefore, second round infections can occur fast enough before a robust antiviral state has been established in susceptible cells by paracrine IFN action, especially in the subpopulation of naïve cells with slow IFN response. According to our live cell imaging data ~5% of cells respond to IFN even later than 24 h after exposure to the cytokine (Fig 3D). This number is consistent with the fraction of double positive cells we observed by flow cytometry (Fig 6B).

In conclusion, this study describes the dynamic nature of the interaction between DENV and the IFN response in mathematical and experimentally validated terms. Obviously, the next step will be to integrate the intracellular steps of DENV replication, e.g. by using mathematical models analogous to the one we recently established for HCV [75]. Computation of processes in the DENV replication cycle, which are most amenable to antiviral treatment and/or most sensitive to the IFN-induced antiviral state should allow predicting best synergies between antiviral drugs and the (innate) immune response. In this respect, the results described here should foster development of novel antiviral concepts to combat DENV infection and help to understand the mechanisms of attenuation underlying engineered DENV vaccine candidates.

## Materials and Methods

### Cell culture

Cell lines A549, BHK-21, VeroE6, Huh7.5 (kindly provided by C. M. Rice, Rockefeller University, New York, NY) and Huh7/LucUbiNeo-ET [76] were cultivated at 37°C, 5% CO_2_ and 90% relative humidity in Dulbecco’s modified minimal essential medium (Life Technologies, Darmstadt, Germany) supplemented with 2 mmol/L L-glutamine, nonessential amino acids, 100 U/ml penicillin, 100 μg/ml streptomycin and 10% fetal calf serum (DMEMcplt).

### Virus constructs

The pFK-DVs construct derived from isolate 16681 was reported previously [51]. Turbo fluorescent protein 635 (TurboFP635) is a far-red mutant of the red fluorescent protein from the sea anemone *Entacmaea quadricolor* [50]. A mammalian expression vector encoding humanized TurboFP635-C was purchased from Evrogen, Moscow, Russia. The TurboFP635 coding sequence was amplified by PCR and inserted in-frame upstream of the capsid coding region into the pFK-DVs-R2A Renilla luciferase reporter virus construct, thus giving rise to pFK-DVs-faR2A. Plasmid pFK-DVs-faR2A-NS5A-E217A was derived from pFK-DVs-faR by site-directed mutagenesis. pFK-DVs-faR2A-NS4B-P23A was obtained by insertion of a NruI-XbaI fragment that had been isolated from pFK-DVs-R2A NS4B P23A [61] into pFK-DVs-faR2A using the same restriction sites.

### Virus production and purification

BHK-21 cells were transfected with *in vitro* transcribed capped viral RNA. Four hours after transfection, cell culture medium was replaced by fresh DMEMcplt containing 15 mM HEPES [pH 7.5]. Cells were cultured until a cytopathic effect (CPE) became visible. Virus-containing cell culture supernatants were harvested, cleared from cell debris by filtration using a 0.45μm syringe filter tip and frozen at -70°C. For further amplification, DENV contained in this culture supernatant was used to infect VeroE6 cells at a multiplicity of infection (MOI) of 0.1 tissue culture infectious dose 50 (TCID_50_) per cell in DMEMcplt containing 15 mM HEPES [pH 7.5]. Virus-containing cell culture supernatants were harvested on day 4 and 6 post infection. Pooled supernatants were filtered through a 0.45 μM pore-size filter and filtrate was layered onto a cushion composed of 20% (w/v) sucrose dissolved in NTE buffer (120 mM NaCl, 12 mM Tris-HCl [pH 8.0], 2 mM EDTA). After 2h centrifugation at 28.000 rpm (SW32 Ti rotor; Optima LE-80K Ultracentrifuge, Beckman, Krefeld) at 4°C, an aliquot of the supernatant was frozen at -70°C to determine efficiency of purification whereas residual supernatant and sucrose was aspirated. The virus-containing pellet was resuspended in DMEM containing 5% fetal calf serum and 15 mM HEPES [pH 7.5], aliquoted and stored at -70°C until further use. Titers of infectious virus contained in the original culture supernatants and purified stocks were quantified by limiting dilution assay as described elsewhere and infectivity titers were calculated as described earlier [77]. To determine efficiency of removal of antiviral cytokines, we used a bioassay that is based on the inhibition of HCV replication in the reporter cell line Huh7/LucUbiNeo-ET [78]. In brief, LucUbiNeo-ET cells were seeded into 96-well plates and 24 h later incubated for an additional 48 h in the presence of UV-inactivated purified DENV or the original culture supernatant used for purification. Cells were lysed and HCV replication was determined by using luciferase assay as described previously [78,79].

### Infection of cells with DENV

Target cells were seeded one day prior to infection in appropriate cell culture dishes or multi-well plates. After washing the cells once with PBS, virus diluted in DMEMcplt to the desired MOI, given in TCID_50_ per cell, was added for one hour at 37°C and 5% CO_2_ with occasional rocking. After removal of the inoculum, cells were washed thrice with PBS prior to adding an appropriate amount of DMEMcplt.

### Generation of A549-derived cells stably expressing reporter constructs

To obtain A549-derived cells expressing an Mx1- or IFIT1-tagged deGFP in the most authentic genomic context, cells were transfected with a bacterial artificial chromosome (BAC), which allowed expression of the reporter gene under control of all regulatory elements upstream and downstream of the coding sequence. The following BACs were used: human BAC Mx1 RP11-121A5 and human BAC IFIT1 RP11-46M17 (Source BioScience, Cambridge, UK). These BACs encoded for fusion proteins composed of Mx1 or IFIT1, each fused C-terminally to enhanced GFP and the degradation domain of mouse ornithine decarboxylase (mODC). The latter reduced the half-life of the fusion protein, thus allowing studies of the dynamics of the IFN response [80]. The original Mx1- or IFIT1-BACs were modified by using the RED/ET recombination method according to an adapted protocol of the Quick and Easy BAC Modification Kit (Gene Bridges, Heidelberg, Germany). In this way we inserted the GFP tagging cassette (kindly provided by Frank Buchholz, Dresden, Germany) and the mODC degradation domain [80,81]. The latter was introduced into the tagging cassette-containing vector by using the pMARS eGFPmODC plasmid (kindly provided by Andriy Khobta, Mainz, Germany) and standard cloning techniques that can be obtained upon request. A549 cells were transfected with BAC-constructs by using the Effectene transfection reagent as recommended by the manufacturer (Qiagen, Hilden, Germany) and cell clones with stable integration of the BAC as well as strong signal-to-noise ratio after stimulation with IFN were selected as previously described [81].

### Characterization of A549 BAC reporter cells

A549-IFIT1deGFP or A549-Mx1deGFP cells were treated with IFN-α (Sigma-Aldrich, Munich, Germany) or IFN-λ (PeproTech, Hamburg, Germany), harvested at time points specified in the results section, fixed with 2% paraformaldehyde in PBS and analyzed by flow cytometry using a FACSCalibur and the CellQuestPro Software package (Becton Dickinson, Heidelberg, Germany). For fluorescence activated cell sorting, A549 reporter cells were treated with 10 or 100 IU/ml IFN-α for 24 h, harvested by trypsinization followed by centrifugation (700 rpm, 5 min) and resuspended in PBS containing 5% dissociation buffer (Gibco Life Technologies, Darmstadt, Germany). Cell sorting was performed with a FACSAria flow cytometer (Becton Dickinson). GFP-positive and -negative cells were either directly lysed to prepare total RNA for RT-qPCR analysis or seeded at a density of 2 x 10^4^ cells/well of a 24-well culture plate. Twenty four hours after sorting, cells were treated with 10, 100, or 1000 IU IFN-α/ml or left untreated and incubated for an additional 24 h. Cells were then fixed with 2% paraformaldehyde and analyzed by flow cytometry using a FACSCalibur (Becton Dickinson).

### Time-lapse microscopy

Around 10^5^ A549 or A549-reporter cells were seed into a 35mm-diameter glass bottom culture dish (MatTek Corporation, USA) and ~24 h later, cells were infected for 1 h at 37°C with occasional rocking. After removal of the inoculum, cells were washed thrice with PBS and 2ml phenol red-free DMEM containing 5% fetal calf serum was added. For time-lapse microscopy of DENV-infected cells, a Nikon Eclipse Ti inverted microscope system (Nikon, Japan) with motorized stage and climate chamber (PeCon, Erbach, Germany) was used. Ten to 60 observation fields were defined and image acquisition was performed at intervals of 30 or 60 min by using the automated Nikon perfect focus system and GFP as well as TRITC (tetramethylrhodamine) filters (100ms exposure times). Time-lapse microscopy experiments of IFN-α-treated A549-IFITdeGFP cells were also carried out using a Leica TCS SP5 confocal microscope (Leica, Germany) with motorized stage and climate chamber (EMBL, Heidelberg). Ten to 20 observation fields were defined and image acquisition was performed at intervals of 60 min by acquiring 20 Z-stacks for each position to compensate for Z-drift.

### Determination of the half-life of GFP-tagged reporter proteins

A549-Mx1deGFP or -IFIT1deGFP cells were incubated with 100 IU/ml IFN-α for 15 h prior to addition of 100μg/ml cycloheximid for various time points. Cells were either lysed for analysis by Western blot or fixed with 2% paraformaldehyde in PBS for flow cytometry analysis.

### Flow cytometry analyses

For intracellular Mx1 staining, cells were fixed and permeabilized with BD Cytofix/Cytoperm solution as recommended by the manufacturer (BD Biosciences, San Jose, USA). Washing of cells and staining was carried out using 1x BD Perm/Wash buffer (BD Biosciences, San Jose, USA). Mx1 was detected by using the mouse monoclonal MxA-specific antibody M143 (kindly provided by Georg Kochs, Freiburg, Germany). After three washing steps, phycoerythrin-conjugated goat anti-mouse antibody was added (Santa Cruz Biotechnology, Heidelberg, Germany). Cells were washed thrice with 1x BD Perm/Wash buffer and once with PBS. Stained cells were analyzed using a FACSCalibur flow cytometer and the CellQuestPro Software package (Becton Dickinson).

For the 12 h-time-frame virus kinetics experiments cells were fixed with 4% paraformaldehyde in PBS. IFIT1deGFP or Mx1deGFP and faR expression was analyzed by using a MACSQuant Analyzer (Miltenyi Biotech, Bergisch Gladbach, Germany) and the FlowJo v8 Software package.

### Immunofluorescence

A549 cells were seeded onto glass coverslips at a density of 4 x 10^4^ cells per well of a 24-well culture plate. After infection at an MOI of 10 TCID_50_/cell, cells were fixed at time points specified in the results section using 4% paraformaldehyde in PBS and permeabilized with PBS containing 0.5% (v/v) Triton X100. For immunostaining we used either the dsRNA-specific mouse monoclonal antibody J2 (English & Scientific Consulting, Szirák, Hungary; order no. J2-0803) or a NS5-specific rabbit polyclonal antiserum [43] that were diluted in 5% goat serum in PBS. After 1 h incubation at room temperature, cells were washed thrice with PBS and incubated for 45 min with the appropriate Alexa 488- or Alexa 546-conjugated secondary antibody diluted in PBS containing 5% goat serum. Nuclear DNA was detected by using DAPI fluorescent stain (Life Technologies, Darmstadt, Germany). The percentage of DENV-positive cells was determined by calculating the ratio of NS5-positive cells versus total cell number. For each time point, three view fields (10x magnification) were counted using a Leica DFC350 FX microscope (Leica, Wetzlar, Germany). Image analyses were performed using the ImageJ Software Cell Counter Plugin (W. S. Rasband, ImageJ, US National Institutes of Health, Bethesda, MD; http://rsb.info.nih.gov/ij/, 1997–2006).

### RT-qPCR

Total RNA was isolated from cell lysates by using the NucleoSpin RNA II Kit (Macherey Nagel, Düren, Germany) as recommended by the manufacturer and used for reverse transcription with the Multiscribe reverse transcriptase (Applied Biosystems, Darmstadt, Germany). cDNAs were either directly used for real-time PCR or stored at -80°C until further use. The real-time PCR reaction mix contained 2x Green DYE Master mix (PJK, Kleinblittersdorf, Germany) and 5 μl cDNA. The following primers were used: DENV2 (forward 5’-GCC CTT CTG TTC ACA CCA TT-3’; reverse 5’-CCA CAT TTG GGC GTA AGA CT-3’); IFIT1 (forward 5’-GAA GCA GGC AAT CAC AGA AA-3’; reverse 5’-TGA AAC CGA CCA TAG TGG AA-3’); GFP (forward 5’- CGG CGA CGT AAA CGG CCA CAA GT TC-3’; reverse 5’-TGG TGC GCT CCT GGA CGT AGC CTT-3’); Mx1 (forward 5’- AGC CAC TGG ACT GAC GAC TT-3’; reverse 5’- GAG GGC TGA AAA TCC CTT C-3’); IFIT3 (forward 5’-GAA CAT GCT GAC CAA GCA G-3’; reverse 5’-CAG TTG TGT CCA CCC TTC C-3’); IFN-λ1 (forward 5’-CTG TAC AAC AAG TTC AAG GGA-3’; reverse 5’-GGG CCG GCT CCA CTT CAA CA-3’). GAPDH mRNA (primer sequences are described in [82]) was used for normalization of input RNA. Reactions were performed on an ABI PRISM 7000 Sequence Detection System. Amounts of DENV RNA or ISG mRNA were calculated by using the ΔΔCT method described previously [83].

### Immunoblot analyses

For immunoblot analyses, samples were lysed using 2x protein sample buffer (200 mM Tris [pH 8.8], 5 mM EDTA, 0.1% Bromophenolblue, 10% sucrose, 3% SDS, 2% β-mercaptoethanol). Proteins were separated by electrophoresis into a SDS-polyacrylamide gel and transferred onto a nitrocellulose membrane by using a semidry blotting device. After transfer, membranes were rinsed with PBS and incubated overnight in Odyssey blocking buffer (LI-COR Biotechnology, USA) at 4°C. Membranes were incubated with primary antibodies (MxA-specific rabbit polyclonal antiserum (kindly provided by Ilkka Julkunen, Finland); IFIT1-specific rabbit polyclonal antiserum (Abnova Corporation, Germany); GFP-specific mouse monoclonal antibody JL8 (Clontech Laboratories, USA); STAT2-specific rabbit polyclonal antiserum (Santa Cruz, Heidelberg, Germany); DENV E-specific mouse monoclonal antibody (ATCC, USA); beta actin-specific rabbit polyclonal antiserum (Sigma, Germany)), each diluted in blocking buffer. After 2 h incubation, membranes were washed thrice with 1x PBS, supplemented with 0.05% (v/v) Tween-20 (PBS-T) and appropriate LI-COR IRDye 800CW- or IRDye 680RD-coupled secondary antibodies, diluted in blocking buffer, were added for 45 min. After three additional washing steps with PBS-T and one final washing step with PBS, bound antibodies were detected by using the Odyssey Infrared Imaging system (LI-COR Biotechnolgy, USA).

### IFN-λ-specific ELISA

Supernatants of infected cells were cleared from cell debris by filtration through a 0.45μm syringe filter tip, treated with 1% (v/v) Triton X100 to inactivate infectious DENV particles and stored at -70°C. Amounts of IFN-λ were determined by using the VeriKine-DIY Human IFN-λ / IL-28B/29/28A-specific ELISA (Product No. 61840–1; PBL Interferon Source, USA) and the protocol recommended by the manufacturer. The dynamic range of the assay was 62.5–4,000 pg/ml. Measurements were conducted in high binding 96-well ELISA microplates (Greiner Bio-One, Frickenhausen, Germany).

### VeriPlex Human Cytokine 16-Plex ELISA

Supernatants of infected cells were cleared from cell debris by filtration through a 0.45μm syringe filter tip and 1% (v/v) Triton X100 was added to inactivate infectious DENV particles. Samples were stored at -70°C until further use. Cytokines were detected by using the VeriPlex Human Cytokine 16-Plex ELISA (PBL Interferon Source, USA) using the protocol recommended by the manufacturer. Images were acquired using a ChemiDoc MP Imaging system (Biorad, Munich) and analyzed by using the Q-View software package (Quansysbio, USA).

### Image and data analysis

Microscopy images were analyzed by using the ImageJ (W. S. Rasband, ImageJ, US National Institutes of Health, Bethesda, MD; http://rsb.info.nih.gov/ij/, 1997–2006) and MTrackJ software packages (http://www.imagescience.org/meijering/) and further built-in plugins. Statistical analyses were conducted by using the Microsoft Excel 2010 and GraphPad Prism 5 software packages.

### Analysis of live cell image data

For quantification of the deGFP-tagged ISG-reporters, we developed an automated image analysis approach consisting of three main steps. First, segmentation of cell nuclei for which we adapted a previously introduced approach [84] that is based on the gradient information of the image intensities. Second, the mean intensity of the deGFP-reporter was computed for each single cell by averaging the intensity values within a ring around the corresponding cell nucleus. Third, for cell tracking we adapted a recently reported approach [85] that (a) determined one-to-one correspondences based on feature similarity of cells in subsequent frames; (b) determined events by a likelihood measure and merged the respective trajectories to obtain cell lineage trees; (c) processed computed trajectories, e.g. to merge broken trajectories and to discard tracks starting in frames later than the first frame. In this way cells that newly enter the field of view were not considered.

### Mathematical modeling

The mathematical model was formulated as a set of delay-differential equations. For numerical simulations, we used the RADAR5 framework [86]. For parameter estimation and computation of confidence bounds using profile likelihoods we employed the trust-region-reflective least-squares algorithm of Matlab’s optimization toolbox. For further details, see S1 Supplementary Methods.

## Supporting Information

S1 Supplementary Methods Mathematical modeling. Description of the mathematical model developed and applied in this study. The description consists of: (1) data-driven model of viral spread and IFN-induced antiviral defense; (2) parameterization of the model based on wildtype DENV data; (3) detection of DENV mutant-specific parameters by utilizing the knowledge from wildtype fitting; (4) extension of the population-based model to elucidate the paracrine and autocrine effects of IFN.
(PDF)

S1 Fig IFN treatment protects against DENV infection. IFN-competent A549 cells were treated with 100 IU/ml IFN-α prior to (-4 h) or after (2 h, 8 h) or at the time point of infection (0 h) with the DENV2 strain 16681 at a MOI of 0.1 TCID_50_/cell. Cells were fixed 28 h post infection and analyzed by immunofluorescence using a NS5-specific antiserum. Shown is a representative experiment (n = 2). Mock-treated and DENV-infected cells without IFN treatment served as reference. (**A**) Quantification of infection efficiency. For each time point, 250–350 cells detected in 3 view fields were analyzed for DENV infection. (**B**) Titers of infectious supernatants harvested from the cells shown in panel (A) were determined by limiting-dilution assay.
(TIF)

S2 Fig Elevated ISG mRNA levels in GFP-positive sorted A549-Mx1deGFP BAC reporter cells. A549-Mx1deGFP cells were stimulated with 10 IU/ml IFN-α for 24 h and sorted according to deGFP expression by using flow cytometry. Directly after sorting, GFP-positive and -negative cells were lysed and total RNA was extracted. Amounts of the mRNAs specified in the bottom of the graph were quantified by RT-qPCR and normalized to GAPDH mRNA levels. Data are the mean from two independent experiments and their respective SDs.
(TIF)

S3 Fig Kinetics of IFN-λ-mediated reporter gene activation in stably BAC-transfected A549 cells. (**A**) A549-IFIT1deGFP cells were stimulated with 10 ng/ml IFN-λ. Cells were harvested at time points specified in the top (hours) and lysates were analyzed by Western blot using mono-specific antisera (GFP, IFIT1 and β-actin, top to bottom, respectively). A representative immunoblot of 3 independent experiments is shown. (**B**) Induction kinetics of IFIT1deGFP and Mx1deGFP after treatment of A549 reporter cells with 10 ng/ml IFN-λ. Cells were fixed at time points specified in the bottom and number of GFP-positive cells was determined by flow cytometry. Shown are the mean and SD of 2 independent experiments. (**C**) Dose response assay for IFN-λ. Cells were treated with various concentrations of IFN-λ that are specified in the bottom and 24 h later mean GFP intensity was determined by flow cytometry (left panel; grey line indicates detection limit). The number of GFP-expressing cells (right panel) was determined in the analogous way. Data are means from 3 independent experiments and their respective SDs.
(TIF)

S4 Fig Heterogeneity of IFIT1 expression at the single cell level after IFN-α treatment. A549-IFIT1deGFP reporter cells were treated with 10 IU/ml (**A**) or 100 IU/ml (**B**) IFN-α and monitored by time-lapse microscopy for 72 h. Mean intensity of the IFIT1deGFP reporter was quantified in single cells by automated image analysis as described in the materials and methods section.
(TIF)

S5 Fig FaR is a reliable marker for DENV replication and spread. (**A**) Schematic of the DENV-faR trans-complemented particle (TCP) system. (1) Infectious DENV-faR_TCP_ was produced by transfecting cells that stably express capsid protein—prM and E (for reasons of biosafety two independent expression constructs had to be used) with a subgenomic DENV-faR reporter replicon RNA. This replicon contains the faR reporter gene and lacks C, prM and E that are provided in trans in the engineered helper cell line. DENV-faR_TCP_s (dark red circles) released into the cell culture supernatant were harvested 24 h after transfection and used to infect naïve cells. Infected cells can be detected via monitoring faR expression. (2) DENV-faR_TCP_ is unable to spread in cells that do not express the structural proteins. These cells support only infection and replication, but not virus particle production and virus spread, hence the name “single round infection”. (**B**) Comparison of DENV spread in naïve A549 cells upon infection with the DENV-faR reporter virus or DENV-faR_TCP_s. Cells were infected at a MOI of 0.1 TCID_50_/cell and the fraction of faR-positive cells was detected by flow cytometry at time points specified in the bottom. (**C**) Kinetics of DENV-faR reporter virus replication and spread in A549 cells. Upon infection with DENV-faR at a MOI of 10 TCID_50_/cell, the number of DENV-faR- and DENV-dsRNA-positive cells detectable at given time points was determined by immunofluorescence assay using faR- and dsRNA-specific antibodies. Data are means from 2 independent experiments and their respective SDs.
(TIF)

S6 Fig Removal of antiviral cytokines from DENV preparations used in this study. Absence of antiviral cytokines in DENV-faR virus stocks produced on VeroE6 cells after purification by sucrose gradient centrifugation. Aliquots of unpurified culture supernatants of DENV-infected VeroE6 cells as well as virus pellets and supernatants after ultracentrifugation were added to HCV replicon-containing cells. These replicons are highly sensitive to IFNs and thus, suitable to measure even low amounts of antiviral cytokines [78]. To avoid DENV-mediated lysis of HCV replicon-containing cells, samples were subjected to UV inactivation prior to inoculation of the cells. HCV RNA replication was determined by luciferase assay.
(TIF)

S7 FigIdentification of DENV related parameter values.To determine the initiation of viral RNA replication and virus particle release, we fitted the measured kinetics of (**A**) DENV-faR RNA and (**B**) infectious virus concentration after high dose infection (data replotted from Fig 4C) in log_10_ space using the following objective function *f*_*i*_ with *i* ∈{RNA, Virus}: $\;f_{i}\left(t\right)=\;f_{i,0}\;e^{-\;d_{i}t}+H\left(t-t_{i\text{,on}}\right)\;f_{i\text{,max}}\;\left(1-e^{-\;r_{i}\left(t-t_{i\text{,on}}\right)}\right)$. This function accounts for an exponential decay of the initial amount *f*_*i*,*o*_ with a rate constant *d*_*i*_ at time *t*. The replication and virus production setting in at time *t*_*i*,*on*_ after infection are described with a Heaviside step function *H* multiplied by a first order kinetic that increases with rate *r*_*i*_ and saturates at *f*_*i*,*max*_. The shown best fit is obtained by applying a trust-region-reflective least-squares algorithm with at least 2 × 10^3^ different random initial values. In the case of fitting the TCID_50_ data, we only considered data points measured in the time range from 2 to 36 h p.i. In addition, we calculated the 95% confidence intervals of the parameter values using a non-parametric bootstrap method with sample size 5 × 10^4^ leading to the following results (given in best fit [lower and upper bound of 95% confidence interval]): (A) *f*_RNA,0_ = 10^6.3^[10^5.5^;10^8.9^] relative values, *d*_RNA_ = 0.04[0.01;0.1] 1/h, *t*_RNA,on_ = 7.5[5.5;9.0] h, *f*_RNA,max_ = 10^7.3^[10^5.3^;10^7.8^] relative values, *r*_RNA_ = 0.05[0.04;0.1] 1/h. (B) *f*_Virus,0_ = 10^5.0^[10^4.5^;10^6.4^] TCID_50_/ml, *d*_Virus_ = 0.02[0.01;0.05] 1/h, *t*_Virus,on_ = 16.3[12.3;17.7] h, *f*_Virus,max_ = 10^8.6^[10^4.4^;107^6.3^] TCID_50_/ml and *r*_Virus_ = 0.05[0.004;0.21] 1/h. **(C)** Estimation of the virus degradation rate. The parameter determination of the rate constant of virus degradation *d*_V_ is based on a virus stability experiment in which BHK-21 cells were incubated with DENV-faR virus particles and virus titers (black dots) were quantified by TCID_50_ assay at the indicated time points post incubation. Data were fitted with an exponential decay equation (blue curve) by applying a trust-region-reflective least-squares algorithm with 10^4^ different random initial values. The best fit resulted in *d*_V_ = 0.4/h together with an initial virus concentration of 1.6 × 10^6^ TCID_50_/ml.(TIF)

S8 Fig Induction kinetics of selected cytokines released from A549 cells upon DENV infection. A549 cells were infected with wildtype DENV-faR or the DENV-faR E217A mutant at MOIs of 0.1 (**A, B**) or 5 (**C, D**) TCID_50_/cell, respectively. Supernatants were harvested in 12 h time intervals up to 96 h. Cytokine release was analyzed by using the VeriPlex Human Cytokine Multiplex ELISA kit (16-plex). Note that at MOI 0.1, IFN-λ levels induced by the mutant were higher than that of the wildtype. At MOI 5, IFN-λ levels induced by the mutant exceeded the wildtype-induced levels for early time points (≤ 60 h), but this was reversed at 72 h and 84 h whereas IFN-α measured in parallel was not detected. At both low and high MOI, the spread of the mutant virus was very much diminished. IL, interleukin; n.d., not detectable.
(TIF)

S9 Fig Heterogeneity of IFN induction and DENV-faR replication in A549 reporter cells at the single cell level. A549 reporter cell lines were infected with DENV-faR at a MOI of 10 TCID_50_/cell and time-lapse live cell imaging was started one hour later. Images were recorded in 1 h intervals until 72 h p.i. Images were analyzed by using the ImageJ software package and the MTrackJ plug-in. (**A**) Representative still images taken at time points specified in the bottom left. (**B**) Kinetics of onset of detectable expression of the viral faR reporter gene (upper panel) and the ISG-reporter IFIT1deGFP (lower panel), respectively. In panel B, 161 DENV+ and 51 IFIT1+ cells were used for the analysis, respectively.
(TIF)

S10 Fig Enhanced induction of IFN response and limited spread of the DENV E217A mutant in Mx1deGFP reporter cells. A549-Mx1deGFP reporter cells were infected with DENV-faR (**A**) or the DENV-faR E217A mutant (**B**) at a MOI of 0.1 TCID_50_/cell, respectively. At given time points post infection (p.i.), cells were fixed and 100 μl of the cell suspension was analyzed by flow cytometry. The dot plots illustrate the jointly measured DENV-faR (x-axis) and Mx1deGFP (y-axis) fluorescence intensities of individual cells. Shown is one of two independent experiments.
(TIF)

S11 Fig Attenuation of spread of the 2’-O-methylation mutant in A549 cells as determined by live cell imaging. A549 cells were infected with DENV-faR wildtype (**A**) or the E217A mutant (**B**) at a MOI of 0.2 TCID_50_/cell and monitored by time-lapse microscopy for 72 h. Images were recorded in 30 min intervals. Representative still images taken at time points specified in the bottom left are shown. Images correspond to S3 and S4 Movies, respectively. Shown is an overlay of brightfield and fluorescence images.
(TIF)

S12 Fig Comparative analysis of viral replication and activation of the IFN response by DENV wildtype, the replication impaired mutant P23A and the 2’-O-methyltransferase mutant E217A. A549-IFITdeGFP cells were infected at a MOI of 5 TCID_50_/cell with (**A**) DENV-faR wildtype, (**B**) the P23A NS4B mutant [61] or (**C**) the E217A NS5 mutant, respectively. Cells were harvested 12, 24, 36, 48, 60, 72, 80 and 96 h post infection, fixed and analyzed by flow cytometry. A representative experiment is shown (n = 2).
(TIF)

S1 Table Model parameter estimates based on wildtype DENV data. According to the biological meaning (cf. Fig 6A and S1 Supplementary Methods, section 1), the model parameters were estimated by considering only data regarding wildtype DENV infection. Given are the best fit values and the 95% confidence intervals (calculated with the profile-likelihood method) after fitting the model to the time-resolved data set shown in Fig 6C using a fixed initial viral load along with separately determined values for virus and IFN degradation rates. Abbreviations: arbitrary units (a.u.), confidence interval (CI), experiment (exp.), hours (h), milliliter (ml), picogram (pg).
(DOCX)

S2 Table Model parameter estimates based on E217A mutant data. Using the established wildtype DENV-related parameter values (cf. S1 Table), data concerning E217A mutant infection shown in Fig 8C were fitted by allowing only four parameters to differ between wildtype DENV and the E217A mutant. Given are the best fit values and the 95% confidence intervals (calculated with the profile-likelihood method). Abbreviations: arbitrary units (a.u.), confidence interval (CI), E217A mutant (mut), hours (h), picogram (pg).
(DOCX)

S3 Table Additional model parameters of the full model. The full version of the model (cf. S1 Supplementary Methods, section 4; Figs 9 and 10) was simulated with the given parameter values and for the remaining parameters we used the estimates listed in S1 and S2 Tables. Abbreviations: arbitrary units (a.u.), E217A mutant (mut), hours (h), milliliter (ml), picogram (pg).
(DOCX)

S1 Movie Time-lapse microscopy of A549-IFIT1deGFP reporter cells infected with the DENV-faR reporter virus. A549-IFIT1deGFP cells were infected with DENV-faR at a MOI of 0.2 TCID_50_/cell and time-lapse microscopy was started one hour later. Images were recorded in 30 min intervals until 72 h p.i. Overlay of 488 nm (GFP) and 561 nm (faR) fluorescence channels is shown.
(AVI)

S2 Movie Time-lapse microscopy of A549-Mx1deGFP reporter cells infected with the DENV-faR reporter virus. A549-Mx1deGFP cells were infected with DENV-faR at a MOI of 0.1 TCID_50_/cell and time-lapse microscopy was started one hour later. Images were recorded in 1 h intervals until 72 h p.i. Overlay of 488 nm (GFP) and 561 nm (faR) fluorescence channels is shown.
(AVI)

S3 Movie Time-lapse microscopy of DENV-faR wildtype virus replication in naïve A549 cells. A549 cells were infected with DENV-faR wildtype at a MOI of 0.2 TCID_50_/cell and time-lapse microscopy was started one hour later. Images were recorded in 30 min intervals until 72 h p.i. Overlay of brightfield and 561 nm (faR) fluorescence channels is shown.
(AVI)

S4 Movie Time-lapse microscopy of DENV-faR E217A mutant replication in naïve A549 cells. A549 cells were infected with the DENV-faR-E217A mutant at a MOI of 0.2 TCID_50_/cell and time-lapse microscopy was started one hour later. Images were recorded in 30 min intervals until 72 h p.i. Overlay of brightfield and 561 nm (faR) fluorescence channels is shown.
(AVI)
