# Supplementary material for: Live Cell Analysis and Mathematical Modeling Identify Determinants of Attenuation of Dengue Virus 2’-O-Methylation Mutant
Source: PLoS Pathog. 2015 Dec 31;11(12):e1005345. doi: 10.1371/journal.ppat.1005345 (PMC4697809; doi:10.1371/journal.ppat.1005345)
Supplement: S1 Supplementary Methods — Description of the mathematical model developed and applied in this study. The description consists of: (1) data-driven model of viral spread and IFN-induced antiviral defense; (2) parameterization of the model based on wildtype DENV data; (3) detection of DENV mutant-specific parameters by utilizing the knowledge from wildtype fitting; (4) extension of the population-based model to elucidate the paracrine and autocrine effects of IFN. (PDF) [file ppat.1005345.s001.pdf]

S1 Text - Supplementary Methods

Mathematical modeling

1 Data-driven model of viral spread and IFN-induced antiviral defense

Our studies of the interactions between DENV and the IFN system revealed that efficient viral spread and IFN-induced antiviral immune defense occur simultaneously in an infected cell population (cf. **Fig 1A, B** and **Fig 5**). To analyze the dynamics of the IFN response after infection with DENV at a quantitative level, we established a data-driven mathematical model.

The time-resolved data after wildtype DENV infection (cf. **Fig 6B, C**) suggest that the timing of viral spread as well as IFN release play an important role in the competition between virus and the IFN system. Regarding the high diffusion coefficients of DENV ($2.6\times10^4 \mu m^2/h$, [S1]) and IFN ($1.1\times10^5 \mu m^2/h$, [S2]), spread occurs over many cell diameters on the relevant time scale of hours and spatial gradients of DENV as well as IFN are negligible. Therefore, we developed a population-based delay-differential equation (DDE) model with uniform distribution of secreted IFN and extracellular virus.

The observed single-step growth curves of virus replication, virus production and IFN secretion after high dose infection with DENV (cf. **Fig 4C, D**) illustrate that these processes are heterogeneously distributed among single cells. As an approximation of the temporal distribution of these processes, we take into account the delay of virus replication, virus production and IFN secretion in form of constant time delays.

In our population-based model (**Fig 6A**) we initially consider a number of naïve cells S_0 which are susceptible to virus infection, as well as an initial extracellular viral load V_0. Susceptible cells S can become infected by extracellular virus V with infection rate r_V (cf. blue arrow in **Fig 6A**). Depending on the time elapsed since viruses have entered the host cells, infected cells I can acquire up to three different features in parallel:

- After a time delay τ_R, infected but not yet virus replicating cells $I_{\overline{R}}$ turn into virus replicating cells I_R. The time delay of virus replication τ_R is related to the mean expression onset of virus replication in individual cells monitored after infection with DENV-faR (cf. **Fig 5 and Fig 6B**).
- After a time delay τ_V, infected but not yet virus releasing cells $I_{\overline{V}}$ become virus producing cells I_V and release new generated infectious virus particles with the virus production rate v_V. The time delay of virus production τ_V represents the average time required to replicate, translate viral proteins, assemble and release new virus particles (cf. **Fig 4C**).

– After a time delay τ_F, infected but not yet IFN expressing cells $I_{\overline{F}}$ turn into IFN secreting cells I_F and produce IFN F with the IFN secretion rate v_F. The time delay of IFN secretion τ_F corresponds to the mean onset of IFN expression. It includes the necessary time of viral recognition, downstream signal transduction and IFN transcription, translation and secretion (cf. **Fig 4D**).

Since the time delay τ_V also comprises the duration of virus replication, the condition $\tau_V > \tau_R$ must be fulfilled, while we do not require any temporal link between τ_F and both other time delays. Whether the production of new virus particles takes longer than IFN secretion or vice versa will be determined by the parameter estimation.

According to our experimental study, IFN has two antiviral effects (cf. **Fig 1A, B** and **S1 Fig**):

– Pre-stimulation with IFN demonstrated that secreted IFN can protect naïve cells against DENV infection. To integrate this paracrine IFN response in our model, we take into account an IFN-induced switch from susceptible cells to protected cells P with the protection rate r_F (solid green arrow in **Fig 6A**).

– Stimulation of infected cells with IFN in an early phase after viral entry can activate antiviral defense mechanisms, which reduce virus replication and consequently virus production. To keep the model simple, so that its parameters can be identified from experimental data, we neglect a direct autocrine impact of IFN in the first instance (dashed green arrow in **Fig 6A**). After the model parameterization, we will examine the effect of secreted IFN on infected cells directly with an extended version of our model, which is described in detail in section 4.

In order to include the removal of extracellular virus and IFN by cellular uptake and, to a lesser extent, by extracellular degradation, we assume a decrease of virus and IFN with the rate constant of virus degradation d_V and the rate constant of IFN degradation d_F, respectively.

Moreover, we observed that infected cells proliferate less and die faster than non-infected cells. To compensate this propagation difference and, additionally, to keep the number of model parameters low, we consider an inhibited proliferation in infected cells, whereas susceptible and protected cells proliferate with the rates p_S and p_P.

To establish the DDE system of our model, particular attention must be paid to the formulation of the three features of the infected cells comprising virus replication, virus production and IFN expression, which begin at time τ_R, τ_V and τ_F, respectively. As the time delays τ_m for $m \in \{R, V, F\}$ are free parameters and only bound to the condition $\tau_V > \tau_R$, the properties of the infected cells can arise at different times, overlap each other and take place in parallel. Therefore we consider each feature of the infected cells separately and calculate the number of inactive infected cells $I_{\overline{m}}(t)$ and active infected cells $I_m(t)$ for $m \in \{R, V, F\}$ depending on time $t \in \mathbb{R}$. For this purpose we first require the total number of infected cells I at time t

$$I(t) = I_{\overline{m}}(t) + I_m(t) \quad \forall m \in \{R, V, F\} \tag{1.1}$$

and the corresponding differential equation

$$\dot{I}(t) \overset{\text{Fig 6A}}{=} r_{\mathsf{V}} V(t) S(t). \tag{1.2}$$

The number of inactive infected cells $I_{\overline{m}}$ at time t consists of all cells which become infected in the time period $[t - \tau_m, t]$, since after τ_m inactive cells $I_{\overline{m}}$ progress to active cells I_m. Thus, $I_{\overline{m}}$ is given by

$$I_{\overline{m}}(t) \overset{(1.2)}{=} \int_{t-\tau_m}^{t} r_{\mathsf{V}} V(s) S(s) ds. \tag{1.3}$$

To obtain the differential equation $\dot{I}_{\overline{m}}$, we differentiate both sides of (1.3) with respect to time t and apply the fundamental theorem of calculus:

$$\begin{aligned} \dot{I}_{\overline{m}}(t) &= \frac{d}{dt} \int_{t-\tau_m}^{t} r_{\mathsf{V}} V(s) S(s) ds \\ &= r_{\mathsf{V}} V(t) S(t) - r_{\mathsf{V}} V(t - \tau_m) S(t - \tau_m). \end{aligned} \tag{1.4}$$

Using the preceding equations, the differential equation $\dot{I}_m$ results from

$$\begin{aligned} \dot{I}_m(t) &\overset{(1.1)}{=} \frac{d}{dt} \left(I(t) - I_{\overline{m}}(t) \right) \\ &= \dot{I}(t) - \dot{I}_{\overline{m}}(t) \\ &\overset{(1.2),(1.4)}{=} r_{\mathsf{V}} V(t - \tau_m) S(t - \tau_m). \end{aligned} \tag{1.5}$$

Taking all considerations together, our model is described by the following DDE system:

$$\begin{aligned} \dot{S}(t) &= -r_{\mathsf{V}} V(t) S(t) - r_{\mathsf{F}} F(t) S(t) + p_{\mathsf{S}} S(t) \\ \dot{I}_{\overline{\mathsf{R}}}(t) &= r_{\mathsf{V}} V(t) S(t) - r_{\mathsf{V}} V(t - \tau_{\mathsf{R}}) S(t - \tau_{\mathsf{R}}) \\ \dot{I}_{\mathsf{R}}(t) &= r_{\mathsf{V}} V(t - \tau_{\mathsf{R}}) S(t - \tau_{\mathsf{R}}) \\ \dot{I}_{\overline{\mathsf{V}}}(t) &= r_{\mathsf{V}} V(t) S(t) - r_{\mathsf{V}} V(t - \tau_{\mathsf{V}}) S(t - \tau_{\mathsf{V}}) \\ \dot{I}_{\mathsf{V}}(t) &= r_{\mathsf{V}} V(t - \tau_{\mathsf{V}}) S(t - \tau_{\mathsf{V}}) \\ \dot{I}_{\overline{\mathsf{F}}}(t) &= r_{\mathsf{V}} V(t) S(t) - r_{\mathsf{V}} V(t - \tau_{\mathsf{F}}) S(t - \tau_{\mathsf{F}}) \\ \dot{I}_{\mathsf{F}}(t) &= r_{\mathsf{V}} V(t - \tau_{\mathsf{F}}) S(t - \tau_{\mathsf{F}}) \\ \dot{P}(t) &= r_{\mathsf{F}} F(t) S(t) + p_{\mathsf{P}} P(t) \\ \dot{F}(t) &= v_{\mathsf{F}} I_{\mathsf{F}}(t) - d_{\mathsf{F}} F(t) \\ \dot{V}(t) &= v_{\mathsf{V}} I_{\mathsf{V}}(t) - d_{\mathsf{V}} V(t). \end{aligned} \tag{1.6}$$

The corresponding initial value problem of our model is defined by the DDE system (1.6) for time $t \geq 0$ together with initial conditions, which we specify next. At the starting

time $t_0 = 0$ only two initial values are unequal to zero, namely the number of susceptible cells $S(0) = S_0$ as well as the extracellular viral load $V(0) = V_0$. Additionally, we must provide history functions for those variables in our system (1.6), which have retarded arguments and refer to the past $[t_0 - \tau_m, t_0]$ for $m \in \{$R, V, F$\}$. Since we will simulate experiments that start with the initial infection at time $t_0 = 0$, we must set $V(t) = 0$ for $t < 0$. This implies, however, that the choice of the history function for the number of susceptible cells S is irrelevant for the solution of the DDE system (1.6), as the delayed variable $S(t - \tau_m)$ with $\tau_m > 0$ is always multiplied by $V(t - \tau_m)$ and $V(t - \tau_m) = 0$ for $t < \tau_m$. For simplicity, we take $S(t) = 0$ for $t < 0$.

To solve the DDE system (1.6) we utilize the freely available RADAR5 solver written in ANSI Fortran-90 [S3]. The RADAR5 framework comprises an algorithm to calculate numerically the solution of stiff delay-differential equations based on an adapted 3-stage Radau IIA collocation method [S4, S5, S6]. This algorithm corresponds to a certain implicit Runge-Kutta method of order 5 in which the Runge-Kutta equations are solved by means of a suitable Newton process [S7]. The Newton iterations require two Jacobian matrices, first the standard Jacobian matrix $\mathcal{J}$ and, second, the Jacobian matrix with respect to the retarded variables $\mathcal{J}_\tau$. These Jacobian matrices are either computed internally or can be provided by the user. In order to increase the accuracy of the numerical solution, we implemented both time-dependent Jacobian matrices of our DDE system (1.6) within the RADAR5 framework.

In addition to the RADAR5 solver we also tested Matlab's dde23 solver for delay-differential equations with constant delays [S8, S9]. Although the implementation in Matlab is more convenient and modifications of the model can be achieved with less effort than within the RADAR5 framework, the significantly faster calculation in Fortran makes the RADAR5 framework more suitable for the purpose of parameter estimation, which will be detailed next.

2 Parameterization of the model based on wildtype DENV data

For the parameterization of the model (1.6), we focus on the time-resolved flow cytometry data set along with ELISA quantification of IFN-λ after infection of the IFIT1deGFP reporter cells with DENV-faR (cf. **Fig 6B, C**). This data set was obtained by measuring a specified proportion of the cell suspensions and thus provides absolute numbers of virus replicating and IFN responding cells with respect to the measured volume.

At the beginning of the experiments, the IFIT1deGFP reporter cells were infected at a MOI of 0.1 $TCID_{50}$/cell. Based on the definition of MOI given by

$$\text{MOI} = \frac{V_0}{S_0}, \tag{2.7}$$

and the initial number of naïve cells S_0 ~15000 in the relevant experiments, we set the initial extracellular viral load $V_0 \overset{(2.7)}{=} 0.1 \times 15000 = 1500$ arbitrary units/ml. This speci-

fication implies, that the viral load related infection rate r_{V} can be determined up to a scaling factor.

In addition, we take advantage of an independent virus stability experiment to obtain an estimate of the virus degradation rate constant d_{V} (**S7C Fig**). The DENV-faR stability analysis was performed by incubating IFN-incompetent BHK-21 cells with a high number of DENV-faR particles i.e., the BHK-21 cells were infecting and the cell culture medium was not changed. The extracellular amount of virus was identified by TCID_{50} assay over time. Since the production of new infectious virus particles in host cells takes approximately 16 h (cf. **Fig 4C** and **S7B Fig**), the measured extracellular amount of virus decreased continuously between 0 and 12 h after incubation. According to the shape of the observed extracellular virus kinetic $\mathcal{V}(t)$, we suppose an exponential virus decay and consider as objective function

$$\mathcal{V}(t) \quad = \quad \mathcal{V}_0 \, e^{-d_{\mathsf{V}} t} \tag{2.8}$$

for $t \in \mathbb{R}$ with the initial condition $\mathcal{V}(0) = \mathcal{V}_0$. To fit the parameters of the objective function (2.8) to the virus stability data, we utilized the trust-region-reflective least-squares algorithm of Matlab's optimization toolbox [S10]. After applying the optimization method with 10^4 different random initial values, the best fit yielded d_{V} = 0.4/h and provides a satisfying match with the data (cf. **S7C Fig**, blue curve).

Moreover, we found that fitting of the IFN secretion rate v_{F} and the rate constant of IFN degradation d_{F} at once would lead to a correlation of both parameters, and thus we set d_{F} = 0.15/h [S11].

To determine the remaining model parameters, we use the time-resolved data set consisting of two independent flow cytometry measurements along with ELISA quantifications post DENV-faR infection (cf. **Fig 6B, C**) by comparing the following observed kinetics with the related model readouts (ROs):

(RO1) The total number of cells A serves as a control and is described in our model by

$$A(t) \quad = \quad S(t) + I_{\overline{m}}(t) + I_m(t) + P(t), \tag{2.9}$$

at time $t \in \mathbb{R}$ and $m \in \{\mathsf{R, V, F}\}$.

(RO2) The number of experimentally identified IFIT1deGFP – DENV-faR double-negative cells are represented by $\widetilde{S}(t)$ defined as

$$\widetilde{S}(t) \quad = \quad S(t) + I_{\overline{\mathsf{R}}}(t). \tag{2.10}$$

$\widetilde{S}(t)$ comprises both susceptible cells and already infected but not yet virus replicating cells $I_{\overline{\mathsf{R}}}$, which implies that the DENV-faR fluorescence protein is not yet visible in the host cell.

(RO3) The observed DENV-faR positive cells correspond to the calculated number of virus replicating cells $I_{\mathsf{R}}(t)$.

(RO4) The detected ISG expressing IFIT1deGFP$^+$ – DENV-faR$^-$ cells are modeled by the protected cells $P(t)$.

(RO5) Since IFN-λ is the most prominent type of IFN in our cell system, we equate the measured level of secreted IFN-λ with the simulated amount of released IFN $F(t)$.

To exploit both independently performed experiments, the model dynamics listed in (RO2)-(RO5) are simultaneously fitted to the related time course data of experiment 1 and 2 by allowing only the initial number of susceptible cells S_0 to vary between experiments, while all other parameters are identical.

As optimization method we use the trust-region-reflective least-squares algorithm of Matlab's optimization toolbox [S10] by calling the Fortran program of our DDE system with a binary Matlab executable (mex) subroutine. Since we compare several components of the model with their respective data, the chi-squares statistic χ^2 of the model parameters θ_k, $k = 1, \dots, N_\text{p}$, is given by

$$\chi^2(\theta_1, ..., \theta_{N_\text{p}}) \;=\; \sum_{j=1}^{N_\text{c}} \sum_{i=1}^{N_\text{t}} \left(\frac{d_j(t_i) - y_j(t_i \mid \theta_1, \dots, \theta_{N_\text{p}})}{\sigma_j(t_i)} \right)^2, \tag{2.11}$$

where N_p stands for the number of model parameters and N_c denotes the number of model components $y_j(t_i \mid \theta_1, \dots, \theta_{N_\text{p}})$ that describe the associated data set $(t_i, d_j(t_i))$ with the measurement error $\sigma_j(t_i)$ for $i = 1, \dots, N_\text{t}$ and $j = 1, \dots, N_\text{c}$ at N_t observation times. To account for the fact, that the data used for fitting were obtained via different measurement techniques and comprise small as well as large values, we assume for the measurement errors a 10$\%$ deviation band relative to the measured kinetic data: $\sigma_j(t_i) = 0.1\, d_j(t_i)$. To find a reliable best fit parameter set of our optimization problem

$$\min_{\{\theta_k \mid k=1,\dots,N_\text{p}\}} \left(\chi^2(\theta_1, \dots, \theta_{N_\text{p}}) \right), \tag{2.12}$$

the least-squares minimization has to be repeated for a large number of different random initial values. It turned out that the calculation of the DDE system (1.6) is considerably more time consuming than solving comparable large ODE systems. To speed up calculation, we utilized Matlab's parallel computing toolbox to run several optimization processes simultaneously on a computer cluster. Due to parallelization we were able to repeat the optimization procedure for more than 10^4 different random initial values. The resulting best fit correctly reproduces the observed kinetic data of both experiments (**Fig 6C**).

In order to assess the uncertainties of the best fit parameter values, we calculate likelihood-based confidence intervals for the estimated parameters by applying the profile-likelihood method [S12]. Likelihood-based confidence regions dependent on a threshold in the likelihoods $Q_{\chi^2}(1 - a, \mathit{DF})$, which represents the $(1 - a)$ quantile of the χ^2-distribution with *DF* degrees of freedom. As we aim to compute the confidence bound of each individual parameter, the degree of freedom in this case is equal to 1.

The $(1-a)$ confidence interval (CI) of a single parameter θ_l, $l \in \{1,\ldots,N_\mathsf{p}\}$, encloses a set of parameter values ν and is defined as

$$\mathsf{CI}^{\theta_l}_{(1-a)} \;=\; \{\nu \mid \Delta\chi^2_{\theta_l}(\nu) \le Q_{\chi^2}(1-a,1)\}, \tag{2.13}$$

with

$$\begin{aligned} \Delta\chi^2_{\theta_l}(\nu) \;=\; & \min_{\{\theta_k | k=1,\ldots,N_\mathsf{p}; k\neq l\}} \left(\chi^2(\theta_1,\ldots,\theta_{l-1},\nu,\theta_{l+1},\ldots,\theta_{N_\mathsf{p}})\right) \\ & - \min_{\{\theta_k | k=1,\ldots,N_\mathsf{p}\}} \left(\chi^2(\theta_1,\ldots,\theta_{N_\mathsf{p}})\right). \end{aligned} \tag{2.14}$$

To compute

$$\min(\chi^2_{\theta_l \mathsf{fix}}) \coloneqq \min_{\{\theta_k | k=1,\ldots,N_\mathsf{p}; k\neq l\}} \left(\chi^2(\theta_1,\ldots,\theta_{l-1},\nu,\theta_{l+1},\ldots,\theta_{N_\mathsf{p}})\right) \tag{2.15}$$

from equation (2.14), we fix the parameter θ_l, $l \in \{1,\ldots,N_\mathsf{p}\}$ systematically to different values ν around the estimated optimum and solve the minimization problem with respect of the remaining parameters $\{\theta_k \mid k = 1,\ldots,N_\mathsf{p}; k \neq l\}$. Afterwards, the term $\Delta\chi^2_{\theta_l}(\nu)$ (cf. (2.14)) is obtained by subtracting the fitted χ^2-value minimized over all parameters (2.12) from the calculated $\min(\chi^2_{\theta_l \mathsf{fix}})$-value. The most common confidence region $\mathsf{CI}_{95\%}$ follows from equation (2.13) by taking into account the corresponding quantile of the χ^2-distribution $Q_{\chi^2}(95\%,1) = 3.8$. By plotting $\Delta\chi^2_{\theta_l}(\nu)$ and detecting those values ν for which $\Delta\chi^2_{\theta_l}$ remain below the respective quantile-based thresholds, the confidence intervals can be determined directly from the graph of the profile likelihood.

The application of the profile-likelihood method for our model regarding the parameters that were estimated based on the kinetic wildtype DENV data set shown in **Fig 6C**, reveals that all these parameters are identifiable within narrow confidence bounds (**Fig 7A and S1 Table**). Since the model is constrained by experimental wildtype DENV data, we can utilize it to make quantitative predictions about the competition between spreading DENV and IFN-induced antiviral protection.

To validate the parameterization, we examined whether key parameter values agree with the results of independent measurements. As described in the main text, we found that the estimated time delays of virus replication (cf. **Fig 5**) and virus production (cf. **Fig 7B**) comply with experimental data that were not used for model fitting.

In addition to the time delays, we also investigated the parameterized IFN response in the model using an IFN stimulation experiment, in which A549-IFIT1deGFP reporter cells were treated with IFN-λ (cf. **S3B Fig**). To imitate the initial conditions of this experiment, we consider a number of susceptible cells S_0 along with the applied extracellular IFN concentration F_0 = 10 ng/ml. When simulating the IFN response, we assume that the recognition of extracellular IFN F turns susceptible cells S into protected cells P with the protection rate r_F. The removal of IFN by cellular uptake and, to a lesser

extent, by extracellular degradation is taken into account through the IFN degradation rate d_{F}. The dynamics of this small submodel at time $t \in \mathbb{R}_{\geqslant 0}$ is defined by the following ODE system:

$$\begin{aligned} \dot{S}(t) &= -r_{\mathsf{F}} F(t) S(t) & S(0) &= S_0 \\ \dot{P}(t) &= r_{\mathsf{F}} F(t) S(t) & P(0) &= 0 \\ \dot{F}(t) &= -d_{\mathsf{F}} F(t) & F(0) &= F_0. \end{aligned} \tag{2.16}$$

We fix S_0, r_{F} and d_{F} to the fitted values given in **S1 Table**. The ODE system (2.16) was then solved using a standard ODE solver of MATLAB based on explicit Runge-Kutta method [S13, S14]. The direct comparison shows a good agreement between the predicted fraction of protected cells and the observed IFN-responding cells (**Fig 7C**). This test indicates that the parameterized model describes the IFN response correctly and, additionally, underscores the accuracy of the estimated protection rate r_{F}.

Taken together, the validity of the model is corroborated by the parameterization within narrow confidence intervals from wildtype DENV data and, additionally, by the consistency with key results of independent experiments that were not considered for model fitting.

3 Detection of DENV mutant-specific parameters by utilizing the knowledge from wildtype fitting

Driven by the question, which antiviral factors have the greatest influence on viral fitness, we compared wildtype DENV with the E217A DENV mutant, which is unable to modify its RNA genome by 2'-O-methylation [S15]. Our studies show that the E217A mutant elicits faster IFN production resulting in an earlier onset of the IFN response, and barely spreads in IFN-competent cells (**Fig 8B, C**). In order to analyze the differences between wildtype and attenuated E217A mutant infections on a quantitative level, we utilize the population-based model parameterized exclusively with wildtype DENV data (cf. section 2) to identify E217A mutant-specific parameters.

Motivated by findings from the literature, we argue that four parameters might vary between wildtype DENV and E217A mutant infections. Since studies demonstrate that IFIT1 inhibits translation of mutant RNA [S16, S17], the generation of new infectious virus particles could take longer or occur at a lower rate in the E217A mutant. Moreover, as other reports show that 2'-O-unmethylated RNA is detected more readily by intracellular pattern recognition receptors [S18], infected cells might also produce IFN faster or to a larger extent.

For the parameter optimization regarding the E217A mutant, we therefore take advantage of the established wildtype DENV parameter set (cf. **S1 Table**) and estimate only the four just mentioned potentially E217A mutant-specific parameters (MP) in line with their respective constrains:

(MP1) Delay of mutant virus production $\tau_{\text{V mut}} > \tau_{\text{R}}$

(MP2) Mutant virus production rate $\upsilon_{\text{V mut}} \leq \upsilon_{\text{V}}$

(MP3) Delay of IFN secretion after mutant virus infection $\tau_{\text{F mut}}$

(MP4) IFN secretion rate after mutant virus infection $\upsilon_{\text{F mut}} \geq \upsilon_{\text{F}}$.

In the same way as in the wildtype DENV case, we determine the E217A mutant-specific parameters (MP1)-(MP4), by fitting the model dynamics listed in (RO2)-(RO5) (cf. section 2 page 5) simultaneously to the related time-resolved data set consisting of two independent flow cytometry measurements along with ELISA quantifications post DENV-faR E217A mutant infection (cf. **Fig 8B, C**).

We repeated the optimization procedure for more than 5×10^3 different random initial values in the same way as for the data concerning wildtype DENV. The obtained best fit provides a good match with the observed kinetics after DENV-faR E217A mutant infection of both experiments (cf. **Fig 8C**).

Again, applying the profile-likelihood method, we calculate the 95% confidence intervals of the estimated E217A mutant-specific parameters (MP1)-(MP4) according to (2.13). The resulting narrow confidence bounds confirm the identifiability of all four parameters. More importantly, the comparison of the confidence regions reveals that only two of the four E217A mutant-specific parameters differ strongly from their wildtype DENV values: The time delay of IFN secretion is ~24 h shorter and the virus production rate is ~8-fold lower after E217A mutant infection in contrast to wildtype DENV (**S2 Table and Fig 8D**).

4 Extension of the population-based model to elucidate the paracrine and autocrine effects of IFN

The prediction of the data-based quantitative model, that a decreased virus production and an accelerated IFN induction make the difference between wildtype DENV and the attenuated E217A mutant, raises the questions how much impact either factor individually has on the outcome of infection, or even if the two factors are possibly related to each other. The latter question is prompted by our observation that stimulation of infected cells with IFN in an early phase after infection causes a reduction of virus replication and consequently virus production (cf. **Fig 1A, B** and **S1 Fig**). However, this autocrine effect of IFN must be temporally limited, since stimulation with IFN has no further influence after a certain time period post infection.

To analyze the impact of IFN on infected cells, we expand our established model (1.6) by assuming that the recognition of IFN in a certain time window of IFN responsiveness τ_{P} after viral entry inhibits the production of new infectious virus particles (**Fig 9A**, solid green inhibition link). Technically, we calculate the probability of infected cells to sense IFN in the time period τ_{P} based on techniques from the field of the survival analysis

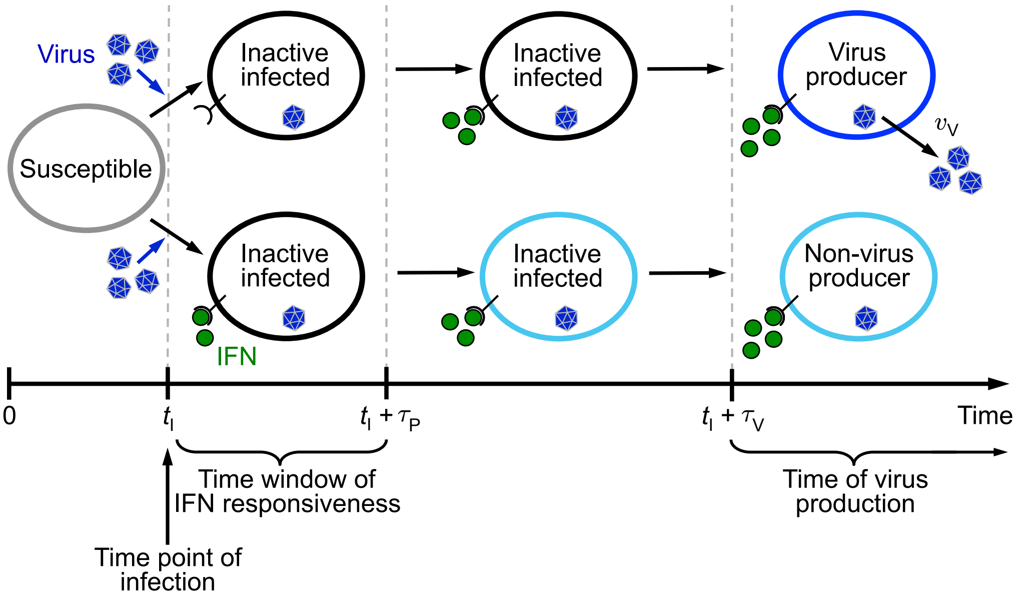

Mathematical Modeling Fig 1. Scheme of the modeled antiviral effect of IFN on already infected cells. To incorporate into the model an antiviral impact of IFN on infected cells, we assume after the time point of infection t_I of susceptible cells (gray oval) an IFN-responsive time window τ_P. Inactive infected cells (black oval), which receive no IFN stimulus in the time interval $[t_I; t_I + \tau_P]$ become at $t_I + \tau_V$ virus producing cells (dark blue oval) and release new infectious viral particles with the rate constant v_V (upper sequence). In contrast, virus production is inhibited in inactive infected cells, which recognize IFN in the time frame $[t_I; t_I + \tau_P]$ (light blue oval; lower sequence).

[S19, S20, S21].

At first, we consider a non-negative, continuous random variable T, which represents the waiting time until an infected cell recognizes IFN. The probability $\mathcal{P}$ to receive no IFN stimulus within the time span $\tilde{t} \in \mathbb{R}_{\geq 0}$ is defined as

$$\mathcal{P}(T > \tilde{t}) \underset{T \text{ non-negative}}{\overset{T \text{ continuous}}{=}} \exp\left(- \int_0^{\tilde{t}} h(x) dx \right), \tag{3.17}$$

where h denotes the so-called hazard function [S20]. In our case, the hazard function is equal to the time-dependent probability rate of recognizing IFN given by

$$h(t) = r_P F(t), \tag{3.18}$$

with the rate of IFN-induced inhibition of virus production r_P and the amount of extracellular IFN $F(t)$ (**Fig 9A**, solid green inhibition link).

The distinction whether or not an infected cell is stimulated by IFN within the time window τ_P after the time point of infection $t_I \geq 0$ (**Mathematical Modeling Fig 1**) is relevant at the time

$$\begin{aligned} t &= t_{\mathsf{I}} + \tau_{\mathsf{V}} \\ \iff \quad t - \tau_{\mathsf{V}} &= t_{\mathsf{I}}. \end{aligned} \tag{3.19}$$

Therefore, we compute the probability that IFN is not recognized up to the time

$$t_{\mathsf{I}} + \tau_{\mathsf{P}} \overset{(3.19)}{=} t - \tau_{\mathsf{V}} + \tau_{\mathsf{P}} \tag{3.20}$$

under the condition that there was no stimulation through IFN before the time point of viral entry. The condition is necessary, since sensing of IFN before infection leads to antiviral protection in our model. The probability of an infected cell to produce virus without antiviral IFN action is thus calculated as

$$\begin{aligned} \mathcal{P}(T > t_{\mathsf{I}} + \tau_{\mathsf{P}} \mid T > t_{\mathsf{I}}) &\underset{(3.19)}{\overset{(3.20)}{=}} \mathcal{P}(T > t - \tau_{\mathsf{V}} + \tau_{\mathsf{P}} \mid T > t - \tau_{\mathsf{V}}) \\ &= \frac{\mathcal{P}(T > t - \tau_{\mathsf{V}} + \tau_{\mathsf{P}} \cap T > t - \tau_{\mathsf{V}})}{\mathcal{P}(T > t - \tau_{\mathsf{V}})} \\ &= \frac{\mathcal{P}(T > t - \tau_{\mathsf{V}} + \tau_{\mathsf{P}})}{\mathcal{P}(T > t - \tau_{\mathsf{V}})} \\ &\overset{(3.17)}{=} \exp\left(- \int_0^{t-\tau_{\mathsf{V}}+\tau_{\mathsf{P}}} h(x)dx \right) \exp\left(\int_0^{t-\tau_{\mathsf{V}}} h(x)dx \right) \\ &\overset{(3.18)}{=} \exp\left(- \int_0^{t-\tau_{\mathsf{V}}+\tau_{\mathsf{P}}} r_{\mathsf{P}} F(x)dx \right) \exp\left(\int_0^{t-\tau_{\mathsf{V}}} r_{\mathsf{P}} F(x)dx \right) \\ &= \exp\left(-r_{\mathsf{P}} \int_{t-\tau_{\mathsf{V}}}^{t-(\tau_{\mathsf{V}}-\tau_{\mathsf{P}})} F(x)dx \right) \\ &= \exp\left(r_{\mathsf{P}} \left[\mathscr{F}(t - \tau_{\mathsf{V}}) - \mathscr{F}(t - (\tau_{\mathsf{V}} - \tau_{\mathsf{P}})) \right] \right), \end{aligned} \tag{3.21}$$

where $\mathscr{F}$ denotes the antiderivative of F.

The probability (3.21) enables us to discriminate between infected cells $I_{\mathsf{V}\overline{\mathsf{P}}}$, which receive no IFN stimulus and thus produce virus with the rate constant v_{V} after τ_{V}, and infected, non-virus producing cells I_{VP}, which recognize IFN within the time window τ_{P} (cf. **Mathematical Modeling Fig 1**). Accordingly, we include $I_{\mathsf{V}\overline{\mathsf{P}}}$, I_{VP} and $\mathscr{F}$ in our DDE model (1.6) and obtain the following DDE system with four time delays:

$$
\begin{aligned}
\dot{S}(t) &= -r_{\mathsf{V}}V(t)S(t) - r_{\mathsf{F}}F(t)S(t) + p_{\mathsf{S}}S(t) \\
\dot{I}_{\overline{\mathsf{R}}}(t) &= r_{\mathsf{V}}V(t)S(t) - r_{\mathsf{V}}V(t-\tau_{\mathsf{R}})S(t-\tau_{\mathsf{R}}) \\
\dot{I}_{\mathsf{R}}(t) &= r_{\mathsf{V}}V(t-\tau_{\mathsf{R}})S(t-\tau_{\mathsf{R}}) \\
\dot{I}_{\overline{\mathsf{V}}}(t) &= r_{\mathsf{V}}V(t)S(t) - r_{\mathsf{V}}V(t-\tau_{\mathsf{V}})S(t-\tau_{\mathsf{V}}) \\
\dot{I}_{\mathsf{V}\overline{\mathsf{P}}}(t) &= \exp\left(r_{\mathsf{P}}\left[\mathscr{F}_1(t) - \mathscr{F}_2(t)\right]\right) r_{\mathsf{V}}V(t-\tau_{\mathsf{V}})S(t-\tau_{\mathsf{V}}) \\
\dot{I}_{\mathsf{VP}}(t) &= \left(1 - \exp\left(r_{\mathsf{P}}\left[\mathscr{F}_1(t) - \mathscr{F}_2(t)\right]\right)\right) r_{\mathsf{V}}V(t-\tau_{\mathsf{V}})S(t-\tau_{\mathsf{V}}) \\
\dot{I}_{\overline{\mathsf{F}}}(t) &= r_{\mathsf{V}}V(t)S(t) - r_{\mathsf{V}}V(t-\tau_{\mathsf{F}})S(t-\tau_{\mathsf{F}}) \qquad (3.22) \\
\dot{I}_{\mathsf{F}}(t) &= r_{\mathsf{V}}V(t-\tau_{\mathsf{F}})S(t-\tau_{\mathsf{F}}) \\
\dot{P}(t) &= r_{\mathsf{F}}F(t)S(t) + p_{\mathsf{P}}P(t) \\
\dot{F}(t) &= v_{\mathsf{F}}I_{\mathsf{F}}(t) - d_{\mathsf{F}}F(t) \\
\dot{\mathscr{F}}_1(t) &= F(t-\tau_{\mathsf{V}}) \\
\dot{\mathscr{F}}_2(t) &= F(t-(\tau_{\mathsf{V}}-\tau_{\mathsf{P}})) \\
\dot{V}(t) &= v_{\mathsf{V}}I_{\mathsf{V}\overline{\mathsf{P}}}(t) - d_{\mathsf{V}}V(t).
\end{aligned}
$$

The associated initial value problem of the extended model is defined by the DDE system (3.22) for time $t \geq 0$ together with initial conditions, which we specify in the following. At the starting time $t_0 = 0$, only the number of susceptible cells $S(0) = S_0$ and the extracellular viral load $V(0) = V_0$ are non-zero. For those variables, that have retarded arguments, we use as history functions the constant zero function, that is, $S(t) = 0$, $V(t) = 0$ as well as $F(t) = 0$ for $t < 0$.

The full delay-differential equation model (3.22) is numerically solved by applying the RADAR5 solver written in ANSI Fortran-90 [S3]. To obtain a precise solution, we implemented the time-dependent standard Jacobian matrix and the Jacobian matrix with respect to the retarded variables within the RADAR5 framework.

The results of the IFN-stimulation experiment depicted in **Fig 1B** indicate an IFN responsive time window τ_{P} of approximately 6 h post infection with wildtype DENV at a MOI of 10 $TCID_{50}$/cell. The infection with such a high viral dose causes a synchronized infection of the entire cell population. Since the delay of virus replication after a non-synchronized infection is expected to be delayed, we simulate low dose infections with a time window of IFN responsiveness of $\tau_{\mathsf{P}} = 8$ h. Additionally, we fix the rate of IFN-induced inhibition of virus production $r_{\mathsf{P}} = 10^4\, r_{\mathsf{F}}$, as we suspect that restriction of virus replication is achieved faster than antiviral protection.

According to findings from the literature, IFIT1 is one of those ISGs that can be induced directly after viral recognition in an IFN-independent manner [S22, S23]. An early IFN-independent IFIT1 induction could thus also be contributing to the predicted decreased virus production in IFIT1 reporter cells after E217A mutant infection. To incorporate

IFN-independent antiviral effects in the model, we assume that virus producing cells release new infectious virus particles with a reduced rate $\upsilon_{\mathrm{V\,mut}}$ = 0.5 υ_{V} post DENV mutant infection, which represents half of the virus production rate υ_{V} after infection with wildtype DENV.

The full model (3.22) with the mentioned modifications listed in **S3 Table** matches the observed kinetics of both wildtype and mutant DENV replication and IFN response equally well as the original model (1.6). Moreover, the full model allows us to study the competition between spreading DENV and the antiviral immune response induced in an autocrine as well as paracrine manner by secreted IFN. In addition, we can separately analyze the effect of IFN on infected or naïve cells by setting either r_{P} or r_{F} to zero and subsequently compare the relative importance of both antiviral effects on a quantitative level (cf. main text and **Fig 9B**).

References

S1. Chang WS, Shang H, Perera RM, Lok Sm, Sedlak D, Kuhn RJ, Lee GU (2008) Rapid detection of Dengue virus in serum using magnetic separation and fluorescence detection. Analyst 133: 233–240.

S2. Hu J, Nudelman G, Shimoni Y, Kumar M, Ding Y, López C, Hayot F, Wetmur JG, Sealfon SC (2011) Role of cell-to-cell variability in activating a positive feedback antiviral response in human dendritic cells. PloS One 6: e16614.

S3. Guglielmi N, Hairer E (2005) Users guide for the code RADAR5. Appl Numer Math 52: 113–127.

S4. Hairer E, Wanner G (1999) Stiff differential equations solved by Radau methods. J Comput Appl Math 111: 93–111.

S5. Guglielmi N, Hairer E (2001) Implementing Radau IIA methods for stiff delay differential equations. Computing 67: 1–12.

S6. Guglielmi N, Hairer E (2008) Computing breaking points in implicit delay differential equations. Adv Comput Math 29: 229–247.

S7. Guglielmi N (2005) On the Newton iteration in the application of collocation methods to implicit delay equations. Appl Numer Math 53: 281–297.

S8. Shampine LF, Thompson S (2001) Solving DDEs in Matlab. Appl Numer Math 37: 441–458.

S9. Shampine LF, Thompson S (2009) Numerical solution of delay differential equations. Delay differential equations, Springer, 1–27.

S10. Coleman T, Zhang Y (2003) Optimization toolbox for use with Matlab. The MathWorks Inc .

S11. Rand U, Rinas M, Schwerk J, Nöhren G, Linnes M, Kröger A, Flossdorf M, Kály-Kullai K, Hauser H, Höfer T, Köster M (2012) Multi-layered stochasticity and paracrine signal propagation shape the type-I interferon response. Mol Syst Biol 8: 584.

S12. Venzon D, Moolgavkar S (1988) A method for computing profile-likelihood-based confidence intervals. Appl Statist 87–94.

S13. Bogacki P, Shampine LF (1989) A 3(2) pair of Runge-Kutta formulas. Appl Math Lett 2: 321–325.

S14. Shampine LF, Reichelt MW (1997) The Matlab ODE suite. SIAM J Sci Comput 18: 1–22.

S15. Ray D, Shah A, Tilgner M, Guo Y, Zhao Y, Dong H, Deas TS, Zhou Y, Li H, Shi PY (2006) West Nile virus 5'-cap structure is formed by sequential guanine N-7 and ribose 2'-O methylations by nonstructural protein 5. J Virol 80: 8362–8370.

S16. Pichlmair A, Lassnig C, Eberle CA, Górna MW, Baumann CL, Burkard TR, Bürckstümmer T, Stefanovic A, Krieger S, Bennett KL, Rülicke T, Weber F, Colinge J, Müller M, Superti-Furga G (2011) IFIT1 is an antiviral protein that recognizes 5'-triphosphate RNA. Nat Immunol 12: 624–630.

S17. Habjan M, Hubel P, Lacerda L, Benda C, Holze C, Eberl CH, Mann A, Kindler E, Gil-Cruz C, Ziebuhr J, Thiel V, Pichlmair A (2013) Sequestration by IFIT1 impairs translation of 2'O-unmethylated capped RNA. PLoS Pathog 9: e1003663.

S18. Züst R, Cervantes-Barragan L, Habjan M, Maier R, Neuman BW, Ziebuhr J, Szretter KJ, Baker SC, Barchet W, Diamond MS, Siddell SG, Ludewig B, Thiel V (2011) Ribose 2'-O-methylation provides a molecular signature for the distinction of self and non-self mRNA dependent on the RNA sensor Mda5. Nat Immunol 12: 137–143.

S19. Cox DR, Oakes D (1984) Analysis of survival data, vol. 21. CRC Press.

S20. Dobson AJ (2001) An introduction to generalized linear models. CRC Press.

S21. Rodríguez G (2007). Lecture notes on generalized linear models. Princeton University.

S22. Grandvaux N, Servant MJ, tenOever B, Sen GC, Balachandran S, Barber GN, Lin R, Hiscott J (2002) Transcriptional profiling of interferon regulatory factor 3 target genes: direct involvement in the regulation of interferon-stimulated genes. J Virol 76: 5532–5539.

S23. Diamond MS, Farzan M (2013) The broad-spectrum antiviral functions of IFIT and IFITM proteins. Nat Rev Immunol 13: 46–57.
