# Supplementary figures and images for: Live Cell Analysis and Mathematical Modeling Identify Determinants of Attenuation of Dengue Virus 2’-O-Methylation Mutant
Source: PLoS Pathog. 2015 Dec 31;11(12):e1005345. doi: 10.1371/journal.ppat.1005345 (PMC4697809; doi:10.1371/journal.ppat.1005345)

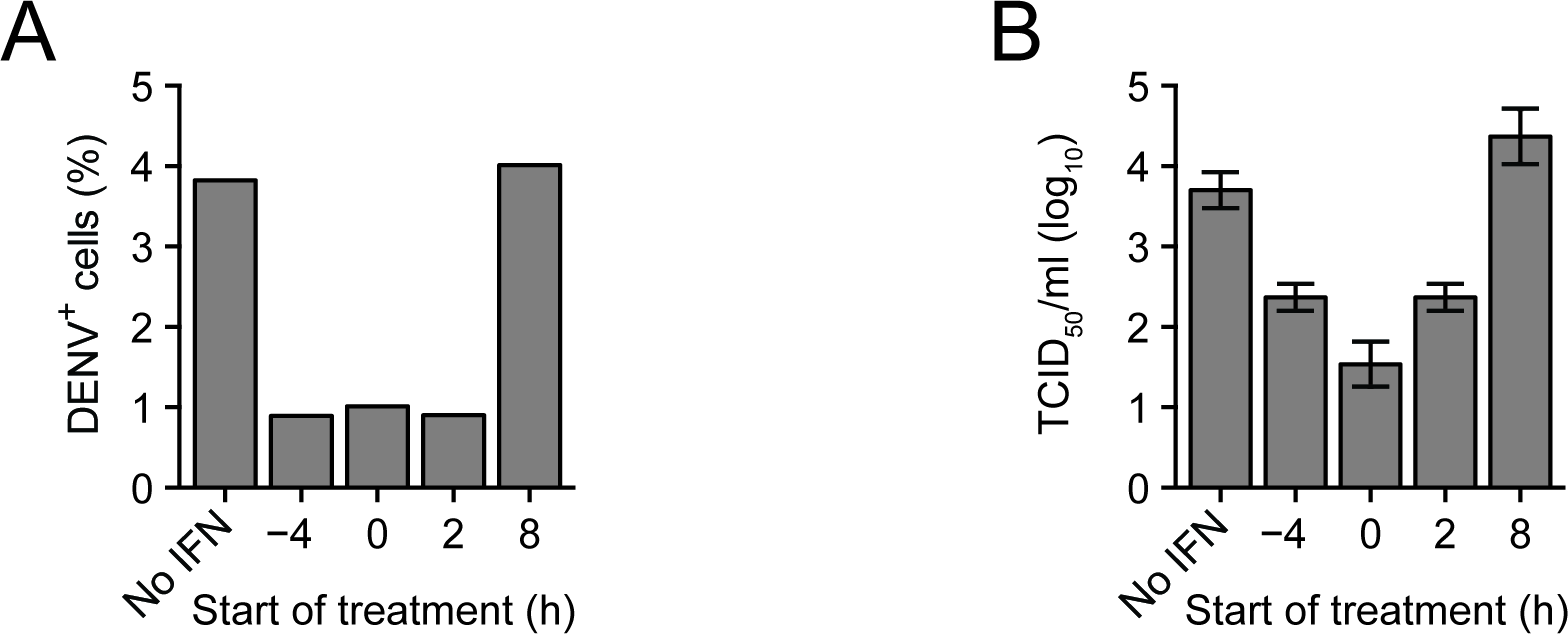

Supplement: S1 Fig — IFN-competent A549 cells were treated with 100 IU/ml IFN-α prior to (-4 h) or after (2 h, 8 h) or at the time point of infection (0 h) with the DENV2 strain 16681 at a MOI of 0.1 TCID50/cell. Cells were fixed 28 h post infection and analyzed by immunofluorescence using a NS5-specific antiserum. Shown is a representative experiment (n = 2). Mock-treated and DENV-infected cells without IFN treatment served as reference. (A) Quantification of infection efficiency. For each time point, 250–350 cells detected in 3 view fields were analyzed for DENV infection. (B) Titers of infectious supernatants harvested from the cells shown in panel (A) were determined by limiting-dilution assay. (TIF) [file ppat.1005345.s002.tif]

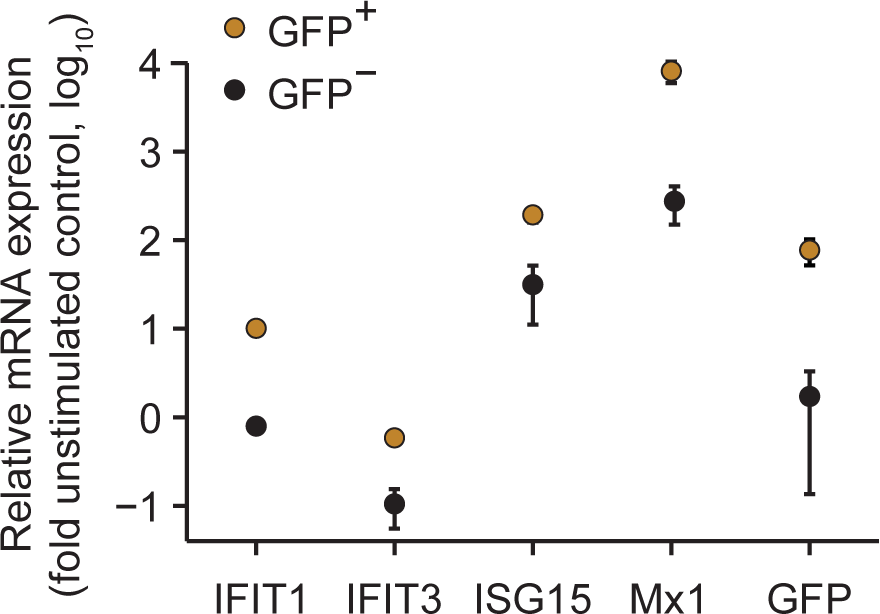

Supplement: S2 Fig — A549-Mx1deGFP cells were stimulated with 10 IU/ml IFN-α for 24 h and sorted according to deGFP expression by using flow cytometry. Directly after sorting, GFP-positive and -negative cells were lysed and total RNA was extracted. Amounts of the mRNAs specified in the bottom of the graph were quantified by RT-qPCR and normalized to GAPDH mRNA levels. Data are the mean from two independent experiments and their respective SDs. (TIF) [file ppat.1005345.s003.tif]

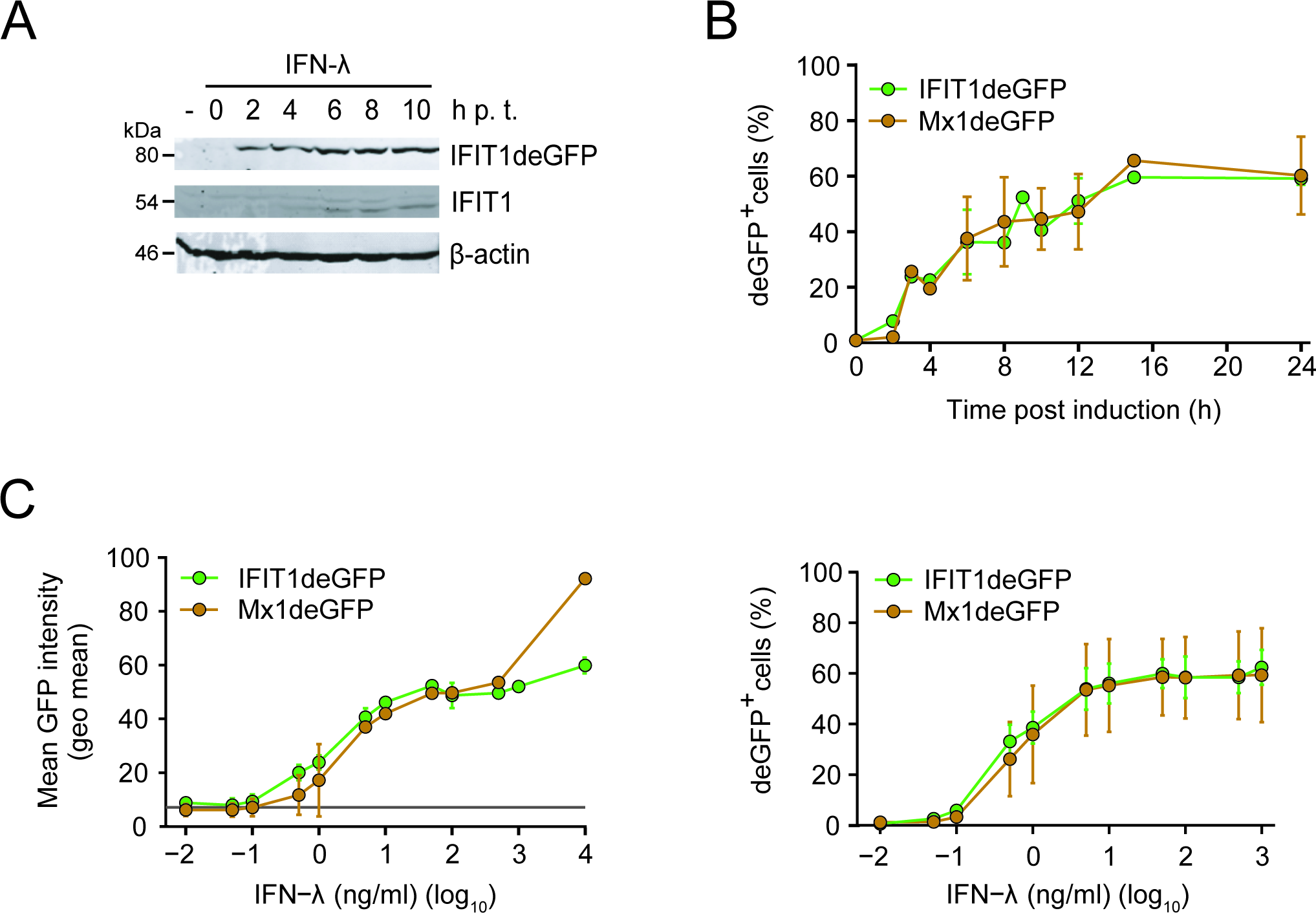

Supplement: S3 Fig — (A) A549-IFIT1deGFP cells were stimulated with 10 ng/ml IFN-λ. Cells were harvested at time points specified in the top (hours) and lysates were analyzed by Western blot using mono-specific antisera (GFP, IFIT1 and β-actin, top to bottom, respectively). A representative immunoblot of 3 independent experiments is shown. (B) Induction kinetics of IFIT1deGFP and Mx1deGFP after treatment of A549 reporter cells with 10 ng/ml IFN-λ. Cells were fixed at time points specified in the bottom and number of GFP-positive cells was determined by flow cytometry. Shown are the mean and SD of 2 independent experiments. (C) Dose response assay for IFN-λ. Cells were treated with various concentrations of IFN-λ that are specified in the bottom and 24 h later mean GFP intensity was determined by flow cytometry (left panel; grey line indicates detection limit). The number of GFP-expressing cells (right panel) was determined in the analogous way. Data are means from 3 independent experiments and their respective SDs. (TIF) [file ppat.1005345.s004.tif]

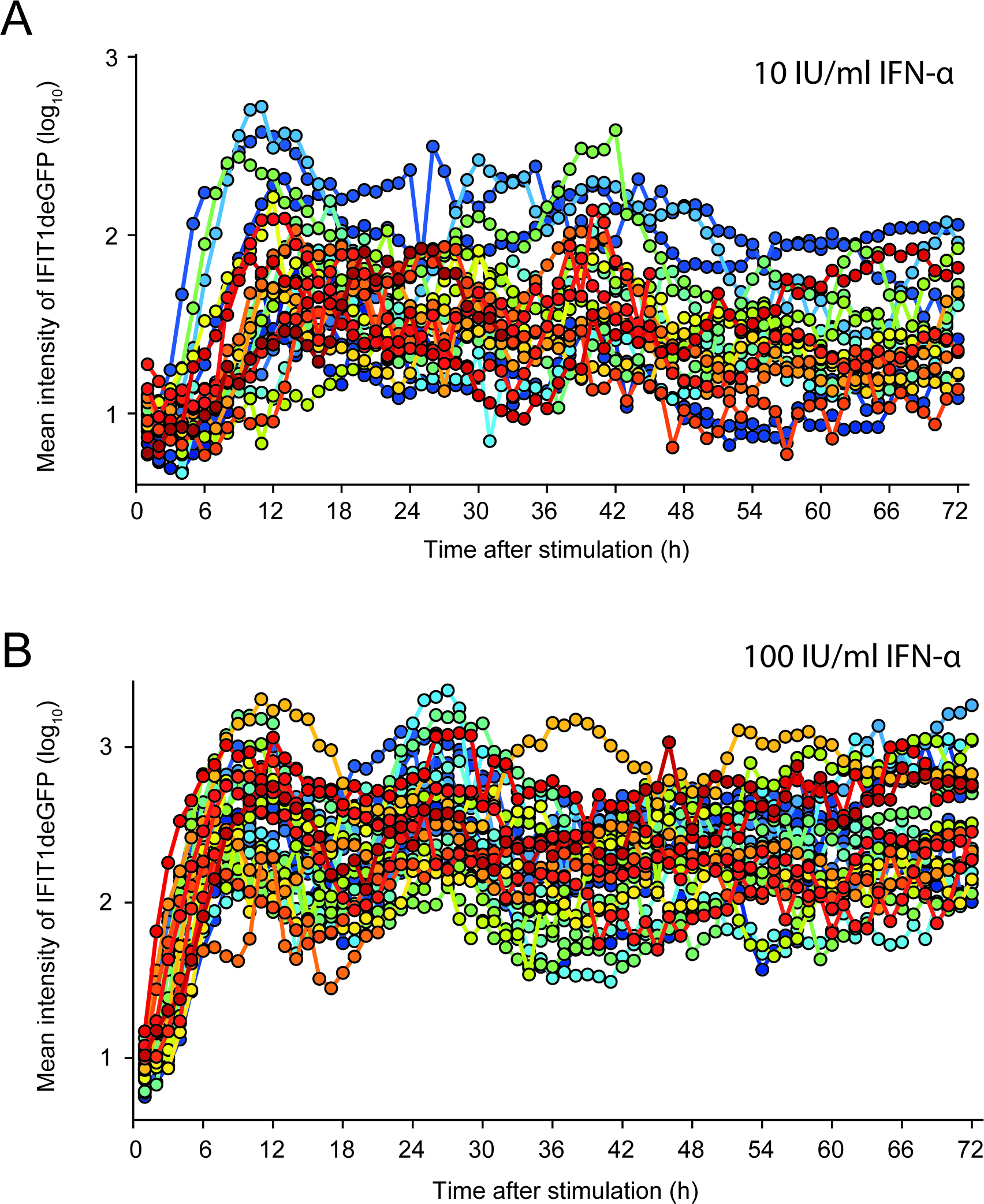

Supplement: S4 Fig — A549-IFIT1deGFP reporter cells were treated with 10 IU/ml (A) or 100 IU/ml (B) IFN-α and monitored by time-lapse microscopy for 72 h. Mean intensity of the IFIT1deGFP reporter was quantified in single cells by automated image analysis as described in the materials and methods section. (TIF) [file ppat.1005345.s005.tif]

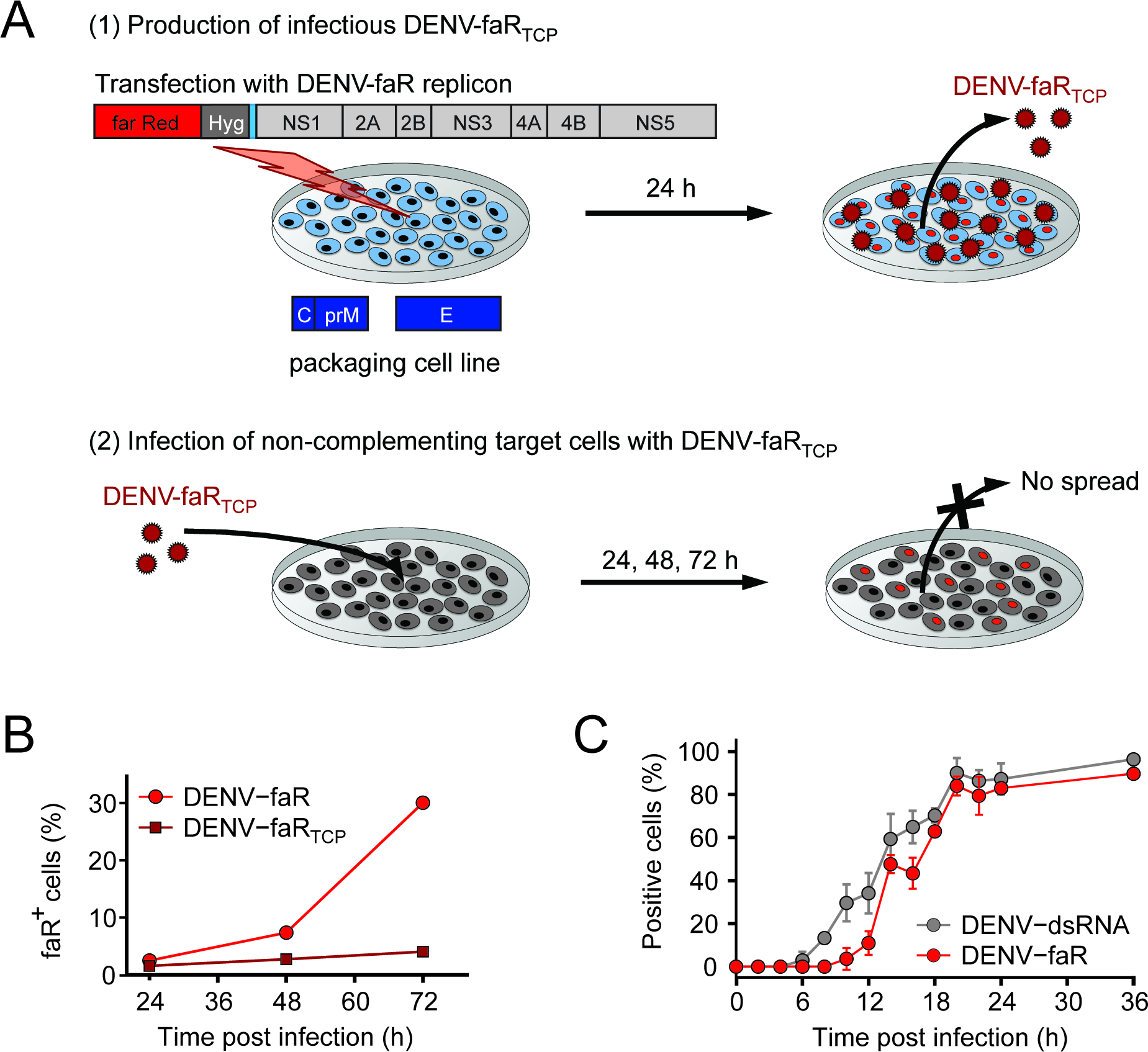

Supplement: S5 Fig — (A) Schematic of the DENV-faR trans-complemented particle (TCP) system. (1) Infectious DENV-faRTCP was produced by transfecting cells that stably express capsid protein—prM and E (for reasons of biosafety two independent expression constructs had to be used) with a subgenomic DENV-faR reporter replicon RNA. This replicon contains the faR reporter gene and lacks C, prM and E that are provided in trans in the engineered helper cell line. DENV-faRTCPs (dark red circles) released into the cell culture supernatant were harvested 24 h after transfection and used to infect naïve cells. Infected cells can be detected via monitoring faR expression. (2) DENV-faRTCP is unable to spread in cells that do not express the structural proteins. These cells support only infection and replication, but not virus particle production and virus spread, hence the name “single round infection”. (B) Comparison of DENV spread in naïve A549 cells upon infection with the DENV-faR reporter virus or DENV-faRTCPs. Cells were infected at a MOI of 0.1 TCID50/cell and the fraction of faR-positive cells was detected by flow cytometry at time points specified in the bottom. (C) Kinetics of DENV-faR reporter virus replication and spread in A549 cells. Upon infection with DENV-faR at a MOI of 10 TCID50/cell, the number of DENV-faR- and DENV-dsRNA-positive cells detectable at given time points was determined by immunofluorescence assay using faR- and dsRNA-specific antibodies. Data are means from 2 independent experiments and their respective SDs. (TIF) [file ppat.1005345.s006.tif]

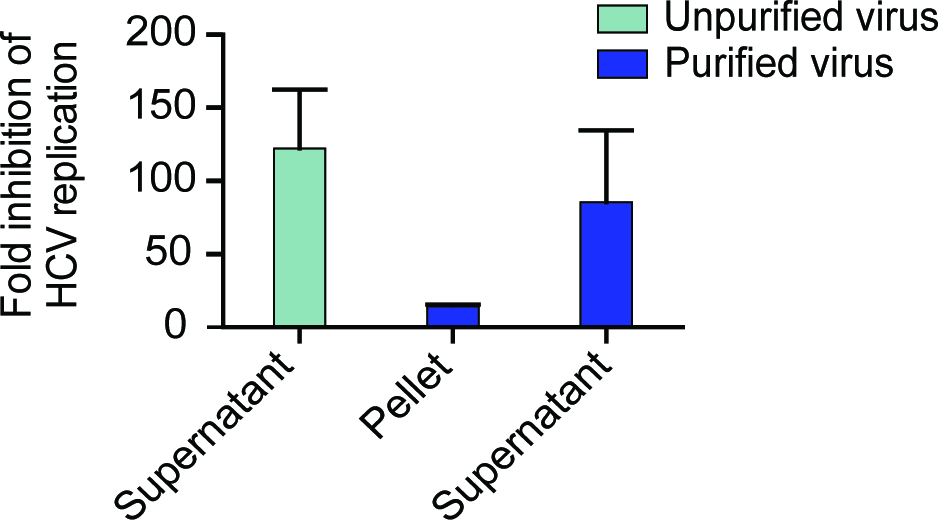

Supplement: S6 Fig — Absence of antiviral cytokines in DENV-faR virus stocks produced on VeroE6 cells after purification by sucrose gradient centrifugation. Aliquots of unpurified culture supernatants of DENV-infected VeroE6 cells as well as virus pellets and supernatants after ultracentrifugation were added to HCV replicon-containing cells. These replicons are highly sensitive to IFNs and thus, suitable to measure even low amounts of antiviral cytokines [78]. To avoid DENV-mediated lysis of HCV replicon-containing cells, samples were subjected to UV inactivation prior to inoculation of the cells. HCV RNA replication was determined by luciferase assay. (TIF) [file ppat.1005345.s007.tif]

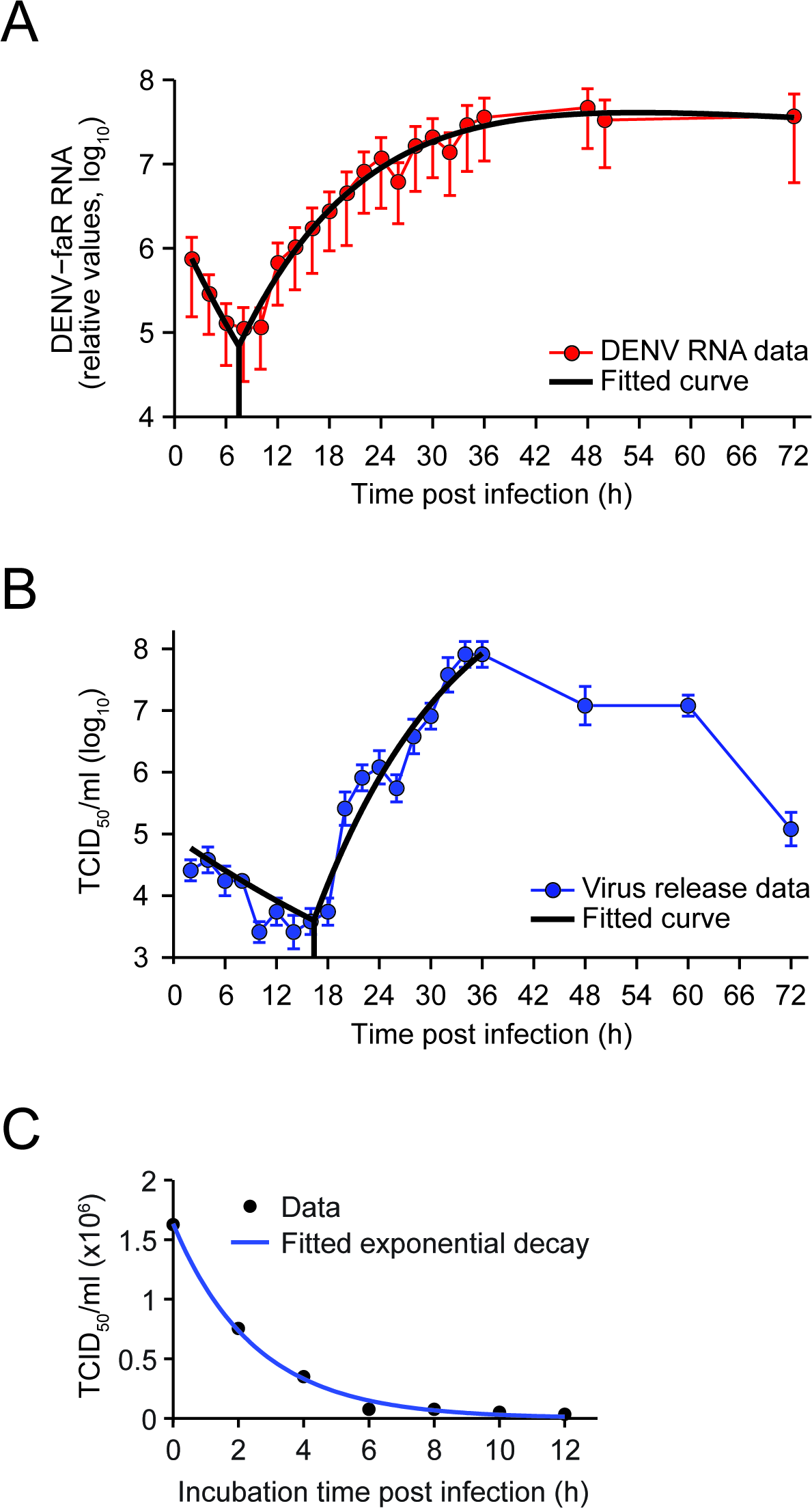

Supplement: S7 Fig — To determine the initiation of viral RNA replication and virus particle release, we fitted the measured kinetics of (A) DENV-faR RNA and (B) infectious virus concentration after high dose infection (data replotted from Fig 4C) in log10 space using the following objective function f i with i ∈{RNA, Virus}: fi(t)= fi,0 e− dit+H(t−ti,on) fi,max (1−e− ri(t−ti,on)). This function accounts for an exponential decay of the initial amount f i,o with a rate constant d i at time t. The replication and virus production setting in at time t i,on after infection are described with a Heaviside step function H multiplied by a first order kinetic that increases with rate r i and saturates at f i,max. The shown best fit is obtained by applying a trust-region-reflective least-squares algorithm with at least 2 × 10^3^ different random initial values. In the case of fitting the TCID50 data, we only considered data points measured in the time range from 2 to 36 h p.i. In addition, we calculated the 95% confidence intervals of the parameter values using a non-parametric bootstrap method with sample size 5 × 10^4^ leading to the following results (given in best fit [lower and upper bound of 95% confidence interval]): (A) f RNA,0 = 106.3[105.5;108.9] relative values, d RNA = 0.04[0.01;0.1] 1/h, t RNA,on = 7.5[5.5;9.0] h, f RNA,max = 107.3[105.3;107.8] relative values, r RNA = 0.05[0.04;0.1] 1/h. (B) f Virus,0 = 105.0[104.5;106.4] TCID50/ml, d Virus = 0.02[0.01;0.05] 1/h, t Virus,on = 16.3[12.3;17.7] h, f Virus,max = 108.6[104.4;1076.3] TCID50/ml and r Virus = 0.05[0.004;0.21] 1/h. (C) Estimation of the virus degradation rate. The parameter determination of the rate constant of virus degradation d V is based on a virus stability experiment in which BHK-21 cells were incubated with DENV-faR virus particles and virus titers (black dots) were quantified by TCID50 assay at the indicated time points post incubation. Data were fitted with an exponential decay equation (blue curve) by applying a trust- [file ppat.1005345.s008.tif]

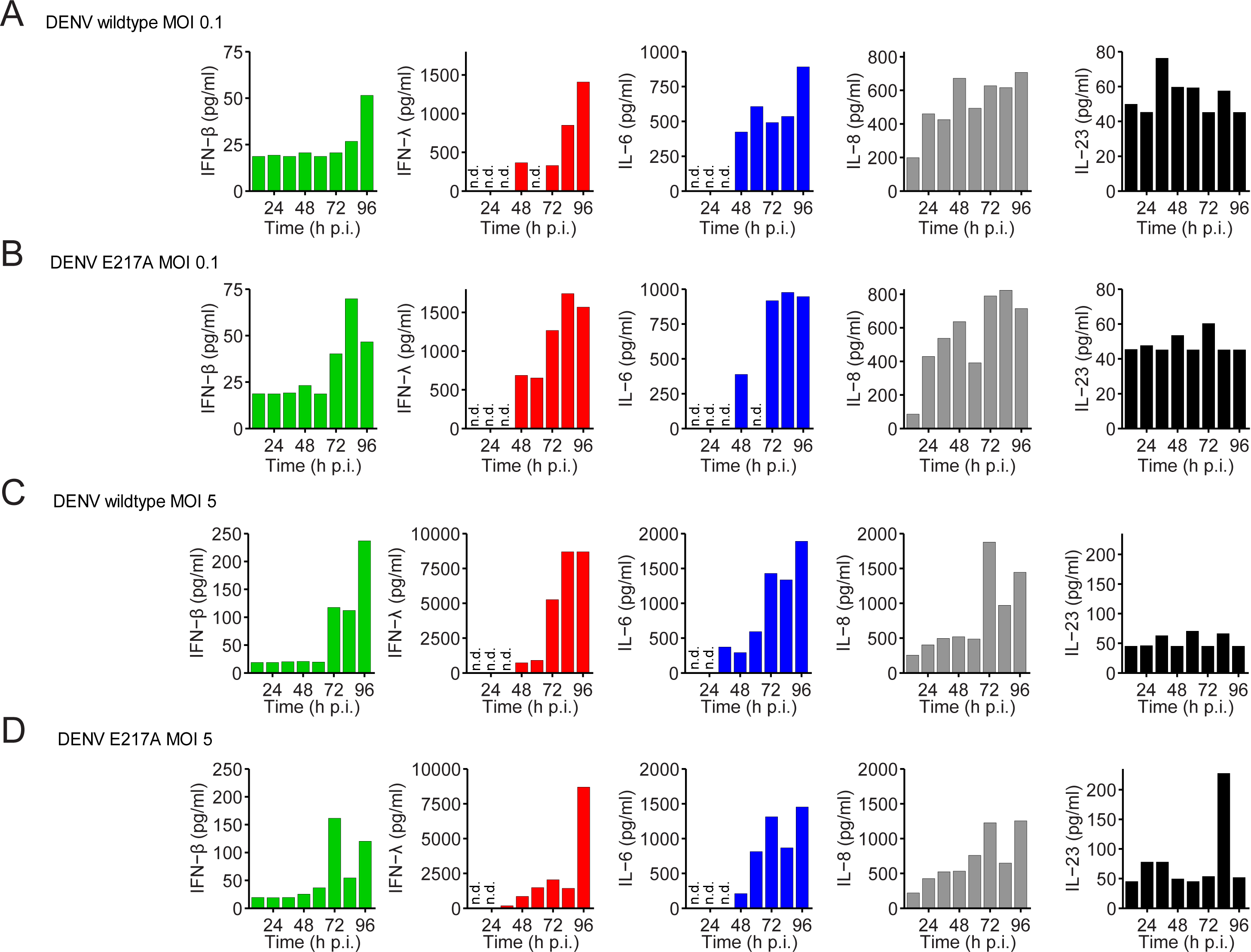

Supplement: S8 Fig — A549 cells were infected with wildtype DENV-faR or the DENV-faR E217A mutant at MOIs of 0.1 (A, B) or 5 (C, D) TCID50/cell, respectively. Supernatants were harvested in 12 h time intervals up to 96 h. Cytokine release was analyzed by using the VeriPlex Human Cytokine Multiplex ELISA kit (16-plex). Note that at MOI 0.1, IFN-λ levels induced by the mutant were higher than that of the wildtype. At MOI 5, IFN-λ levels induced by the mutant exceeded the wildtype-induced levels for early time points (≤ 60 h), but this was reversed at 72 h and 84 h whereas IFN-α measured in parallel was not detected. At both low and high MOI, the spread of the mutant virus was very much diminished. IL, interleukin; n.d., not detectable. (TIF) [file ppat.1005345.s009.tif]

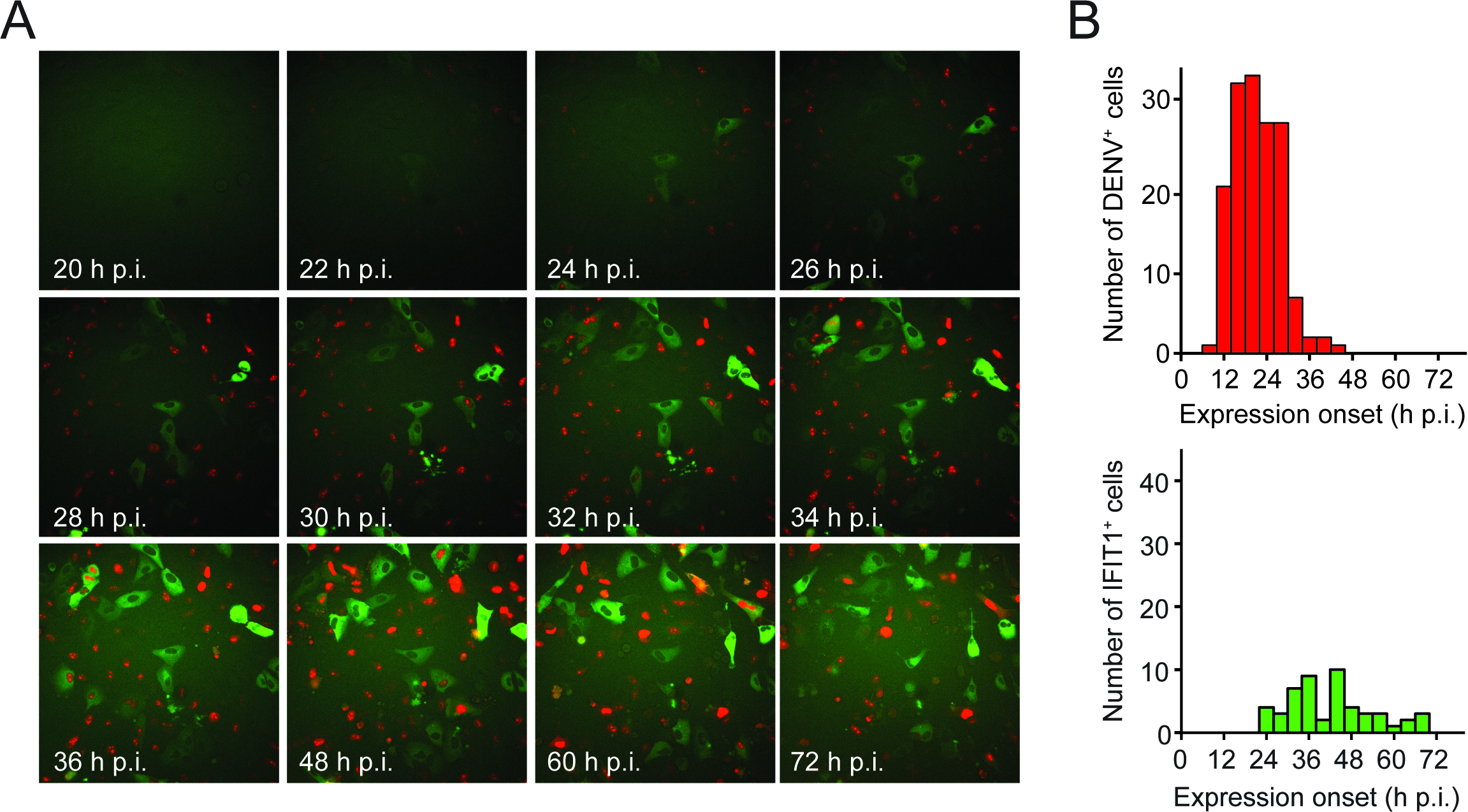

Supplement: S9 Fig — A549 reporter cell lines were infected with DENV-faR at a MOI of 10 TCID50/cell and time-lapse live cell imaging was started one hour later. Images were recorded in 1 h intervals until 72 h p.i. Images were analyzed by using the ImageJ software package and the MTrackJ plug-in. (A) Representative still images taken at time points specified in the bottom left. (B) Kinetics of onset of detectable expression of the viral faR reporter gene (upper panel) and the ISG-reporter IFIT1deGFP (lower panel), respectively. In panel B, 161 DENV+ and 51 IFIT1+ cells were used for the analysis, respectively. (TIF) [file ppat.1005345.s010.tif]

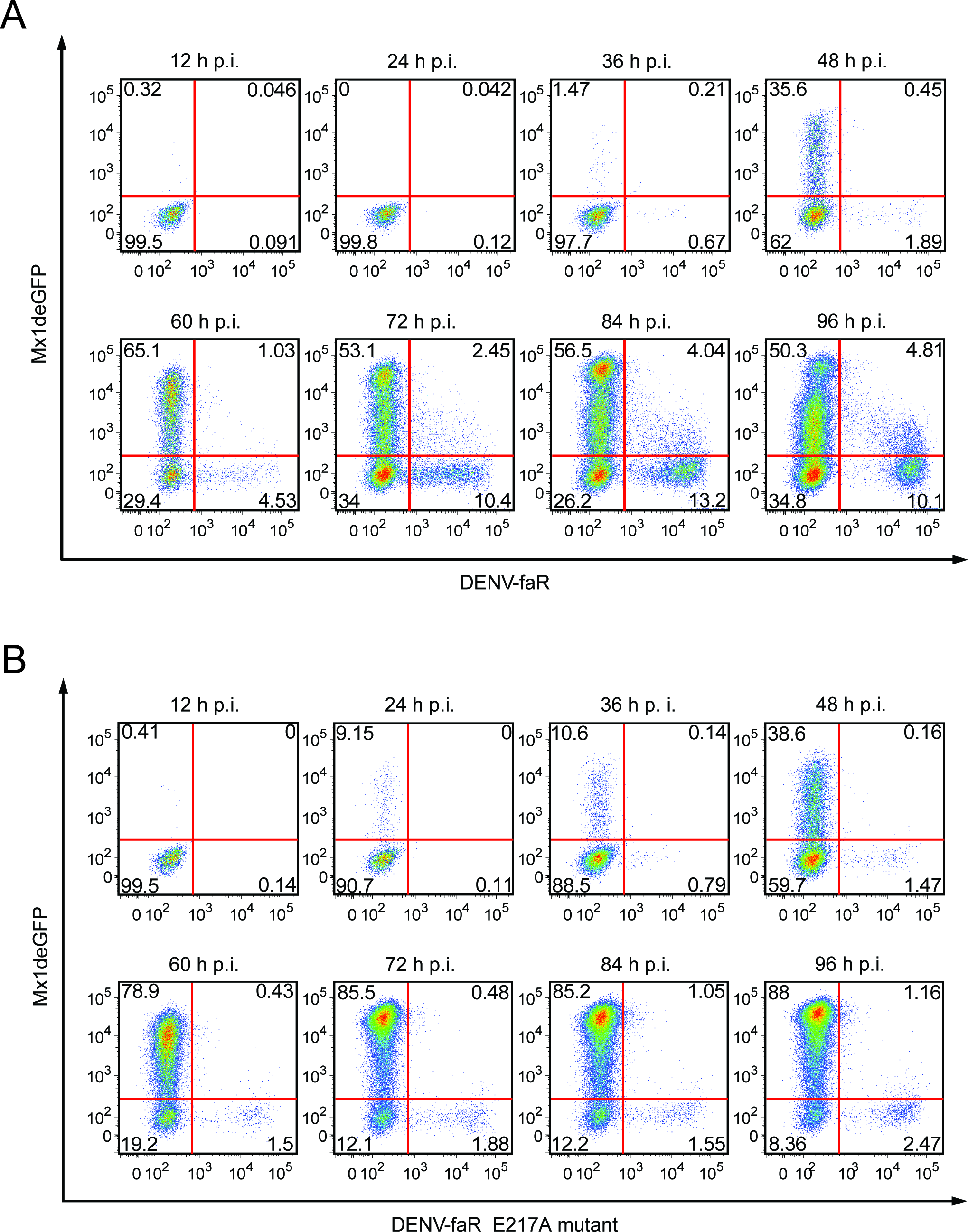

Supplement: S10 Fig — A549-Mx1deGFP reporter cells were infected with DENV-faR (A) or the DENV-faR E217A mutant (B) at a MOI of 0.1 TCID50/cell, respectively. At given time points post infection (p.i.), cells were fixed and 100 μl of the cell suspension was analyzed by flow cytometry. The dot plots illustrate the jointly measured DENV-faR (x-axis) and Mx1deGFP (y-axis) fluorescence intensities of individual cells. Shown is one of two independent experiments. (TIF) [file ppat.1005345.s011.tif]

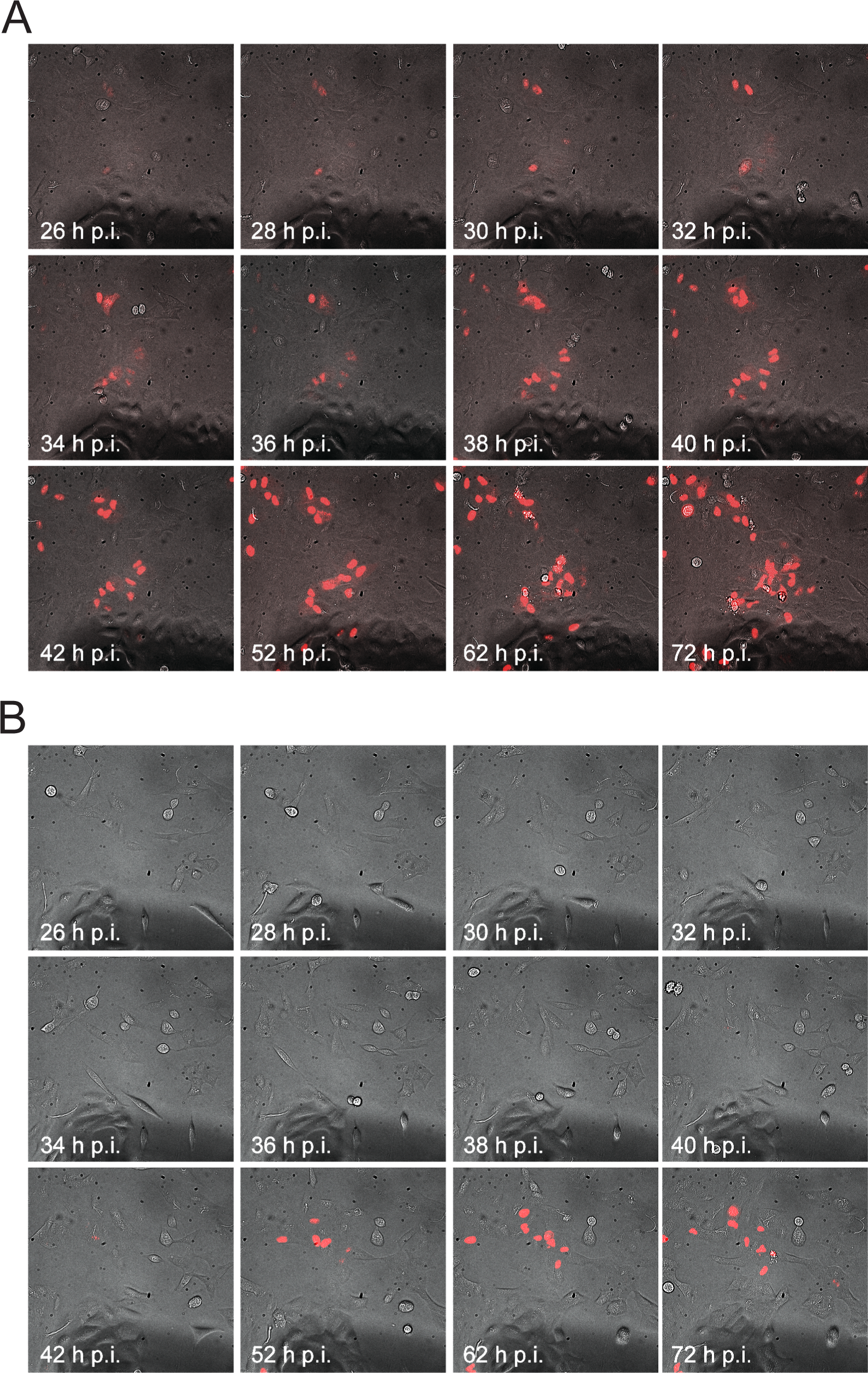

Supplement: S11 Fig — A549 cells were infected with DENV-faR wildtype (A) or the E217A mutant (B) at a MOI of 0.2 TCID50/cell and monitored by time-lapse microscopy for 72 h. Images were recorded in 30 min intervals. Representative still images taken at time points specified in the bottom left are shown. Images correspond to S3 and S4 Movies, respectively. Shown is an overlay of brightfield and fluorescence images. (TIF) [file ppat.1005345.s012.tif]

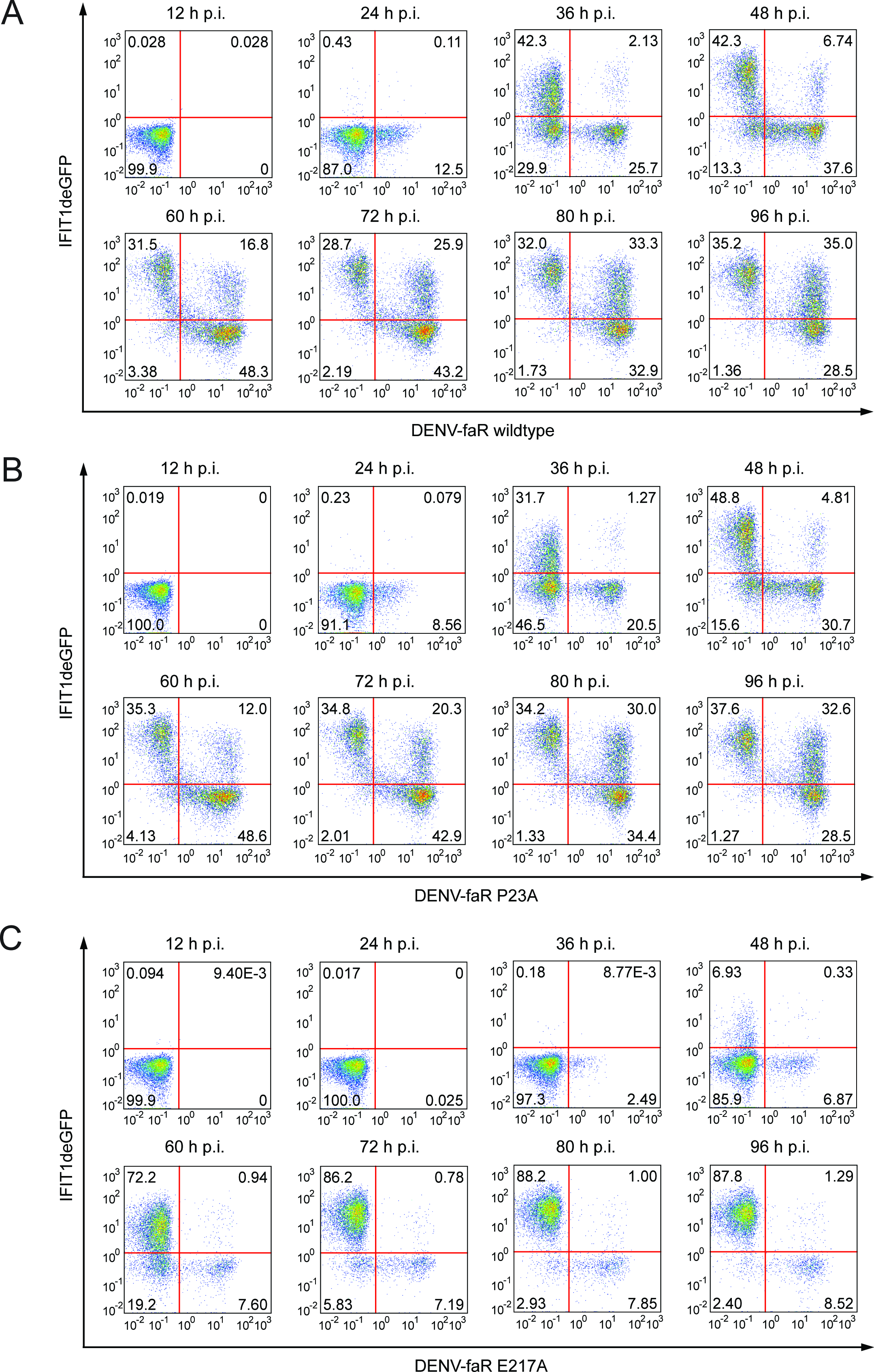

Supplement: S12 Fig — A549-IFITdeGFP cells were infected at a MOI of 5 TCID50/cell with (A) DENV-faR wildtype, (B) the P23A NS4B mutant [61] or (C) the E217A NS5 mutant, respectively. Cells were harvested 12, 24, 36, 48, 60, 72, 80 and 96 h post infection, fixed and analyzed by flow cytometry. A representative experiment is shown (n = 2). (TIF) [file ppat.1005345.s013.tif]
