# Supplementary material for: Live Cell Analysis and Mathematical Modeling Identify Determinants of Attenuation of Dengue Virus 2’-O-Methylation Mutant
Source: PLoS Pathog. 2015 Dec 31;11(12):e1005345. doi: 10.1371/journal.ppat.1005345 (PMC4697809; doi:10.1371/journal.ppat.1005345)
Supplement: S1 Table — According to the biological meaning (cf. Fig 6A and S1 Supplementary Methods, section 1), the model parameters were estimated by considering only data regarding wildtype DENV infection. Given are the best fit values and the 95% confidence intervals (calculated with the profile-likelihood method) after fitting the model to the time-resolved data set shown in Fig 6C using a fixed initial viral load along with separately determined values for virus and IFN degradation rates. Abbreviations: arbitrary units (a.u.), confidence interval (CI), experiment (exp.), hours (h), milliliter (ml), picogram (pg). (DOCX) [file ppat.1005345.s014.docx]

**S1 Table. Model parameter estimates based on wildtype DENV data.**

| **Model parameter** |  | **Value [95% confidence interval]** |
| --- | --- | --- |
| **Virus dynamics** |  |  |
| Initial viral load | *V*_0_ | 1500 a.u./ml |
| Delay of virus replication | *τ*_R_ | 24.0 [23.9; 24.1] h |
| Delay of virus production | *τ*_V_ | 31.4 [30.6; 32.2] h |
| Virus production rate | *v*_V_ | 0.54 [0.45; 0.67] a.u./h/cell |
| Infection rate | *r*_V_ | 1.7×10^-5^ [1.5×10^-5^; 2.0×10^-5^] ml/a.u./h |
| Virus degradation rate | *d*_V_ | 0.4/h |
| **IFN dynamics** |  |  |
| Delay of IFN secretion | *τ*_F_ | 31.9 [31.4; 32.5] h |
| IFN secretion rate | *v*_F_ | 0.014 [0.012; 0.016] pg/h/cell |
| Protection rate | *r*_F_ | 1.5×10^-5^ [1.3×10^-5^; 1.8×10^-5^] ml/pg/h |
| IFN degradation rate | *d*_F_ | 0.15/h |
| **Cell numbers** |  |  |
| Initial susceptible cells (exp.1) | *S*_0_ | 12540 [10760; 14460] cells/ml |
| Initial susceptible cells (exp.2) | *S*_0_ | 11720 [10210; 13330] cells/ml |
| Proliferation rate susceptible cells | *p*_S_ | 0.041 [0.038; 0.044] 1/h |
| Proliferation rate protected cells | *p*_P_ | 0.083 [0.078; 0.087] 1/h |
