# Supplementary material for: Live Cell Analysis and Mathematical Modeling Identify Determinants of Attenuation of Dengue Virus 2’-O-Methylation Mutant
Source: PLoS Pathog. 2015 Dec 31;11(12):e1005345. doi: 10.1371/journal.ppat.1005345 (PMC4697809; doi:10.1371/journal.ppat.1005345)
Supplement: S2 Table — Using the established wildtype DENV-related parameter values (cf. S1 Table), data concerning E217A mutant infection shown in Fig 8C were fitted by allowing only four parameters to differ between wildtype DENV and the E217A mutant. Given are the best fit values and the 95% confidence intervals (calculated with the profile-likelihood method). Abbreviations: arbitrary units (a.u.), confidence interval (CI), E217A mutant (mut), hours (h), picogram (pg). (DOCX) [file ppat.1005345.s015.docx]

**S2 Table. Model parameter estimates based on E217A mutant data.**

| **Model parameter** |  | **Value [95% confidence interval]** |
| --- | --- | --- |
| **Virus dynamics** |  |  |
| Delay of virus production (E217A mutant) | *τ*_V mut_ | 28.3 [26.8; 29.7] h |
| Virus production rate (E217A mutant) | *v*_V mut_ | 0.067 [0.06; 0.074] a.u./h/cell |
| **IFN dynamics** |  |  |
| Delay of IFN secretion (E217A mutant) | *τ*_F mut_ | 8.2 [7.5; 9.1] h |
| IFN secretion rate (E217A mutant) | *v*_F mut_ | 0.014 [0.014; 0.015] pg/h/cell |
