# Supplementary material for: Live Cell Analysis and Mathematical Modeling Identify Determinants of Attenuation of Dengue Virus 2’-O-Methylation Mutant
Source: PLoS Pathog. 2015 Dec 31;11(12):e1005345. doi: 10.1371/journal.ppat.1005345 (PMC4697809; doi:10.1371/journal.ppat.1005345)
Supplement: S3 Table — The full version of the model (cf. S1 Supplementary Methods, section 4; Figs 9 and 10) was simulated with the given parameter values and for the remaining parameters we used the estimates listed in S1 and S2 Tables. Abbreviations: arbitrary units (a.u.), E217A mutant (mut), hours (h), milliliter (ml), picogram (pg). (DOCX) [file ppat.1005345.s016.docx]

**S3 Table. Additional model parameters of the full model.**

| **Model parameter** |  | **Value** |
| --- | --- | --- |
| **Virus dynamics** |  |  |
| Virus production rate | *v*_V_ | 0.54 a.u./h/cell |
| Virus production rate (E217A mutant) | *v*_V mut_ | 0.27 a.u./h/cell |
| **IFN dynamics** |  |  |
| Time window of IFN responsiveness | *τ*_P_ | 8 h |
| Rate of IFN-induced inhibition of virus production | *r*_P_ | 0.15 ml/pg/h |
